# Effect of Apatinib Plus Pegylated Liposomal Doxorubicin vs Pegylated Liposomal Doxorubicin Alone on Platinum-Resistant Recurrent Ovarian Cancer: The APPROVE Randomized Clinical Trial

**DOI:** 10.1001/jamaoncol.2022.2253

**Published:** 2022-06-30

**Authors:** Tiantian Wang, Jie Tang, Hongying Yang, Rutie Yin, Jingru Zhang, Qi Zhou, Ziling Liu, Lanqin Cao, Li Li, Yi Huang, Kui Jiang, Wei Wang, Fenglin She, Ni Guan, Zhiguo Hou, Ning Li, Lingying Wu

**Affiliations:** 1 Department of Gynecologic Oncology, National Cancer Center/National Clinical Research Center for Cancer/Cancer Hospital, Chinese Academy of Medical Sciences and Peking Union Medical College, Beijing, China; 2 Department of Gynecologic Oncology, Hunan Cancer Hospital, Central South University, Changsha, China; 3 Department of Gynecology, The Third Affiliated Hospital of Kunming Medical University, Yunnan Provincial Cancer Center, Kunming, China; 4 Department of Obstetrics and Gynecology, West China Second University Hospital, Sichuan University, Chengdu, China; 5 Department of Gynecology, Cancer Hospital of China Medical University, Liaoning Cancer Hospital & Institute, Shenyang, China; 6 Department of Gynecologic Oncology, Chongqing University Cancer Hospital, Chongqing, China; 7 Department of Oncology Center, The First Hospital of Jilin University, Changchun, China; 8 Department of Gynecology, Xiangya Hospital, Central South University, Changsha, China; 9 Department of Gynecologic Oncology, Guangxi Medical University Affiliated Tumor Hospital, Nanning, China; 10 Department of Gynecologic Oncology, Hubei Cancer Hospital, Wuhan, China; 11 Department of Oncology, The Second Affiliated Hospital of Dalian Medical University, Dalian, China; 12 Department of Gynecology, Peking Union Medical College Hospital, Beijing, China; 13 Jiangsu Hengrui Pharmaceuticals Co Ltd, Shanghai, China

## Abstract

**Importance:**

There are substantial unmet therapeutic needs in patients with platinum-resistant recurrent ovarian cancer (PROC), and novel therapeutic strategies should be explored.

**Objective:**

To evaluate the efficacy and safety of treatment with apatinib (a vascular endothelial growth factor receptor 2 tyrosine kinase inhibitor) plus pegylated liposomal doxorubicin (PLD) for PROC.

**Design, Setting, and Participants:**

The APPROVE trial was performed as an open-label, randomized clinical trial at 11 hospitals in China between March 22, 2018, and November 16, 2020. Patients with histologically confirmed ovarian cancer who had experienced disease progression during or within 6 months of discontinuing any prior line of treatment with platinum-based chemotherapy were eligible. This primary analysis was based on data that were current as of January 28, 2021.

**Interventions:**

Patients received PLD alone (40 mg/m^2^, intravenously, every 4 weeks, for up to 6 cycles) or PLD plus apatinib (250 mg, orally, daily).

**Main Outcomes and Measures:**

The primary end point was progression-free survival (PFS) by Response Evaluation Criteria in Solid Tumours (RECIST), version 1.1, in the intent-to-treat population.

**Results:**

In total, 152 female patients were randomized, with 78 (51.3%) in the apatinib plus PLD group (median age, 54 years; range, 22-76 years) and 74 (48.7%) in the PLD group (median age, 56 years; range, 33-72 years). The median follow-up duration was 8.7 months (IQR, 4.7-14.1 months). The median PFS was 5.8 months (95% CI, 3.8-8.8) for treatment with apatinib plus PLD vs 3.3 months (95% CI, 2.1-3.8) for PLD (hazard ratio, 0.44; 95% CI, 0.28-0.71; *P* < .001). The median overall survival was 23.0 months (95% CI, 18.9 to not reached) with treatment with apatinib plus PLD vs 14.4 months (95% CI, 12.1-23.4) with PLD (hazard ratio, 0.66; 95% CI, 0.40-1.09). The most frequent grade 3 or higher treatment-emergent adverse events were decreased neutrophil counts (11 [14.9%] in the apatinib plus PLD group vs 6 [8.3%] in the PLD group), hypertension (6 [8.1%] vs none), and decreased white blood cell count (5 [6.8%] vs 3 [4.2%]). Two patients receiving treatment with apatinib plus PLD experienced grade 2 fistulas.

**Conclusions and Relevance:**

This randomized clinical trial found that treatment with apatinib plus PLD showed promising efficacy and manageable toxic effects in patients with PROC and may be a new alternative treatment option in this setting.

**Trial Registration:**

Clinicaltrials.gov Identifier: NCT04348032

## Key Points

**Question:**

Does the addition of apatinib, a vascular endothelial growth factor receptor 2 tyrosine kinase inhibitor, to treatment with pegylated liposomal doxorubicin (PLD) improve progression-free survival in patients with platinum-resistant recurrent ovarian cancer?

**Findings:**

In the APPROVE randomized clinical trial that included 152 patients with platinum-resistant recurrent ovarian cancer, the median progression-free survival in the apatinib plus PLD group was 5.8 months compared with 3.3 months in the PLD alone group.

**Meaning:**

The trial results found that apatinib plus PLD may be a novel alternative treatment option for patients with platinum-resistant recurrent ovarian cancer.

## Teaser

This randomized clinical trial examines the efficacy and safety of treatment with apatinib plus pegylated liposomal doxorubicin in Chinese patients with platinum-resistant recurrent ovarian cancer.

## Introduction

Ovarian cancer (OC) is one of the most common gynecological cancers, with 313 959 newly diagnosed cases and 207 252 deaths in women worldwide predicted in 2020; of these, 17.6% of new cases and 18.1% of deaths occur in China.^1^ Because of atypical early symptoms, more than 70% of patients receive a diagnosis at an advanced stage.^2^ Currently, for patients with a new diagnosis, cytoreductive surgery combined with platinum-based chemotherapy (with or without bevacizumab) is the standard treatment. Almost all patients will experience relapse and eventually develop platinum resistance. In this setting, monotherapy with pegylated liposomal doxorubicin (PLD), docetaxel, paclitaxel, or topotecan remains the predominant therapeutic option but results in a remarkably short survival, reflecting substantial unmet therapeutic needs.^2^

Angiogenesis plays a role in normal ovarian physiology and the progression of OC via ascites formation and metastatic spread, and its inhibition has been shown to have potential clinical benefit for patients with OC, including those with platinum-resistant disease.^3^ The AURELIA trial showed that the addition of bevacizumab (a humanized anti-vascular endothelial growth factor [VEGF] monoclonal antibody) to conventional non–platinum-based chemotherapy prolonged median progression-free survival (PFS) by 3.3 months and increased the objective response rate (ORR) by 15.5% compared with monochemotherapy, although adding bevacizumab to single-agent chemotherapy did not statistically improve overall survival (OS; hazard ratio [HR], 0.85; 95% CI, 0.66-1.08; *P* < .17).^4^ Another anti-VEGF agent, pazopanib, has also shown preliminary evidence of efficacy in combination with weekly paclitaxel for advanced platinum-resistant or refractory disease, but the clinical application was limited by toxic effects.^5,6^

Apatinib is an oral small-molecule tyrosine kinase inhibitor (TKI) that highly selectively binds to and inhibits VEGF receptor 2, the clinical efficacy of which has been proven in hepatocellular carcinoma and gastric cancer.^7,8^ For platinum-resistant or platinum-refractory OC, the encouraging antitumor activity and manageable toxic effects of treatment apatinib plus etoposide has been reported in the AEROC trial, with an ORR of 54% and median PFS of 8.1 months in 35 patients.^9^ However, there was no direct comparison to determine the actual contribution of apatinib in AEROC. The APPROVE randomized clinical trial was conducted to evaluate the efficacy and safety of treatment with apatinib in combination with PLD compared with PLD alone in patients with PROC.

## Methods

### Study Design and Participants

The multicenter, open-label, randomized clinical APPROVE trial was conducted at 11 hospitals in China (Supplement 1, Supplement 2, and eTable 1 in Supplement 3). Patients were eligible if they were 18 years or older; had histologically confirmed epithelial OC, primary peritoneal cancer, or fallopian tube cancer; had experienced disease progression during or within 6 months after discontinuing any prior line of treatment with platinum-based chemotherapy; had malignant pleural effusion or ascites or at least 1 assessable lesion according to Response Evaluation Criteria in Solid Tumours (RECIST; version 1.1); had an Eastern Cooperative Oncology Group performance status of 0 to 1; had a life expectancy of at least 4 months; and had adequate hematological, liver, and kidney function.

Key exclusion criteria included uncontrollable hypertension or arrhythmias, a history of cardiac insufficiency of grade 2 or greater, abnormal coagulation function, the presence of substantial clinical bleeding symptoms or a definite bleeding tendency within 3 months before randomization, major surgical procedures within 4 weeks before randomization, and a history of liposomal doxorubicin therapy within the last 6 months. The full eligibility criteria are listed in the trial protocol (Supplement 1).

The trial was approved by the ethics committee of each participating site and performed in accordance with the Declaration of Helsinki and Good Clinical Practice guidelines. All patients provided written informed consent. This trial followed the Consolidated Standards of Reporting Trials (CONSORT) reporting guideline.

### Randomization and Masking

Patients were randomly assigned (1:1) to receive treatment with either PLD alone or apatinib plus PLD via an interactive web response system and were stratified by platinum-free interval (PFI, ≤3 months vs 3-6 months [excluding the boundary values] from last receipt of platinum-based chemotherapy to progression) and prior platinum-sensitive relapse (yes vs no). Treatment was allocated in blocks of 4 or 6 in each stratum. Patients and investigators were not masked to the treatment allocation.

### Procedures

Treatment with PLD was administered intravenously at 40 mg/m^2^ on day 1, every 4 weeks, for up to 6 cycles in both groups. Apatinib was administered orally at a dose of 250 mg, once daily, in the apatinib plus PLD group. After the completion of 6 cycles of combination therapy, patients with disease control could receive maintenance therapy with apatinib until disease progression, unacceptable toxic effects, or withdrawal of consent. Dose reduction, interruption, and discontinuation were permitted. The detailed dose adjustment scheme is available in the trial protocol (Supplement 1).

Responses were assessed by the investigators per RECIST, version 1.1, using computed tomography or magnetic resonance imaging at baseline, every 2 cycles during treatment, and 4 weeks after discontinuing treatment. Laboratory parameters (including hematology parameters, prothrombin time, blood biochemistry parameters, routine urine/feces parameters, and the tumor marker CA-125) and vital signs were assessed, and 12-lead electrocardiograms were performed at baseline and before each cycle. Adverse events (AEs) were monitored before each cycle and within 30 days after the final administration of the study drug and were graded according to the National Cancer Institute Common Terminology Criteria for Adverse Events, version 4.0.

### Outcomes

The primary end point was PFS by RECIST, version 1.1, defined as the time from randomization to the first documented tumor progression or death. Secondary end points included OS (defined as the time from randomization until death of any cause), ORR (defined as the percentage of patients with at least 1 postbaseline efficacy assessment who achieved a complete or partial response [CR/PR] according to RECIST, version 1.1), disease control rate (DCR, defined as the percentage of patients with at least 1 postbaseline efficacy assessment who achieved a CR or PR or stable disease for at least 6 weeks according to RECIST, version 1.1), and safety.

### Statistical Analysis

In previously published data, the median PFS with treatment with PLD alone was approximately 3.0 months.^10,11^ We hypothesized that the addition of apatinib could improve the median PFS from 3.0 to 5.5 months. To achieve the 80% power needed to detect the difference in PFS between groups at a significance level of .05 (2-sided), 86 progression events or deaths were required. Assuming an enrolment period of 24 months and a total study period of 28 months, 126 patients would need to be enrolled, assuming a 20% dropout rate. With the outbreak of the COVID-19 epidemic, in-hospital follow-up was difficult because of epidemic control. To ensure statistical power, the APPROVE study group decided to adjust the sample size to 150 patients.

Efficacy analyses were conducted in the intent-to-treat population (defined as all patients who were randomized) for PFS and OS and in the modified intent-to-treat population (defined as all patients who were randomized and received at least 1 efficacy assessment after randomization) for the ORR and DCR. Safety analyses were performed in the safety population, defined as all patients who received at least 1 dose of study medication and received a documented safety assessment postadministration.

Progression-free survival and OS were estimated using the Kaplan-Meier method, and the corresponding 95% CIs were calculated with the Brookmeyer Crowley method. Hazard ratios and corresponding 95% CIs were estimated using a stratified Cox proportional hazards model. The time-to-event end points were compared between the 2 groups using the stratified log-rank test. For the ORR and DCR, 95% CIs were calculated using the Clopper-Pearson method. Post hoc subgroup analyses of PFS were conducted based on baseline characteristics, including age (<65 years, ≥65 years), Eastern Cooperative Oncology Group performance status (0, 1-2), previous platinum-sensitive relapse (yes, no), previous therapy lines (1, 2, ≥3), PFI (≤3 months, >3 months), ascites (yes, no), platinum-refractory disease (yes, no), and first platinum-resistant relapse (yes, no). The randomization stratification factors were included in all the stratified statistical models and tests as strata. Statistical analyses were conducted using SAS (version 9.4, SAS Institute).

## Results

### Patients

**Figure 1.**
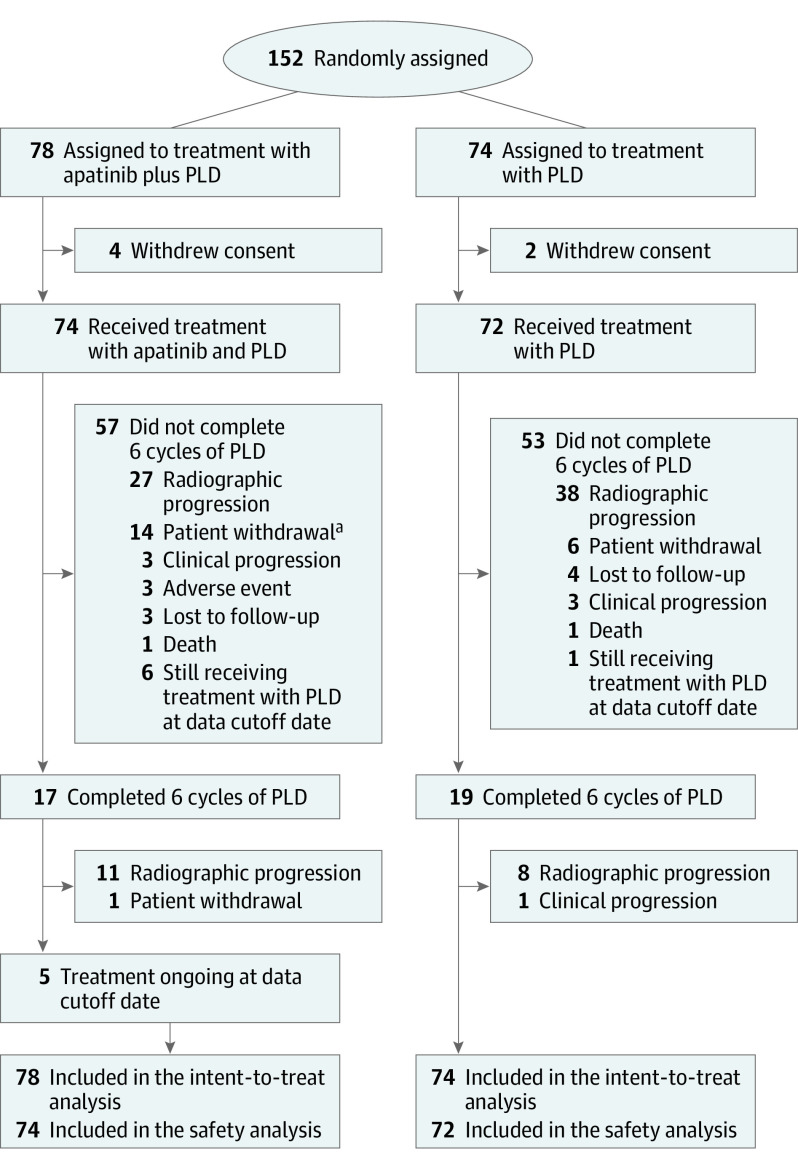
Trial Profile ^a^One patient discontinued treatment with pegylated liposomal doxorubicin (PLD) but was still receiving treatment with apatinib at the data cutoff date.

Between March 22, 2018, and November 16, 2020, 152 patients were enrolled and randomly assigned to receive treatment with apatinib plus PLD (78 [51.3%]) or PLD alone (74 [48.7%]; Figure 1). All patients assigned were included in the intent-to-treat population. Four patients in the apatinib plus PLD group and 2 in the PLD group withdrew consent before receiving the assigned treatment and were excluded from safety analyses. Baseline characteristics were well balanced between the 2 treatment groups (Table 1). At the time of data cutoff on January 28, 2021, the median follow-up duration was 8.7 months (IQR, 4.7-14.1 months).

**Table 1.** Patient Characteristics

Characteristic	No. (%)	
	Apatinib plus PLD group (n = 78)	PLD group (n = 74)
Age, median (range), y	54 (22-76)	56 (33-72)
ECOG performance status		
0	17 (21.8)	24 (32.4)
1	58 (74.4)	46 (62.2)
2	1 (1.3)	0
Missing	2 (2.6)	4 (5.4)
Primary tumor site		
Ovary	76 (97.4)	73 (98.6)
Fallopian tube	2 (2.6)	1 (1.4)
Histology at diagnosis		
Serous/adenocarcinoma	78 (100)	70 (94.6)
High grade	71 (91.0)	68 (97.1)
Low grade	3 (3.8)	2 (2.9)
Unknown	2 (2.6)	0
Adenocarcinoma	2 (2.6)	0
Endometrioid	0	2 (2.7)
Clear cell	0	2 (2.7)
FIGO stage		
I	3 (3.8)	5 (6.8)
II	3 (3.8)	3 (4.1)
III	54 (69.2)	55 (74.3)
IV	11 (14.1)	10 (13.5)
Unknown	7 (9.0)	1 (1.4)
Previous treatment lines		
1	44 (56.4)	38 (51.4)
2	18 (23.1)	26 (35.1)
≥3	16 (21.5)	10 (13.5)
Platinum-free interval, mo		
≤3	34 (43.6)	29 (39.2)
3-6	44 (56.4)	45 (60.8)
Previous chemotherapy		
Paclitaxel	78 (100)	74 (100)
Platinum	78 (100)	74 (100)
Gemcitabine	2 (2.6)	2 (2.7)
Ifosfamide	2 (2.6)	1 (1.4)
Other^a^	2 (2.6)	2 (2.7)
Previous antiangiogenic therapy		
Yes	3 (3.8)	0
No	75 (96.2)	74 (100)
Previous PARP inhibitor therapy		
Yes	5 (6.4)	2 (2.7)
No	73 (93.6)	72 (97.3)
Ascites		
Yes	30 (38.5)	28 (37.8)
No	48 (61.5)	46 (62.2)
Previous platinum-sensitive relapse		
Yes	29 (37.2)	31 (41.9)
No	49 (62.8)	43 (58.1)
Platinum refractory		
Yes	15 (19.2)	14 (18.9)
No	63 (80.8)	60 (81.1)
First platinum-resistant relapse		
Yes	61 (78.2)	56 (75.7)
No	17 (21.8)	18 (24.3)
Measurable disease	57 (73.1)	56 (75.7)

Abbreviations: ECOG, Eastern Cooperative Oncology Group; FIGO, International Federation of Gynecology and Obstetrics; PARP, polyadenosine diphosphate-ribose polymerase; PLD, pegylated liposomal doxorubicin.

^a^ One case each of fluorouracil and etoposide in the apatinib plus PLD group, as well as doxorubicin and irinotecan in the PLD group.

### Efficacy

**Figure 2.**
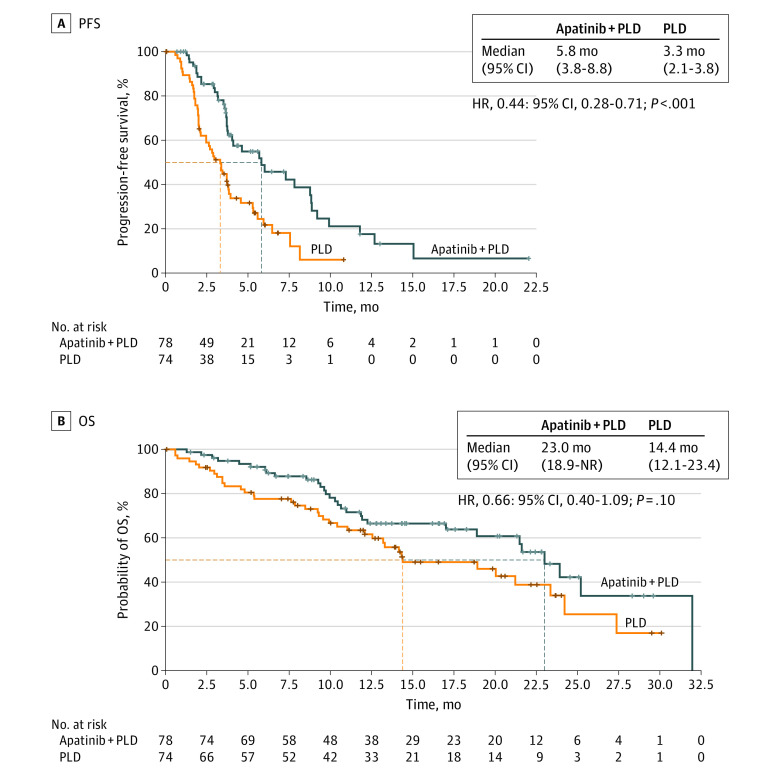
Progression-Free Survival (PFS) and Overall Survival (OS) Kaplan-Meier analysis of PFS (A) and OS (B) in the intent-to-treat population. Stratification factors included platinum-free interval (≤3 months vs 3-6 months) and prior platinum-sensitive relapse (yes vs no). HR indicates hazard ratio; NR, not reached; PLD, pegylated liposomal doxorubicin.

Thirty-seven of 78 patients (47.4%) in the apatinib plus PLD group and 50 of 74 patients (67.6%) in the PLD group experienced a progression event or death, and the median PFS was significantly improved in the apatinib plus PLD group compared with the PLD group (5.8 months [95% CI, 3.8-8.8] vs 3.3 months [95% CI, 2.1-3.8]; HR, 0.44; 95% CI, 0.28-0.71; *P* < .001; Figure 2A). Post hoc subgroup analyses showed that the PFS benefit of adding treatment with apatinib was generally consistent across subgroups, including in subgroups of patients without previous platinum-sensitive relapse (HR, 0.35; 95% CI, 0.19-0.62), patients with a PFI of 3 months or less (HR, 0.20; 95% CI, 0.09-0.44), and platinum-refractory patients (HR, 0.16; 95% CI, 0.04-0.61; eFigure in Supplement 3). The OS was not yet mature, with 56 deaths at the data cutoff date.

A post hoc analysis of updated OS was conducted on August 6, 2021. Thirty of 78 patients (38.5%) in the apatinib plus PLD group and 38 of 74 (51.4%) in the PLD group died. The median OS was 23.0 months (95% CI, 18.9 to not reached) in the apatinib plus PLD group vs 14.4 months (95% CI, 12.1-23.4) in the PLD group, with the HR for death being 0.66 (95% CI, 0.40-1.09; *P* = .10; Figure 2B).

**Figure 3.**
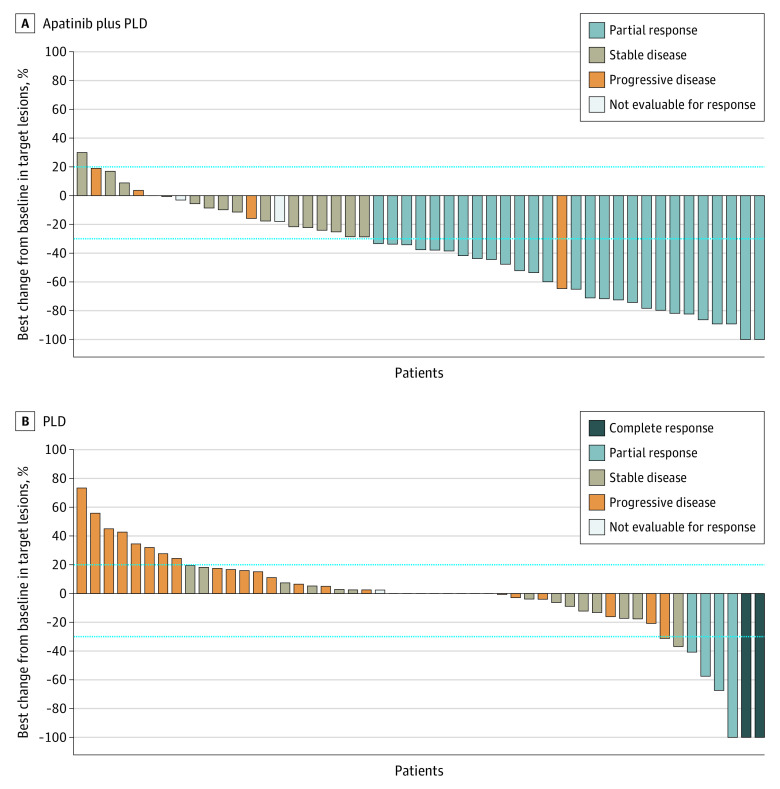
Best Percentage of Change in Target Lesions PLD indicates pegylated liposomal doxorubicin. The horizontal dashed lines represent the criteria of progrssive disease or partial response according to Response Evaluation Criteria in Solid Tumours, version 1.1.

According to RECIST, version 1.1, criteria, the objective response was assessable in 65 of 78 patients (83.3%) in the apatinib plus PLD group and 64 of 74 patients (86.5%) in the PLD group. An unconfirmed objective response was observed in 28 of 65 patients (43.1%; 95% CI, 30.8-56.0) in the apatinib plus PLD group and seven of 64 patients (10.9%; 95% CI, 4.5%-21.2%) in the PLD group (rate difference, 31.6%; 95% CI, 17.2%-46.1%; Table 2). The DCR was 81.5% (95% CI, 70.0%-90.1%) in the apatinib plus PLD group and 53.1% (95% CI, 40.2%-65.7%) in the PLD group (rate difference, 28.9%; 95% CI, 13.5%-44.4%; Table 2). The median time to response was 2.1 months (95% CI, 1.8-2.3) in the apatinib plus PLD group and 2.1 months (95% CI, 1.8-3.5) in the PLD group. Among patients who possessed measurable lesions and underwent at least 1 postbaseline imaging assessment, 43 of 49 patients (87.8%) in the apatinib plus PLD group and 20 of 51 patients (39.2%) in the PLD group achieved target lesion reduction (Figure 3).

**Table 2.** Treatment Responses

Response^a^	No. (%)	
	Apatinib plus PLD group (n = 65)	PLD group (n = 64)
Best overall response		
Complete response^b^	1 (1.5)	3 (4.7)
Partial response^b^	27 (41.5)	4 (6.3)
Stable disease	25 (38.5)	27 (42.2)
Progressive disease	10 (15.4)	28 (43.8)
Not evaluable^c^	2 (3.1)	2 (3.1)
Objective response	28 (43.1)	7 (10.9)
(95% CI)	(30.8-56.0)	(4.5-21.2)
Disease control	53 (81.5)	34 (53.1)
(95% CI)	(70.0-90.1)	(40.2-65.7)

Abbreviation: PLD, pegylated liposomal doxorubicin; RECIST, Response Evaluation Criteria in Solid Tumours.

^a^ Responses were evaluated by the investigators in patients who underwent at least 1 efficacy assessment after randomization according to RECIST, version 1.1.

^b^ Unconfirmed response.

^c^ Not evaluable was defined as stable disease not reaching at least 6 weeks according to RECIST, version 1.1, at the time of data cutoff.

### Safety

The median relative dose intensity for PLD was 96% (range, 33%-124%) in the PLD group and 98% (range, 34%-137%) in the apatinib plus PLD group. The median relative dose intensity for apatinib was 100% (range, 28%-101%). Apatinib dose reduction was reported in 9 of 74 patients (12.2%), and PLD dose reduction was reported in 5 patients (6.8%) in the apatinib plus PLD group vs 1 of 72 patients (1.4%) in the PLD group. At least 1 dose interruption of apatinib occurred in 22 patients (29.7%), and at least 1 dose interruption of PLD occurred in 1 patient (1.4%) in the PLD group and none in the apatinib plus PLD group. Most dose reductions and interruptions followed AEs. Three patients (3.8%) in the apatinib plus PLD group and none in the PLD group discontinued treatment owing to AEs.

In total, 69 of 74 patients (93.2%) in the apatinib plus PLD group and 61 of 72 patients (84.7%) in the PLD group experienced at least 1 treatment-emergent AE (TEAEs; eTable 2 in Supplement 3). The most common TEAEs of any grade in both groups were decreased white blood cell count (45 [60.8%] in the apatinib plus PLD group vs 36 [50.0%] in the PLD group) and decreased neutrophil counts (44 [59.5%] vs 27 [37.5%]; eTable 2 in Supplement 3). An increased incidence of any grade hand-foot syndrome (19 [25.7%] in the apatinib plus PLD group vs 2 [2.8%] in the PLD group), hypertension (13 [17.6%] vs 1 [1.4%]), and proteinuria (11 [14.9%] vs 2 [2.8%]) was observed with treatment with apatinib. Grade 3 or higher TEAEs occurred in 32 of 74 patients (43.2%) in the apatinib plus PLD group and 14 of 72 patients (19.4%) in the PLD group, with decreased neutrophil counts (11 [14.9%] vs 6 [8.3%]), hypertension (6 [8.1%] vs none) and decreased white blood cell counts (5 [6.8%] vs 3 [4.2%]) being the most frequent AEs. Two patients (2.7%) in the apatinib plus PLD group experienced grade 3 proteinuria compared with none in the PLD group. Two patients in the apatinib plus PLD group experienced grade 2 fistulas. Serious AEs were reported in nine of the 74 patients (12.2%) in the apatinib plus PLD group, with the most common being incomplete bowel obstruction (2 [2.7%]) and intestinal fistula (2 [2.7%]), and in 3 of 72 patients (4.2%) in the PLD group, including 1 instance each of infection, vomiting/nausea, and acute pancreatitis. No patient died of AEs.

## Discussion

To our knowledge, APPROVE is the first randomized clinical trial to compare treatment with apatinib plus single-agent chemotherapy with single-agent chemotherapy alone in PROC. The APPROVE trial met the primary end point, showing that the addition of treatment with apatinib to PLD for PROC resulted in a statistically significant prolongation in PFS. The risk of disease progression was reduced by 56%. This positive finding was consistent with previously published studies of other antiangiogenic agents combined with nonplatinum monochemotherapy (AURELIA^4,12^ and MITO 11^6^). Additionally, the apatinib plus PLD regimen was well tolerated without new safety concerns.

The PFS benefits were generally consistent across all subgroups. The HRs indicated a more substantial benefit in patients with previous nonplatinum-sensitive relapse, a PFI of 3 months or less, platinum-refractory disease, or multiple platinum-resistant relapses, with reduced progression risks of 65%, 80%, 84%, and 63%, respectively. These subsets of patients have a worse prognosis, with very limited systemic therapeutic options, and are often excluded, or only a small proportion are included in other randomized trials (eg, AURELIA^4^ and MITO 11^6^).

The AURELIA trial reported that treatment with bevacizumab plus PLD did not yield a significant improvement in OS compared with PLD alone (13.7 months vs 14.1 months; HR, 0.91; 95% CI, 0.62-1.36).^12^ The benefit of adding bevacizumab might have been concealed because of the crossover to bevacizumab that was allowed after disease progression. In addition, the comparison of treatment with bevacizumab plus PLD with PLD was a subset analysis with no power provided to it. For the APPROVE trial, the median OS of 23.0 months with treatment with apatinib and PLD was numerically longer than the results reported in the AURELIA trials. The risk of death was reduced by 34% (HR, 0.66; 95% CI, 0.40-1.09), although no statistical difference was observed. Similar to AURELIA, APPROVE also used OS as a secondary end point, with no statistical assumptions made. Additionally, only 38% of deaths occurred at the time of this analysis, and the findings might change with prolonged follow-up.

In the APPROVE trial, treatment with PLD was discontinued after 6 cycles, whereas chemotherapy was administered until disease progression in the AURELIA trial.^4^ However, the AURELIA trial showed that the incidence of cumulative chemotherapy-related grade 2 or greater AEs was increased with continued chemotherapy after the completion of 6 cycles. In TRIAS trial, 6 treatment cycles of topotecan were chosen to avoid cumulative toxic effects.^13^ Based on these, the duration of PLD was limited in the APPROVE trial from the perspective of avoiding cumulative toxic effects. Despite limiting treatment with PLD to 6 cycles, most patients in the PLD group discontinued treatment before completing 6 cycles owing to disease progression.

In this trial, AEs were mostly consistent with the previously published safety profile of treatment with apatinib plus chemotherapy in OC and other solid tumors,^9,14,15^ with no new safety concerns identified. In this trial, most TEAEs were grade 1 or 2 and relieved by symptomatic treatment. Hypertension, hand-foot syndrome, and proteinuria were the most common grade 3 or higher nonhematological AEs with treatment with apatinib, and their occurrence has been reported to be associated with better antitumor efficacy in patients with advanced solid tumors who are receiving VEGF TKIs.^16,17,18^ However, compared with those in previous studies,^9,15^ the lower incidences of any-grade hypertension (17.6%), hand-foot syndrome (25.7%), and proteinuria (14.9%) in the present trial might be attributed to the low-dose administration of apatinib. Additionally, hemorrhage events, frequent adverse effects of all antiangiogenic agents, were also uncommon in this trial because of the exclusion of patients with a bleeding tendency and the low-dose administration of apatinib.

### Limitations

This trial had several limitations. First, the OS events in the combination group were immature. Extended follow-up is warranted. Second, APPROVE trial enrollment was limited to Chinese patients. The effect of treatment with apatinib plus chemotherapy in populations from other countries should be determined. Third, we failed to collect data related to patient quality of life. It was unclear whether the improvement in PFS with the combination of apatinib and PLD affected the quality of life of patients. Fourth, only 3 patients who received previous antiangiogenic therapy were included, and it might be of interest to provide an effective strategy for subsequent treatment in this setting.

## Conclusions

In the APPROVE randomized clinical trial, compared with treatment with PLD alone, apatinib combined with PLD led to significant improvements in PFS and ORR, with manageable toxic effects. Although the outcomes of apatinib plus PLD were comparable with those receiving treatment with bevacizumab plus PLD, bevacizumab is administered by intravenous infusion, whereas apatinib is received orally, which allows for timely dose adjustments and is much more convenient for patients. In addition, as a small molecular TKI, the mechanism of apatinib is not identical to that of bevacizumab. Thus, apatinib plus PLD could be considered an alternative treatment option for PROC.

## Notes

Supplement 1. Trial protocol

Supplement 2. Statistical analysis plan

Supplement 3. eTable 1. List of 11 participating sites eTable 2. Treatment-emergent adverse events eFigure. Subgroup analyses of progression-free survival

Supplement 4. Data sharing statement
