# Supplementary material for: Effect of Apatinib Plus Pegylated Liposomal Doxorubicin vs Pegylated Liposomal Doxorubicin Alone on Platinum-Resistant Recurrent Ovarian Cancer: The APPROVE Randomized Clinical Trial
Source: JAMA Oncol. 2022 Jun 30;8(8):1169–76. doi: 10.1001/jamaoncol.2022.2253 (PMC9247861; doi:10.1001/jamaoncol.2022.2253)
Supplement: Supplement 1. — Trial protocol [file jamaoncol-e222253-s001.pdf]

Supplement

The Supplement contains the following items:

1. Original protocol

2. Final protocol

3. Amendment history

Apatinib Combined with Standard Chemotherapy for Platinum-Resistant Recurrent Ovarian Cancer: The APPROVE Study

Study protocol

Version 1.0

Version date: July 19, 2016

**Project:**	**Optimization of treatment regimens and clinical pathways for ovarian cancer**
**Programme:**	**Research on the prevention and control of major chronic non-communicable diseases**
**Lead Site:**	**Cancer Hospital, Chinese Academy of Medical Sciences**
**Research Site:**	**Cancer Hospital, Chinese Academy of Medical Sciences**
**Principal investigator:**	**Lingying Wu**
**Implementation period:**	**September 2016-December 2020**

Collaborating institutions and contacts

Serial number	Institution name	Responsible person/Contact
1	Cancer Hospital, Chinese Academy of Medical Sciences	Lingying Wu/Ning Li
2	Peking Union Medical College Hospital	Lingya Pan/Yu Gu
3	West China Second University Hospital of Sichuan University	Rutie Yin/Qingli Li/Xinxin Jiang
4	Chongqing University Cancer Hospital	Dongling Zou
5	Peking University Cancer Hospital	Hong Zheng/Qian Li
6	Fudan University Shanghai Cancer Center	Xiaojun Chen/Wei Zhang
7	Hunan Cancer Hospital	Jie Tang/Ying Wang
8	The Second Affiliated Hospital of Dalian Medical University	Kui Jiang
9	The First Hospital of Jilin University	Ziling Liu/Zhi Li
10	Xiangya Hospital of Central South University	Lanqin Cao
11	Liaoning Cancer Hospital & Institute	Jingru Zhang
12	Beijing Obstetrics and Gynecology Hospital, Capital Medical University	Jinwei Miao/Di Wu
13	Guangxi Medical University Affiliated Tumor Hospital	Li Li/Bingbing Zhao
14	Hubei Cancer Hospital	Yi Huang
15	Yunnan Cancer Hospital	Hongying Yang/Yi Zhang
16	Tianjin Medical University Cancer Institute & Hospital	Ke Wang/Li Zhang
17	Shandong Cancer Hospital	Qingshui Li

Table of contents

Synopsis.....5
1. **Background**.....11
    1.1 Progress in antiangiogenic therapy for recurrent platinum-resistant ovarian cancer.....11
    1.2 Drug name.....14
    1.3 Results of preclinical studies of apatinib.....14
    1.4 Results of the phase I clinical trial of apatinib.....17
2. **Study purpose**.....22
    2.1 Primary outcome measures.....22
    2.2 Secondary outcome measures.....22
3. **Study design**.....23
4. **Subjects**.....23
    4.1 Inclusion criteria.....23
    4.2 Exclusion criteria.....24
    4.3 Criteria for termination or withdrawal.....26
    4.4 Relevant post-withdrawal regulations for patients in the monochemotherapy group.....26
    4.5 Early termination of the research project.....27
5. Study schedule and evaluation.....27
    5.1 Screening examination and baseline evaluation (will be completed within 21 days before the start of treatment).....27
    5.2 Evaluation of the treatment period.....28
    5.3 End-of-study evaluation.....29
    5.4 Follow-up.....30
    5.5 Evaluation of early exit and withdrawal.....30
    5.6 Study duration.....30
    5.7 Study schedule.....30
6. **Investigational drug**.....34
    6.1 Treatment regimens.....34
    6.2 Dose adjustment.....34
    6.3 Concomitant therapy.....43
    6.4 Drug policy.....44
7. Study endpoint evaluation.....44
    7.1 Efficacy evaluation.....44
    7.2 Safety evaluation.....44
8. Data management.....49
    8.1 Data entry and modification.....49
    8.2 Database security.....50
    8.3 Data storage.....50
9. Statistical analysis.....50
    9.1 Sample size determination.....50
    9.2 Statistical analysis of datasets.....50
    9.3 Statistical analysis methods.....51
10. Ethical norms and quality control.....51
    10.1 Informed consent.....52

10.2 Ethical norms and policies and regulations 52
10.3 Quality Assurance 52
11. References 53
12. The requirements for biological sample collection from the enrolled patients 53
12.1 Tissue sections 53
12.2 Blood collection requirements 54
12.3 Technical roadmap 54
Appendix 54
Appendix 1. ECOG performance status scoring criteria 55
Appendix 2. EORTC QLQ-C30 V3.0 56
Appendix 3. Response Evaluation Criteria in Solid Tumours (RECIST 1.1) criteria 59
Appendix 4. 2014 FIGO staging for ovarian cancer, fallopian tube cancer, and peritoneal cancer 74
Appendix 5. New York Heart Association (NYHA) classification 75

Synopsis

Title	Apatinib Combined with Standard Chemotherapy for Platinum-Resistant Recurrent Ovarian Cancer: The APPROVE Study
Sponsor	National Cancer Center/National Clinical Research Center for Cancer/Cancer Hospital, Chinese Academy of Medical Sciences and Peking Union Medical College
Principal investigator	Lingying Wu
Study population	Patients with platinum-resistant recurrent ovarian cancer
Study design	A randomized, parallel-controlled, multicentre, phase II study
Study objectives	Primary endpoint: progression-free survival (PFS)<br>Secondary endpoints: overall survival (OS), objective response rate (ORR), disease control rate (DCR), and safety
Treatment regimens	Drugs: doxorubicin hydrochloride liposomes, apatinib mesylate tablets<br>Treatment group (group A):<br>Doxorubicin hydrochloride liposomes: 40 mg/m^2 Q4W + apatinib (500 mg po qd);<br>Control group (group B):<br>Doxorubicin hydrochloride liposomes: 40 mg/m^2 Q4W;<br>1. The enrolled patients are required to have received at least 4 cycles of platinum-based first- or second-line chemotherapy and have experienced disease progression within 6 months after platinum-based chemotherapy.<br>2. Patients in groups A and B who achieve effective treatment will not receive more than 6 cycles of chemotherapy. Afterwards, group A will receive apatinib orally for maintenance therapy until disease progression or intolerance, and group B will be followed up. Patients who progress during treatment will stop treatment and withdraw from the study.
Sample collection requirements	1. Paraffin sections of tumours from the initial surgery will be collected from the enrolled patients, and paraffin sections of tumours from patients

	who underwent the initial surgery in other hospitals will be obtained from those hospitals.<br>Sectioning requirements: A total of 10 5-μm-thick or 5 10-μm-thick unstained tissue sections, of which nucleated cells compose more than 80%, with the local tumour cell content exceeding 70%, will be stored at room temperature (paraffin sections of tumours from patients enrolled in other sites will be sent to the Cancer Hospital of the Chinese Academy of Medical Sciences).<br>2. Peripheral blood collection time: Peripheral blood samples (10 mL) will be collected a total of 4 times, namely, before the first, third, and fifth cycles of chemotherapy and one month after the end of the sixth cycle of chemotherapy. If a patient withdraws from the study due to disease progression, another blood sample (10 mL) will be collected. For patients receiving maintenance treatment in the group A, blood samples (10 mL) will be collected during follow-up examinations every 2 months. All blood samples will be collected using 10-mL cfDNA sample storage tubes. After the blood samples are collected, the tubes will be turned upside down 10 times and stored at room temperature (blood samples from the patients enrolled in other sites shall be sent to the Cancer Hospital of the Chinese Academy of Medical Sciences within 5 days).
Planned total number of patients	126 patients (1:1)
Inclusion criteria	1. Previous pathological diagnosis of ovarian cancer, fallopian tube cancer, or primary peritoneal cancer, with available paraffin sections from a previous surgery;<br>2. Platinum-resistant relapse (relapse within 6 months after the last chemotherapy)<br>3. Combined with malignant pleural effusion or ascites or with clinically evaluable recurrent lesions;

	4. Eastern Cooperative Oncology Group (ECOG) performance status of 0 or 1;<br>5. Expected survival ≥ 4 months;<br>6. No history of anti-vascular targeted therapy;<br>7. At least 1 measurable lesion as the target lesion based on the Response Evaluation Criteria in Solid Tumours (RECIST 1.1) criteria; if the target lesion is a lymph node, the diameter must be greater than 1.5 cm, and it must be unsuitable for surgical treatment; and the target lesion must be free of radiotherapy or relapsed within the radiotherapy field;<br>8. The baseline blood routine meets the following criteria:<br>a) Neutrophil count ≥ 1.5×10^9/L,<br>b) Platelet count ≥ 100×10^9/L, and<br>c) Haemoglobin ≥ 9 g/dL (allowing blood transfusion to achieve or maintain this level);<br>9. Liver function meets the following criteria:<br>a) Total bilirubin < 1.5 times the upper limit of normal (ULN);<br>b) Aspartate aminotransferase (AST) and alanine aminotransferase (ALT) < 2.5 × ULN in patients without hepatic metastasis and < 5 × ULN in patients with hepatic metastasis;<br>10. Serum creatinine ≤ 1.25 × ULN or the calculated creatinine clearance ≥ 50 mL/min;
Exclusion criteria	1. Having received more than 2 chemotherapy regimens;<br>2. Refractory patients progressing during previous treatment;<br>3. Current or recent (within 30 days prior to enrolment) usage of another investigational drug or participating in another clinical study;<br>4. Other malignancies occurred within 5 years (except for fully treated cervical carcinoma in situ or skin squamous cell carcinoma or controlled basal cell carcinoma of the skin);<br>5. Hypertension that cannot be resolved, i.e. out of the normal range, by

	antihypertensive drug treatment (systolic blood pressure ≥ 140 mmHg or diastolic blood pressure ≥ 90 mmHg);<br>6. Grade II or higher myocardial ischaemia or myocardial infarction and poorly controlled arrhythmias (including heart rate-corrected QT (QTc) ≥ 450 ms for males and QTc ≥ 470 ms for females);<br>7. Previous or current cardiac insufficiency of grade II or above based on the New York Heart Association (NYHA) criteria, or the left ventricular ejection fraction (LVEF) lower than 50% or the lower limit of normal as evidenced by echocardiography;<br>8. Coagulation disorders [international normalized ratio (INR) >1.5, prothrombin time (PT) > ULN + 4 s, or activated partial thromboplastin time (APTT) >1.5 × ULN], bleeding tendency, or current thrombolysis or anticoagulation therapy;<br>9. Clinically significant bleeding symptoms or clear bleeding tendency, such as gastrointestinal bleeding, haemorrhagic gastric ulcer, baseline faecal occult blood of ++ or above, and vasculitis, within 3 months before randomization;<br>10. Major surgery or severe traumatic injury, fracture or ulcer within 4 weeks before randomization;<br>11. Factors that significantly affect the absorption of oral drugs, such as the inability to swallow, chronic diarrhoea, and intestinal obstruction;<br>12. Urinary protein ≥ ++ indicated by urinalysis or 24-h urinary protein ≥ 1.0 g;<br>13. Other conditions that may affect the clinical study or the interpretation of the study results according to the researcher's judgement;<br>14. Allergy to doxorubicin and/or related substances or the presence of idiosyncratic reactions;<br>15. An expected cumulative dose of doxorubicin (including previous anthracyclines, if any) reaching or exceeding 550 mg/m^2 after 4 cycles of

	doxorubicin hydrochloride injection;<br>16. Uncontrollable arrhythmia or other ECG abnormalities indicative of a research risk, as determined by the principal investigator;<br>17. Doxorubicin liposome treatment in the past 6 months;<br>18. History of local radiation therapy.
Criteria for termination or withdrawal	1. Patients can freely withdraw from the study at any time without any reason;<br>2. Concurrent radiotherapy on target lesions for efficacy observation;<br>3. Violation of the protocol that affects the evaluation of the results;<br>4. The investigators terminate the study due to safety profile among the enrolled patients [such as intolerable adverse events (AEs), pregnancy during treatment, or other events that affect the safety of the subjects];<br>5. When the inclusion/exclusion criteria are violated or the subject lacks compliance with or has to use unauthorized drugs, the investigator and/or the sponsor can decide to discontinue the treatment;<br>6. Progressive disease (PD);<br>7. Medical or ethical reasons that affect continuation in the study;<br>8. Poor quality data and incomplete and inaccurate information;<br>9. Patients receive other antitumour drugs during the clinical study period;<br>10. The sponsor terminates the study.
Efficacy evaluation	The contrast-enhanced thoraco-abdominal pelvic CT will be performed every 2 cycles (i.e., 8 weeks). Efficacy will be evaluated based on the RECIST 1.1 criteria or the Gynecologic Cancer Intergroup (GCIG) CA-125 criteria.
Safety evaluation	AEs will be graded and recorded in accordance with the National Cancer Institute's Common Terminology Criteria for Adverse Events (NCI-CTCAE) version 4.0. During the treatment period, a full physical examination, vital signs, laboratory safety evaluation, and AEs will be recorded at each visit.

Statistical analysis	Primary outcome measures: PFS will be analysed using the Kaplan-Meier method and the K-M plots will be provided. The median PFS and the corresponding 95% confidence interval will also be reported. The difference in survival between the two groups will be compared by using the log-rank test. Hazard ratio will be estimated by using the Cox proportional hazards model.<br>Secondary outcome measures: ORR and DCR will be described using a frequency table and two-sided 95% confidence intervals will be calculated by using Clopper-Pearson method. OS will be analysed using the Kaplan-Meier method and the K-M plots will be provided. The median OS and the corresponding 95% confidence interval will be reported. Hazard ratio will be estimated by using the Cox proportional hazards model. Safety: The number of occurrences and the incidence of safety events will be provided in a tabular format.
Study plan	Enrolment began in September 2016 and is expected to last for 2 years. The end of the study is defined as the completion of at least 2 efficacy evaluations for the last subject under continuous treatment or the occurrence of disease progression or intolerable toxicity.

1. Background

1.1 Progress in antiangiogenic therapy for recurrent platinum-resistant ovarian cancer

Ovarian cancer is a gynaecological malignancy with a poor prognosis. Although most ovarian cancer patients can achieve a clinical response after surgery and first-line chemotherapy, the vast majority of patients experience the painful process of recurrence, chemotherapy, recurrence, and retreatment and eventually develop drug resistance, leading to treatment failure. The main purpose of recurrent ovarian cancer treatment is to improve the quality of life and prolong the survival of patients. In the past 20 years, developments in surgery, chemotherapy and radiotherapy have played a limited role in improving the prognosis of patients with recurrent ovarian cancer. Targeted therapy is a medical treatment method that has emerged in recent years. Its emergence and development depend on the continuous exploration of tumourigenesis and development mechanisms via tumour molecular biology. Targeted therapy uses the characteristic changes in tumour cells at the molecular level as targets and exerts antitumour effects by interfering with molecules that play important roles in the development and progression of tumours. In the past 10 years, myriad clinical studies have evaluated the efficacy and side effects of targeted drugs for recurrent ovarian cancer and have achieved notable results.

Among the various types of targeted drugs, antiangiogenic drugs have been used the most to treat recurrent ovarian cancer, and encouraging results have been achieved. Angiogenesis is an indispensable step for the growth and metabolism of normal cells and tumour cells. However, the angiogenesis process and the composition and distribution of blood vessels in tumour tissues are not completely the same as those in normal tissues. Under the action of some angiogenic factors, tumour tissues are prone to vasodilation, local tissue pressure increases, and neovascular leakage, resulting in compromised oxygen and nutrient supplies. This compromise may be one of the reasons for the necrotic tendency in most solid tumours and may inhibit drug delivery to tumour tissues, thus affecting efficacy. In addition, endothelial cell proliferation, migration, and

invasion during tumour metastasis are closely related to a variety of angiogenic factors. Therefore, drugs targeting angiogenesis should have good prospects for antitumour therapy.

There are many factors involved in angiogenesis, of which vascular endothelial growth factor (VEGF) and its receptor (VEGFR) and angiogenin have become the main targets of existing drugs. Numerous studies have shown that VEGF and its receptors play important roles in both physiological and pathological angiogenesis. VEGF also plays an important role in the development of ovarian cancer and the formation of malignant ascites. VEGF has been reported to be overexpressed in 53% to 97% of ovarian cancers.[1] *In vitro* studies have shown that anti-VEGF drugs can inhibit angiogenesis in tumour tissues, reduce the tumour feeding, and slow tumour growth. In addition, anti-VEGF drugs can normalize the vascular structure of tumour tissues, promote the effective reach of chemotherapeutic drugs to tumour tissues, and have a synergistic effect with chemotherapy.

Bevacizumab is a recombinant, humanized monoclonal anti-VEGF antibody. It inhibits VEGF, suppressing angiogenesis and thereby resulting in 3 effects: (1) tumour vascular degeneration (cutting off the nutrient supply to tumour cells), (2) inhibition of angiogenesis and revascularization (continuous inhibition of residual and new tumour cells), and (3) normalization of surviving vessels (reducing plasma leakage and increasing drug delivery under interstitial pressure). The development of the theory of vascular dependence of tumours has formed a therapeutic strategy that emphasizes both anti-angiogenesis and anti-cell proliferation approaches.

In 2014, a randomized, open-label, phase III clinical trial (AURELIA trial) reported by Andres Poveda et al. from Spain showed that bevacizumab combined with monochemotherapy significantly improved the progression-free survival (PFS) of and objective response rate (ORR) for patients with recurrent platinum-resistant ovarian cancer (PROC).[2] In the AURELIA trial, patients were assigned to a control group (chemotherapy alone) or an experimental group (chemotherapy plus bevacizumab), and the chemotherapy regimen [paclitaxel, pegylated liposomal doxorubicin (PLD) or topotecan] for each patient was chosen by the investigators. The results indicated that

the PFS of the patients in the experimental group improved significantly. The hazard ratios (HRs) of PFS were 0.46 for patients who received paclitaxel (median, 10.4 months vs 3.9 months in the experimental and control groups, respectively), 0.57 for patients who received PLD (median, 5.4 months vs 3.5 months), and 0.32 in patients who received topotecan (median, 5.8 months vs 2.1 months). Different chemotherapy regimens combined with bevacizumab all demonstrated PFS benefits.

Based on the Response Evaluation Criteria in Solid Tumours (RECIST 1.1) criteria, patients who received bevacizumab plus paclitaxel (53.3% vs 30.2%) and patients who received bevacizumab plus topotecan (17.0% % vs 0) had a significantly higher ORR than did patients who received monochemotherapy, while patients who received PLD had a similar ORR to that for patients who received monochemotherapy (13.7% vs. 7.8%).

Patients' self-reported outcomes were evaluated using the European Organisation for Research and Treatment of Cancer (EORTC) Quality of Life Questionnaire-Ovarian Cancer Module (QLQ-OV28). The abdominal/gastrointestinal symptoms improved by ≥ 15% in the 8th and 9th weeks. In each cohort, the proportions of patients whose abdominal/gastrointestinal symptoms improved were higher among those who received bevacizumab combined with chemotherapy (25.0% of patients who received bevacizumab plus paclitaxel, 20.0% of patients who received bevacizumab plus topotecan, and 21.1% of patients who received bevacizumab plus PLD) than among those who received chemotherapy alone (13.0% of patients who received paclitaxel alone, 8.8% of patients who received topotecan alone, and 6.8% of patients who received PLD alone).

Based on the results of the phase III AURELIA trial, the Food and Drug Administration (FDA) approved bevacizumab for the treatment of recurrent PROC in 2014. Although bevacizumab has not been approved in China, it is a clinical treatment option for recurrent PROC. However, because of the high price of bevacizumab, many patients cannot afford it, limiting its clinical application to a certain extent. In summary, antiangiogenic therapy has broad application prospects in cancer treatment. However, more clinical studies are still needed to confirm the efficacy of antiangiogenic targeted

drugs in ovarian cancer.

1.2 Drug name

The chemical name of apatinib mesylate (abbreviated as apatinib) is N-[4-(1-cyanocyclopentyl)phenyl]-2-[(4-pyridylmethyl)amino]-3-pyridinecarboxamide methanesulfonate. Its molecular formula is $C_{25}H_{27}N_{5}0_{3}S$, with a molecular mass of 493.58 (methanesulfonate).

1.3 Results of preclinical studies of apatinib

1.3.1 Pharmacokinetics study

The results of the pharmacokinetics study in beagle dogs indicated that after a single dose of 5 mg/kg apatinib administered intravenously, the highest plasma concentrations (C_{max}) of apatinib were 6058 ng/kg in male beagle dogs and 3523 ng/mL in female beagle dogs. The area under the plasma concentration-time curve ($AUC_{0\rightarrow24\ h}$) was 12599 ng/mL·h (male) and 9106 ng/mL·h (female). The elimination half-lives ($T_{1/2}$) were 2.15 h (male) and 3.22 h (female), and the elimination rate constants (Kel) were 0.328 h^{-1} (male) and 0.216 h^{-1} (female). The mean retention times (MRTs) were 3.08 h (male) and 3.81 h (female). The total plasma drug clearance rates (CL) were 0.385 L/h/kg (male) and 0.515 L/h/kg (female). The apparent distribution volumes (Vd) were 1.19 L/kg (male) and 1.94 L/kg (female).

1.3.2 Preclinical pharmacodynamics study

In vitro: The sulforhodamine B (SRB) or 3-(4,5-dimethylthiazol-2-yl)-2,5-diphenyl tetrazolium bromide (MTT) assay was used to evaluate the growth inhibitory effect of apatinib on a variety of *in vitro* cultured cancer cells, such as colon cancer cells, lung cancer cells, gastric cancer cells, renal carcinoma cells and leukaemia cells. The half-maximal inhibitory concentration (IC_{50}) of apatinib against the above cancer cell lines exceeds 20 μM, a concentration much higher than that needed to inhibit tyrosine kinase receptors, including VEGFR, indicating that apatinib has no cytotoxicity. *In vivo*: Apatinib has a marked antitumour effect on a variety of human cancer xenografts in

nude mice, including colon cancer, lung cancer, and gastric cancer. In addition, apatinib enhances the efficacy of conventional cytotoxic drugs including oxaliplatin, 5-fluorouracil (5-FU), docetaxel and doxorubicin, and its efficacy is markedly better than that of PTK787 and comparable to that of ZD6474 and AMG706.

Antitumour mechanism: Apatinib can effectively inhibit VEGFR2 at extremely low concentrations and can inhibit kinases, such as PDGFR, c-Kit and c-Src, at high concentrations, as shown in the table below. Apatinib is 13.7 times more effective than PTK787 in inhibiting VEGFR2 activity. Moreover, apatinib inhibits downstream signal transduction mediated by VEGFR2. Apatinib also inhibits the growth of KDR/NIH3T3 high-expressing cell lines, VEGF-induced proliferation and migration of human umbilical vein endothelial cells and lumen formation, and microvascular genesis in rat arterial rings. The *in vitro* antiangiogenic effect of apatinib is stronger or equivalent to that of the control compound PTK787.

1.3.3 Toxicological studies of apatinib

1.3.3.1 Acute toxicity tests in animals

(1) Mice: A total of 40 ICR mice were randomly divided into a 5 g/kg dose group and a solvent control group, with 20 mice in each group and a male-to-female ratio of 1:1. An immediate response was observed after a single intragastric administration of apatinib. The mice were continuously observed for 14 days, and toxicity responses and death were recorded. All mice were dissected on the 15th day, and no drug-related changes were observed in various organs. During the observation period, except for the slightly slower body weight gain of the mice in the 5 g/kg dose group, no significant clinical toxic reactions or death were observed.

(2) Rats: A total of 80 SD rats were randomly divided into 4 groups, namely, a 2 g/kg apatinib group, a 5 g/kg apatinib group, and their respective control groups, with 20 rats in each group. An immediate response was observed after a single intragastric administration of apatinib. The mice were continuously observed for 14 days, and toxicity responses and death were recorded. During the observation period, the mortality rates in the 5 g/kg apatinib group were 50% in female rats and 20% in male

rats. In the 2 g/kg apatinib group, 1 female rat died; therefore, the mortality rate for female rats was 10%, while no male rats died. The anatomy of a dead female mouse in the 5 g/kg apatinib group revealed bilateral enlargement of the adrenal glands and yellow plaques in the right kidney. Hepatic and renal function tests revealed that alanine aminotransferase (ALT), aspartate aminotransferase (AST), and blood urea nitrogen (BUN) concentrations were elevated more than 3 times baseline levels. A pathological examination revealed scattered punctate necrosis of hepatocytes, adrenal haemorrhage, thymic atrophy, and lymphocyte reduction in the germinal centre of splenic white pulp. The other rats that died and the rats that were dissected after 14 days showed no obvious abnormalities.

(3) Beagle dogs: Six beagle dogs (body weight, approximately 7 kg; male-to-female ratio, 1:1) were orally administered apatinib. The doses for the 3 female dogs were 1050, 1575, and 2363 mg/kg (50% increments), and the doses for the 3 male dogs were 1575, 2363, and 3545 mg/kg (50% increments). After the administration of apatinib, female beagle dogs showed reduced activity, weakness in the limbs, and gait instability, and the female dog that received 2363 mg/kg apatinib exhibited a 5-fold increase in BUN levels. Male dogs began to vomit 4 h after the administration of apatinib, and they had reduced activity, weakness in the limbs, and reduced food intake. The degree of the toxic reaction increased with increasing dose, but no dog died.

1.3.2 Long-term toxicity test in animals

(1) Rats: Sprague-Dawley rats received apatinib intragastrically for 13 weeks, with a 4-week recovery period, during which they were observed. The rats were divided into 4 groups, namely, 3 dose groups [5, 15 and 50 mg/(kg·d) groups] and 1 solvent control group, with 14 female rats and 14 male rats in each group. No drug-related changes were observed in any rat in the 5 mg/(kg·d) group. The ALT levels in the male rats in the 15 mg/(kg·d) group were slightly elevated during the treatment period but recovered after the drug was discontinued. All rats in the 50 mg/(kg·d) group experienced slow body weight gain, decreased food consumption, weight loss, and mild increases in the liver function indicators ALT, AST, and ALP during the treatment period; however, no drug-related changes in organs were observed during pathological examinations. In

addition, the female rats in this group exhibited incisor fractures (11/14, 78.6%) and a mild reduction in tibial bone mineral density. After drug withdrawal, except for bone changes, which partially recovered, other changes fully reverted. Histological examinations revealed that compared with solvent control group, the 15 mg/(kg·d) group mainly suffered incisor fractures, which partially healed during the 4-week recovery period, and in severe cases, the incisors completely fell out. No drug-related pathological changes were observed in the other 2 dose groups.

(2) Beagle dogs: Twenty-four beagle dogs (body weight, approximately 7 kg; male-to-female ratio, 1:1) were randomly assigned into 4 groups, namely, 3 dose groups [3, 10, and 30 mg/(kg·d) groups] and a control group, with 6 dogs in each group (3 female dogs and 3 male dogs). The dogs were administered apatinib for 13 weeks. The haematology, blood biochemistry, electrocardiogram and bone mineral density indicators in each dose group were similar to those in the control group, and all fluctuated within the normal range, suggesting that liver function and kidney function were essentially normal. During the treatment period, for dogs in the 30 mg/(kg·d) group, the skin colour of the nose and corners of the mouth became pale, and the pathology indicated that their nasal skin became thinner; no abnormalities were observed in the control group.

1.4 Results of the phase I clinical trial of apatinib

1.4.1 Phase I apatinib drug tolerance study

In accordance with the modified Fibonacci method, patients who received apatinib were assigned to 1 of 5 dose groups, namely, a 250 mg group, a 500 mg group, a 750 mg group, an 850 mg group, and a 1000 mg group. Haematological toxicity above grade 4 and non-haematological toxicity above grade 3 were regarded as dose-limiting toxicity (DLT). A total of 18 patients were evaluable for tolerance to apatinib, including 6 patients in the 850 mg group and 3 patients in each of the other dose groups.

Apatinib DLT occurred in the 3 patients in the 1000 mg group, 2 of whom had grade III hypertension and 1 had grade III hand-foot skin reaction (HFSR). The patients recovered from the toxicity after dose interruption, and subsequently were

administrated a reduced dose to further control toxicity. No DLT occurred in the 6 patients in the 850 mg group. For further safety considerations, the observation time of patients in the 850 mg group was extended to 2 cycles; no DLT occurred. Therefore, 850 mg was determined to be the maximum tolerated dose (MTD).

The major adverse events (AEs) in the first cycle of the tolerance test included HFSR, hypertension, leukopenia, oral mucositis, fever, thrombocytopenia, fatigue, elevated bilirubin, headache, abdominal pain, nausea, and elevated transaminases. Most cases were mild to moderate. The main drug-related side effects included HFSR, leukopenia, hypertension and fever, elevated bilirubin, oesophagitis, skin toxicity, thrombocytopenia, nausea, fatigue, headache, elevated transaminases, oral mucositis, upper abdominal discomfort, tongue pain, hoarseness, stomach discomfort, chest tightness and coughing, proteinuria, and sinus bradycardia. In the 850 mg and lower dose groups, the haematological toxicities, including leukopenia and thrombocytopenia, were grade I-III, and non-haematological toxicities were grade I and II, all of which were cured after symptomatic treatment.

1.4.2 Phase I clinical pharmacodynamics observation of apatinib

From May 2007 to December 2008, a phase I clinical trial on apatinib was carried out at Fudan University Shanghai Cancer Centre; the trial specifically included a tolerance study, a pharmacokinetics study, and a phase I clinical supplementary study. A total of 81 patients with advanced solid tumours who either did not respond to or did not receive standard treatment were enrolled. Among them, efficacy was not evaluated in 9 patients, and the dose of apatinib to another 3 patients was low, only 250 mg/day. A total of 69 subjects received apatinib treatment at 500-1000 mg/day (only 3 subjects received 1000 mg/day), and a total of 56 patients were evaluable. The tumour types in the 69 subjects and the efficacy of apatinib in the 56 evaluable patients are provided in Table 1-2 below:

Table 1-2 The efficacy of apatinib on different solid tumours

	Gastric cancer	Colorectal cancer	Lung cancer	Breast cancer	Nasopharyngeal carcinoma	Kidney cancer	Oesophageal cancer	Liver cancer	Small intestinal stromal tumour	Malignant schwannoma of the left iliac fossa
NE	1	7	1	1	1	0	1	1	0	0
CR	0	0	0	0	0	0	0	0	0	0
PR	2	2	0	0	0	1	0	0	1	0
SD	5	15	3	4	1	0	4	1	0	0
PD	1	7	1	2	0	0	0	0	0	1
Death before evaluation	3	1	0	0	0	0	0	0	0	0
Objective response rate	18.1%	8%	0	0	0	100%	0	0	100%	0
Disease control rate	63.6%	68%	75%	66.7%	100%	100%	100%	100%	100%	0
Total	12	32	5	7	2	1	5	2	1	1

Results:

A total of 69 subjects with various types of solid tumours were included in the statistical analysis; the efficacy for 13 subjects was not evaluable. Among the 69 subjects included in the statistical analysis, the dose was 500 mg for 16 subjects, 750 mg for 37 subjects, 850 mg for 13 subjects, and 1000 mg for 3 subjects.

Based on the ORR, only 1 patient with renal cancer and 1 patients with a small intestinal stromal tumour were enrollable or evaluable, and they both achieved a PR. Eleven evaluable patients with gastric cancer and 25 evaluable patients with colorectal cancer were included, and they achieved high response rates of 18.1% and 8%, respectively.

Based on the disease control rate (DCR), only 1 patient with nasopharyngeal carcinoma, 1 patient with liver cancer, and 1 patient with a gastrointestinal stromal tumour were evaluable, and they all achieved stable disease (SD). Due to the small number of cases, efficacy should be interpreted with caution. Four patients with oesophageal cancer, 4 patients with lung cancer, and 6 patients with breast cancer were evaluable, and their DCRs were 100%, 75%, and 66.7%, respectively. However, due to the small number of cases, efficacy was difficult to evaluate.

1.4.3 Phase I clinical pharmacokinetics study of apatinib

Three doses, namely, 500, 750, and 850 mg/case, were used in single-dose oral administration tests. Seven males with tumours and 5 females with tumours were assigned to the low-dose group, 6 males with tumours and 3 females with tumours were assigned to the medium-dose group, and 6 males with tumours and 6 females with tumours were assigned to the high-dose group. In this study, all patients took apatinib 0.5 h after meals, and blood samples from multiple sites and urine samples were collected within 48 h after administration. The results indicated that M1 is a major metabolite of apatinib in humans; the AUC_{M1}/AUC of apatinib was 1.15-5.06. The 48-h cumulative urinary excretion of apatinib ($Cum.Ae_{M1}$/Cum.Ae) was 24.5-353. The $T_{1/2}$ values for apatinib and M1 were 8.93±0.81 h and 12.5±1.666 h, respectively. At the same dose, the individual differences in the levels of apatinib and M1 exposure (AUC and C_{max}) were large. The high-dose group showed significant sex differences ($P < 0.05$), including an AUC 1.96 times higher in females than in males and a C_{max} 3.57 times higher in females than in males. The exposure levels of apatinib and M1 in male and female subjects were nonlinearly but positively correlated with the oral dose of apatinib.

Food intake effect test: The focus of this test was to compare the difference between the absorption of apatinib by oral administration 1 h before food intake and 0.5 h after food intake. The *in vivo* concentration of M1 was also examined. In this test, blood samples from multiple sites and urine samples were collected within 48 h of the administration of apatinib. The results indicated that there were no significant differences in the blood T_{max} and C_{max} of apatinib between male and female subjects ($P > 0.05$). In addition, the AUC, $T_{1/2}$ and Cum.Ae of apatinib, as well as the related pharmacokinetics parameters of M1, were not affected by the order of food intake and the administration of apatinib ($P > 0.05$).

Because M1 has a longer half-life ($T_{1/2}$: 12.9±1.85 h) than apatinib, the multiple administration test focused on the *in vivo* accumulation of M1. In addition, the concentration of apatinib ($T_{1/2}$: 9.24±1.40 h) was observed during the test. In this test, the frequency of administration of apatinib was once per day, and the test period was 4

weeks. Blood samples from multiple sites and urine samples were collected on days 1, 14 and 28 after the start of the test. The test results indicated that although M1 and apatinib have long half-lives, no significant accumulation of M1 and apatinib ($P > 0.05$) was observed in the body of the subjects on day 14 after the start of the test compared with that on day 1 after the start of the test. However, the M1 exposure levels in male subjects increased significantly ($P < 0.05$) on day 28 compared with day 1. The AUC (1 d), AUC (14 d) and AUC (28 d) of apatinib were 9260±4308, 7256±4709, and 21930±20098 ng·h/ml, respectively. The C_{max} (1d) C_{max} (14 d), and C_{max} (28 d) of apatinib were 1285±776, 693±430, and 1602±1755 ng/ml, respectively. The AUC (1 d), AUC (14 d) and AUC (28 d) of M1 were 13846±7061, 10899±6702, and 39784±21900 ng·h/ml, respectively. The C_{max} (1 d), C_{max} (14 d) and C_{max} (28 d) of M1 were 1193±673, 908±597, and 1728±1233 ng/ml, respectively. There was no significant cumulative urinary and renal excretion of apatinib and M1 ($P > 0.05$). The Cum.Ae (1 d), Cum.Ae (14 d) and Cum.Ae (28 d) of M1 were 30281±8141, 34537±15867, and 33466±17737 μg, respectively.

1.4.4 Safety data of apatinib

The results of the phase I trial of apatinib indicated that patients well tolerated the application of 850 mg apatinib alone, that the application of 250 mg, 500 mg and 750 mg apatinib was safe in patients. The main manifestations of toxicity observed in the phase I trial included hypertension (10/16), hand-foot syndrome (HFS) (7/16), bone marrow suppression (7/16), oral ulcers (5/16), oesophagitis (2/16), chest and back pain (2/16), hoarseness (2/16), fatigue (2/16), diarrhoea (1/16), and proteinuria (1/16). In the 750 mg and lower dose groups, haematological toxicities, including leukopenia and thrombocytopenia, were grade I to III, and non-haematological toxicities were grade I and II; these toxicities were cured after symptomatic treatment. The results of phases II and III gastric cancer trials also confirmed that the main side effects to apatinib are leukopenia (36.36%), neutropenia (34.09%), thrombocytopenia (23.03%), proteinuria (44.32%), hypertension (35.23%), HFS (27.84%), fatigue (18.18%), loss of appetite (10.23%), diarrhoea (8.52%), and hoarseness (7.93%). The incidence of unexpected

side effects was low in the trials. Most side effects were transient or reversible after dose adjustment or drug discontinuation, and all of them were controllable.

Based on the results of clinical trials, the Food and Drug Administration of China (CFDA) approved apatinib for the treatment of advanced gastric cancer on November 17, 2014, becoming the first small-molecule targeted drug approved for the treatment of advanced gastric cancer in the world thus far.

In summary, based on the current research status and clinical need for antiangiogenic drugs to treat recurrent PROC, it is necessary to further explore the efficacy and safety of apatinib for the treatment of recurrent PROC.

2. Study purpose

(1) To clarify the efficacy of apatinib in patients with first recurrence of PROC.

(2) To clarify the molecular subtypes of the population who benefits from apatinib and screen the molecular markers that can assess the efficacy of apatinib, thus assisting in the selection of the target population for apatinib.

(3) To study the safety of apatinib, collect relevant safety information in the ovarian cancer population, and observe side effects.

2.1 Primary outcome measures

Progression-free survival (PFS) is defined as the time from randomization to tumour progression or death due to any cause.

2.2 Secondary outcome measures

- Overall survival (OS), defined as the time from the date of randomization to death (months). For subjects who are still alive or lost to follow-up by the cut-off date for data analysis, survival will be censored based on the last known survival time of the subject.
- ORR, defined as the proportion of patients whose tumour volume is reduced to a predetermined value. The response period refers to the period from the first time of

patients achieving CR or PR to disease progression. The imaging results will be evaluated according to the RECIST 1.1 criteria and classified as complete response (CR), partial response (PR), SD, and progressive disease (PD). The ORR is (CR+PR)%.

- DCR, defined as the proportion of the number of patients that have achieved a response (PR+CR) and SD after treatment to the number of evaluable cases. That is, DCR=CR+PR+SD.
- Safety.

3. Study design

This is a randomized, parallel-controlled, multicentre, phase II study. The planned number of enrolled patients is 126. Patients who meet the enrolment criteria will be randomly allocated to one of the following treatment regimens:

1) Doxorubicin hydrochloride liposomes 40 mg/m^2 D1 Q4W; or

2) Doxorubicin hydrochloride liposomes 40 mg/m^2 D1 Q4W plus apatinib (500 mg po qd)

The stratification factor is platinum-free interval (≤3 vs. >3 months).

This study will be divided into 3 stages:

1. The baseline period (within 21 days before the start of treatment) – Patients will complete the screening examination during the baseline period to assess whether they meet the inclusion criteria;

2. Treatment period (from the first administration of apatinib to the completion of the last treatment cycle) – Tumours were evaluated every 8 weeks;

3. Follow-up period – After the end of the study treatment, the survival status and the follow-up antitumour treatment data will be collected by telephone follow-up or patient visits to the research centre every 3 months until death or loss to follow-up.

4. Subjects

4.1 Inclusion criteria

1. Previous pathological diagnosis of ovarian cancer, fallopian tube cancer, or

primary peritoneal cancer, and available paraffin sections from a previous surgery;

2. PROC that first reoccurs less than 6 months after the last chemotherapy;

3. Combined with malignant pleural effusion or ascites or with clinically evaluable recurrent lesions;

4. Eastern Cooperative Oncology Group (ECOG) performance status score of 0-1;

5. Expected survival ≥ 4 months;

6. No history of anti-vascular targeted therapy;

7. At least 1 measurable lesion as the target lesion based on the Response Evaluation Criteria in Solid Tumours (RECIST 1.1) criteria; if the target lesion is a lymph node, the diameter must be greater than 1.5 cm, and it must be unsuitable for surgical treatment; and the target lesion must be free of radiotherapy or relapsed within the radiotherapy field;

8. Baseline complete blood count meets the following criteria:

a) Neutrophil count ≥ 1.5 x 10^9/L;

b) Platelet (PLT) count ≥ 100 x 10^9/L; and

c) Haemoglobin ≥ 9 g/dL (allowing blood transfusion to achieve or maintain this level).

9. Liver function meets the following criteria:

a) Total bilirubin <1.5 times the upper limit of normal (ULN); and

b) AST and ALT < 2.5 × ULN in patients without hepatic metastasis and < 5 × ULN in patients with liver metastases; and

10. Serum creatinine ≤ 1.25 × ULN or the calculated creatinine clearance ≥ 50 mL/min.

4.2 Exclusion criteria

1. Having received more than 2 chemotherapy regimens;

2. Refractory patients progressing during previous treatment;

3. Currently using or recently used (within 30 days prior to enrolment) another investigational drug or participating in another clinical study;

4. Other malignancies occurred within 5 years (except for fully treated cervical

carcinoma in situ or skin squamous cell carcinoma or controlled basal cell carcinoma of the skin);

5. Hypertension that cannot be resolved, i.e., out of the normal range, by antihypertensive drug treatment (systolic blood pressure (SBP) ≥ 140 mmHg or diastolic blood pressure (DBP) ≥ 90 mmHg);

6. Grade II or higher myocardial ischaemia or myocardial infarction and poorly controlled arrhythmia (including heart rate-corrected QT (QTc) ≥ 450 ms for males and QTc ≥ 470 ms for females);

7. Previous or current cardiac insufficiency of grade II or above based on the New York Heart Association (NYHA) criteria or left ventricular ejection fraction (LVEF) lower than 50% or the lower limit of normal as evidenced by echocardiography;

8. Coagulation disorders [international normalized ratio (INR) >1.5, prothrombin time (PT) >ULN + 4 s or activated partial thromboplastin time (APTT) >1.5 × ULN], bleeding tendency, or current thrombolysis or anticoagulation therapy;

9. Clinically significant bleeding symptoms or clear bleeding tendency, such as gastrointestinal bleeding, haemorrhagic gastric ulcer, baseline faecal occult blood of ++ or above, and vasculitis, within 3 months before randomization;

10. Major surgery or severe traumatic injury, fracture or ulcer within 4 weeks before randomization;

11. Factors that significantly affect the absorption of oral drugs, such as inability to swallow, chronic diarrhoea, and intestinal obstruction;

12. Urinary protein ≥ ++ indicated by urinalysis or 24-h urinary protein ≥ 1.0 g;

13. Other conditions that may affect the clinical study or the interpretation of the study results;

14. Allergy to doxorubicin and/or related substances or the presence of idiosyncratic reactions;

15. The expected cumulative dose of doxorubicin (including previous anthracyclines, if any) reaches or exceeding 550 mg/m^2 after 4 courses of doxorubicin hydrochloride injection;

16. Uncontrollable arrhythmia or other ECG abnormalities indicative of research

risk, as determined by the principal investigator;

17. Doxorubicin liposome treatment in the past 6 months; and

18. History of local radiation therapy.

4.3 Criteria for termination or withdrawal

1. Patients can freely withdraw from the study at any time without any reason;

2. Concurrent radiotherapy on target lesions for efficacy observation;

3. Violation of the protocol that affects the evaluation of the results;

4. The investigators terminate the study due to safety events among the enrolled patients (such as intolerable AEs, pregnancy during treatment, or other events that affect the safety of the subjects).

5. When the inclusion/exclusion criteria are violated, or the subject lacks compliance with or has to use unauthorized drugs, the investigator and/or sponsor can decide to discontinue the treatment;

6. PD;

7. Medical or ethical reasons that affect the continuation of the study;

8. Poor quality data and incomplete and inaccurate information;

9. The patients receive other antitumour drugs during the clinical study period; and

10. The sponsor terminates the study.

Once a subject withdraws, the investigator will record the reason for withdrawal on the subject's case report form (CRF) and medical record. All subjects who withdraw due to AEs or abnormal clinical laboratory test results will followed up until the subjects recover or achieve SD, and the subsequent outcomes will be recorded. The efficacy will not be evaluated for participants who withdraw due to side effects, but their adverse drug reactions (ADRs) will be included in the statistics.

4.4 Relevant post-withdrawal regulations for patients in the monochemotherapy group

After patients with doxorubicin liposomal monochemotherapy withdraw from the

group due to disease progression during treatment, they can select the paclitaxel monochemotherapy regimen at 80 mg/m^2 qw based on their actual clinical needs. If patients have no contraindications for apatinib and have the desire to use apatinib, they can receive apatinib in combination with the paclitaxel monochemotherapy regimen under the guidance and follow-up of the clinicians following the same medication principle used for the group receiving combined therapy. Apatinib will be given to these patients free of charge. During treatment, patients will be encouraged to pay close attention to side effects and record them and to undergo regular laboratory tests. The researchers will provide close follow-up regarding the side effects and laboratory indexes of patients and provide corresponding treatments in a timely manner.

4.5 Early termination of the research project

If any of the following situations occur, the study can be terminated early after a discussion and judgement by the project team:

1) New information leads to adverse risk-benefit outcomes for the investigational drug, such as obtaining evidence that the investigational treatment is ineffective and observing any significant but previously unknown side effects or unexpected increases in the severity or incidence of known side effects or other evidence of poor safety;

2) The project undertaker believes that the research cannot be continued for both medical and ethical considerations; and

3) The investigational drug is withdrawn from the market for safety reasons.

5. Study schedule and evaluation

A signed and dated ICF will be obtained from each patient before any screening procedure is performed. The ICF will be reviewed and approved by an ethics committee.

5.1 Screening examination and baseline evaluation (will be completed within 21 days before the start of treatment)

After the patients sign the ICF, the following items will be examined to determine

whether they meet the inclusion criteria:

Evaluation items	Key inspection items	Time from evaluation to the first dose
1. Written informed consent form	Signature	Signed before the first dose
2. Disease history and physical examination	The medical history should include the present medical history, past medical history, and history of allergies; the physical examination should include a pelvic examination.	7 days
3. CBC (venous blood)	Haemoglobin, platelet count, white blood cell count, neutrophil count	3 days
4. Biochemical profile	Blood biochemistry (ALT, AST, AKP, BIL, BUN, and Cr)	7 days
5. Urinalysis	Urine pH, urinary protein, urinary red blood cells, and urinary white blood cells	14 days
6. Stool test	Occult blood, red blood cells, and white blood cells	14 days
7. Pregnancy test	Pregnancy test (test of urine β-HCG or blood β-HCG in women of childbearing age who have not undergone total hysterosalpingo-oophorectomy)	14 days
8. Tumour evaluation	CT or MRI	21 days
9. Tumour marker	CA125	7 days
10. Quality of Life evaluation	EORTC QLQ-C30	7 days
11. ECG	Heart rate, heart rhythm, QTc interval	7 days
12. Cardiac function	Echocardiography	21 days

5.2 Evaluation of the treatment period

The combination regimen will be applied for a maximum of 6 cycles. After the completion of the combination regimen, patients with CR, PR or SD will be treated with apatinib monotherapy. The application of apatinib monotherapy will be continued until disease progression or intolerance.

1. The following examinations and evaluations will be completed 5-7 days before

the start of each treatment cycle:

(1) CBC, biochemical, and coagulation tests;

(2) Urinalysis and stool test;

(3) Evaluation of tumour markers;

(4) Concomitant medication evaluation;

(5) Physical examination (including body weight) and vital signs;

(6) ECOG physical performance status evaluation;

(7) 12-lead ECG and echocardiography;

(8) Evaluation of peripheral neurotoxicity; and

(9) Evaluation of AEs and clinical toxicity.

2. During treatment, CBC and urinalysis will be performed at least once per week.

3. Contrast-enhanced thoracic-abdominal-pelvic CT examinations will be performed before and after the first, third, and fifth cycles of treatment:

Tumour evaluations will always use the same method used for the baseline evaluation. If possible, for patients who have achieved an objective response (CR or PR), efficacy will be confirmed no less than 4 weeks after the first response.

4. The number of CBCs, blood biochemical tests, and urinalyses can be increased based on clinical needs. If the results are out of the normal range, the investigators will re-examine the results as appropriate. Any treatment-related abnormality in clinical examination or laboratory test results will be followed until the abnormality subsides or has no clinical significance.

5.3 End-of-study evaluation

When a patient decides to exit the study due to disease progression, the following evaluations will be performed when the patient withdraws from the trial:

1. Concomitant medication evaluation;

2. Physical examination;

3. ECOG performance status score;

4. Evaluation of peripheral neurotoxicity;

5. Evaluation of AEs;

6. 12-lead ECG and echocardiography;

7. Clinical laboratory tests (including CBC, coagulation, blood biochemistry, tumour markers, urinalysis, stool test, etc.); and

8. Imaging examination of tumours (CT or MRI).

5.4 Follow-up

All reasons for and dates of study discontinuation will be recorded in the CRF (e.g., loss to follow-up, patient refusal, and AEs). AE reporting will be completed within 30 days after the last dose of the investigational drug. The AEs related to the investigational drug will be monitored, and the outcomes will be recorded until the AEs resolve, until the correlation does not hold, or until the investigators judge that no further follow-up is needed from the medical point of view. The corresponding treatments for these AEs will be recorded in the CRF. After the evaluation, each enrolled patient will enter the follow-up period. The study physicians will collect information on survival status by phone follow-up or patient visits to the research centre every 2-3 months.

5.5 Evaluation of early exit and withdrawal

Patients who request early exit during the treatment period will receive a study exit evaluation. The reason for and date of exit will be recorded in the patient's medical record and CRF. When a patient withdraws from the study, the investigators will make every effort to complete all withdrawal-related examinations.

5.6 Study duration

The enrolment period is planned to be 12 months. The end of the study is defined as the completion of at least 2 efficacy evaluations for the last subject under continuous treatment or the occurrence of disease progression or intolerable toxicity.

5.7 Study schedule

		**During treatment**

Evaluation content	Screening period[1]	1st cycle	2nd cycle	3rd cycle	4th cycle	5th cycle	xth cycle	(x+1)th cycle	…….[8]
	(-21 ±5 days)	Day 28±5[2]	Day 56±5	Day 84±5	Day 112±5	Day 140±5	Day 28x±5	Day 28(x+1)±5	Efficacy will be evaluated once every 2 cycles
Written informed consent form	✓								
Demographics	✓								
Medical history	✓								
Inclusion/exclusion criteria	✓								
**Efficacy evaluation**									
Disease evaluation/tumour measurement (CT, MRI, etc.)	✓		✓		✓		✓		✓ Within the last 5 days of even-numbered cycles
Tumour marker (CA125)	✓	✓	✓	✓	✓	✓	✓	✓	✓ Within the last 5 days of even-numbered cycles
EORTC QLQ-C30 (V3.0) Chinese version	✓		✓		✓		✓		✓
**Safety evaluation**									

Concomitant medication	✓	✓	✓	✓	✓	✓	✓	✓	✓ Record at any time
Physical examination[3]	✓		✓		✓		✓		✓
Vital signs	✓	✓	✓	✓	✓	✓	✓	✓	✓
ECOG performance status	✓	✓	✓	✓	✓	✓	✓	✓	✓
Weight	✓	✓	✓	✓	✓	✓	✓	✓	✓
Height	✓								
ECG	✓	✓	✓	✓	✓	✓	✓	✓	✓
Echocardiography[4]	✓	✓	✓	✓	✓	✓	✓		
**Laboratory evaluation**									
CBC[5]	✓	✓	✓	✓	✓	✓	✓	✓	✓
Urinalysis[5]	✓	✓	✓	✓	✓	✓	✓	✓	✓
Biochemical profile[6]	✓	✓	✓	✓	✓	✓	✓	✓	✓
Stool test[7]	✓		✓		✓		✓		✓
Prothrombin time[8]	✓	✓	✓	✓	✓	✓	✓	✓	✓
**Investigational drug**									
Doxorubicin hydrochloride liposome		✓	✓	✓	✓	✓	✓		
Apatinib		✓	✓	✓	✓	✓	✓	✓	✓
Adverse event observation	Recorded at any time until 5 days after the end of treatment								
Study termination[10]	Record at any time								

Note:

1. Screening will be evaluated within a specified time frame before the first use of the investigational drug.

2. Laboratory evaluations will be carried out within 5 days before the use of investigational drugs but will be repeated only when clinically necessary.

3. Physical examinations will be performed within 7 days before the visit at the earliest.

4. Echocardiography can be discontinued after treatment with doxorubicin hydrochloride

liposomes.

5. The number of CBCs [haemoglobin, white blood cell (WBC) count, absolute neutrophil count (ANC), and platelet (PLT) count] and urinalyses (urinary pH, urine protein, urinary red blood cells, and urinary WBC) can be increased based on clinical needs. CBC should be performed within 3 days before each treatment.

6. The number of blood biochemical tests, including liver function tests (ALT, AST, and TBIL), renal function tests (BUN and Cr), and electrolyte tests (serum potassium, sodium, and chloride) can be adjusted based on clinical needs. The laboratory screening results will be reviewed. If the results are out of the normal range, the investigator will re-examine the results as appropriate. Subjects exhibiting treatment-related abnormalities in clinical examinations or laboratory tests will be followed up until the abnormalities have subsided or are no longer clinically significant.

7. Examinations will be performed once during the screening period and then performed as needed by the investigators.

8. Apatinib (500 mg, qd) will be administered until disease progression or intolerable toxicity.

9. The combination regimen can be used for a maximum of 6 cycles. After the completion of the combination regimen, patients with CR, PR or SD will be treated with apatinib monotherapy. Apatinib monotherapy will be continued until disease progression or intolerance.

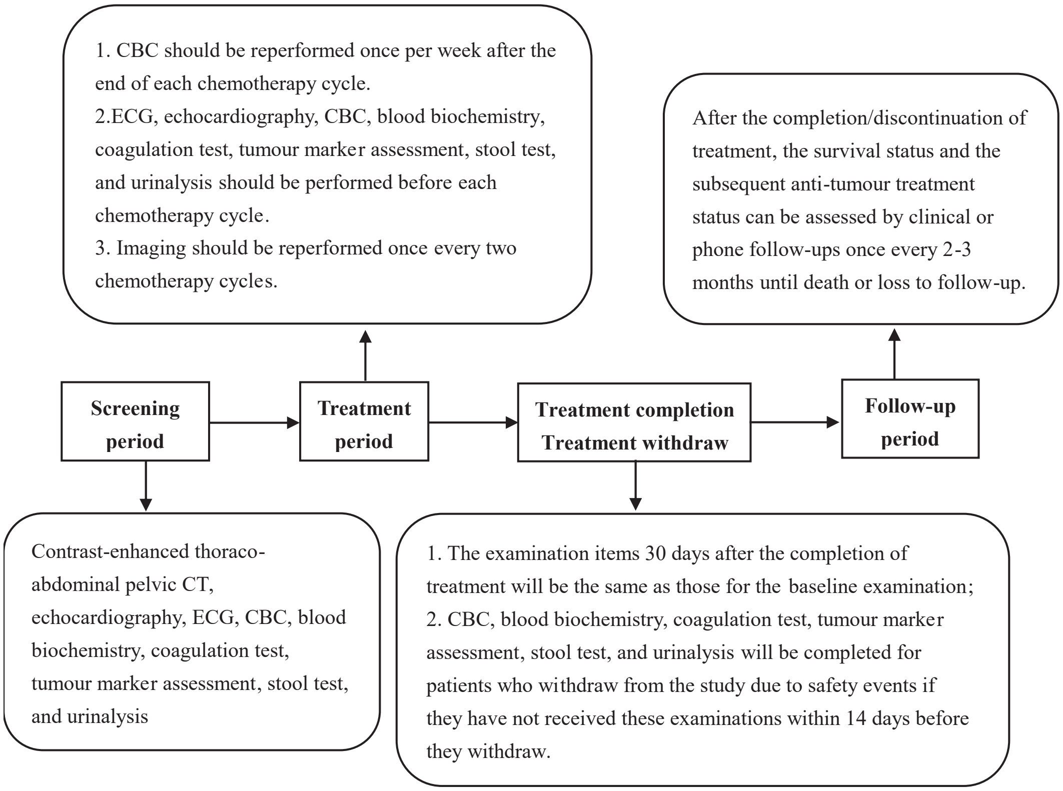

6. Investigational drug

6.1 Treatment regimens

The 126 patients were divided into 2 groups, the patients were randomly assigned to the following 2 treatment regimens by 1:1:

Group A: doxorubicin hydrochloride liposomes 40 mg/m^2 D1 (1 course of treatment = 28 days) + apatinib (500 mg po qd) * 6 cycles; the combination regimen can be applied for a maximum of 6 cycles. After the completion of the combination regimen, patients with CR, PR or SD will be treated with apatinib monotherapy. Apatinib monotherapy will be continued until disease progression or intolerance; and

Group B: doxorubicin hydrochloride liposomes 40 mg/m^2 D1 (1 course of treatment = 28 days) * 6 cycles.

6.2 Dose adjustment

6.2.1 Criteria for drug discontinuation or dose reduction

If a patient has side effects between 2 sequential follow-up visits, the patient should contact the doctor as soon as possible to determine the next treatment regimen. Drug discontinuation and dose reduction are only allowed when haematological toxicity reaches grade III or non-haematological toxicity reaches grade II or above. For non-haematological toxicity, nausea, vomiting and fever with definite cause (below 38℃) should be actively treated symptomatically without drug discontinuation or a dose reduction.

If a severe adverse reaction occurs, the dose will be adjusted or paused until the next cycle. If severe haematological and non-haematological toxic reactions occur, the dose of the drug with a significant causal relationship with the toxic reaction will be adjusted; the dose of only one drug will be adjusted per cycle. The dose of doxorubicin hydrochloride liposomes for intravenous chemotherapy will only be reduced twice (the dose will be reduced by 25% of the standard dose each time), and the dose of apatinib for oral chemotherapy will only be reduced twice (from 500 mg, po, qd to 250 mg, po, qd and from 250 mg, po, qd to 250 mg, po, qod). Otherwise, the patient must withdraw from the study.

During treatment, if a subject has not recovered from a drug toxicity, drug administration will be suspended. The cumulative duration of drug discontinuation for each dosing cycle will not exceed 2 weeks, and the drug discontinuation will not exceed 2 times within each cycle to maintain the drug concentration in each subject who receives treatment. The dose will be delayed for no more than 2 weeks; otherwise, the subject must withdraw from the study.

If multiple toxic reactions occur in 1 treatment cycle, the dose will be adjusted based on the highest grade of toxic reactions. For patients who receive combination therapy, if a toxic reaction is related to 2 drugs, the dose of oral apatinib will be reduced first. After the 1-time apatinib dose reduction, if the toxic reaction persists or is not reduced to grade 1, the dose of doxorubicin hydrochloride liposomes will be reduced no more than 2 times.

6.2.2 Details of drug discontinuation or dose reduction

To ensure the consistency of dose adjustments throughout the study, drug

discontinuation will occur before dose reductions in each dosing cycle. The rules for drug discontinuation and dose reductions are provided in the table below (as long as the conditions for dose reduction are met, the drug is considered poorly tolerated, and the dose must be reduced).

Rules for dose adjustment

NCI Common Toxicity Criteria	Dose adjustment for haematological toxicity
Grade I haematological toxicity	Maintain the original dose level (appropriate symptomatic treatment)
Grade II haematological toxicity	Maintain the original dose level (appropriate symptomatic treatment)
Grade III haematological toxicity	First, the drug will be suspended for symptomatic treatment. When toxicity decreases to ≤ grade II, the dose will be reduced by 1 level, and administration will be resumed.
Grade IV haematological toxicity	First, the drug will be suspended for symptomatic treatment. When the toxicity decreases to ≤ grade II, the dose will be reduced by 1 level, and administration will be resumed.
	Dose adjustment for non-haematological toxicity
Grade I non-haematological toxicity	Maintain the original dose level (appropriate symptomatic treatment)
Grade II non-haematological toxicity	Symptomatic treatment will be performed without discontinuing the drug. If the toxicity decreases to ≤ grade I 2 weeks after symptomatic treatment, drug administration will continue; if the toxicity does not improve or is aggravated, drug administration will be suspended, and the treatment will be continued at the original dose level when the toxicity decreases to ≤ grade I.
Grade III non-haematological toxicity	The drug will be suspended for symptomatic treatment. When the toxicity decreases to ≤ grade I, the treatment will be continued at the original dose, or the investigators may reduce the dose by 1 level as appropriate. If grade III or above side effects reoccur, the treatment will be discontinued, and the subject should withdraw from the clinical study.
Grade IV non-haematological toxicity	Drug administration will be suspended for symptomatic treatment. When the toxicity decreases to ≤ grade I, the treatment will be continued at the original dose. If grade IV toxicity reoccurs, the treatment will be discontinued, and the subject should withdraw from the clinical study (if life-threatening side effects, including grade IV renal damage, neurotoxicity, heart toxicity, and liver toxicity, occur, the treatment will be immediately discontinued, and the subject should withdraw from the clinical study).
Side effects that require special attention	
If hypertensive crises, cerebral haemorrhage, ≥ grade II pulmonary haemorrhage, ≥ grade III haemorrhage elsewhere, arterial thrombosis, grade IV venous thrombosis, leukoencephalopathy syndrome, or gastrointestinal perforation occur, the treatment will be immediately discontinued, and the subject should withdraw from the clinical study and receive active symptomatic treatment.	

6.2.3 Symptomatic treatment and dose adjustment schemes for common side effects

6.2.3.1 Non-haematological side effects

1) HFS:

During the administration of apatinib, the subjects should avoid mechanical injury and friction on the palms and soles by wearing loose and breathable shoes and soft cotton gloves and socks and using gel insoles and avoid physically strenuous exercise. The exposure of the hands and feet to high heat and direct sunlight should be avoided. The skin should be protected using moisturizers that contain lanolin or urea. Spicy foods should be avoided. If moderate HFSR occurs, some necessary supportive and symptomatic treatments can be provided, e.g., routine skin care, cleansing and moisturization, the avoidance of secondary infection, compression, mild abrasives, and mild moisturizer or lubricants. Topical exfoliators, including urea ointments, urea creams, 5% salicylic acid preparations, and corticosteroid-containing emulsions or lubricants can be applied. If necessary, the affected skin area can be soaked in magnesium sulphate dissolved in warm water. Topical antifungal or antibiotic treatment can be applied. B vitamins (B1, B6, and riboflavin) and celecoxib can be administered as appropriate.

If HFS occurs during the treatment period, the treatment dose can be adjusted or delayed based on the scheme detailed in Table 1.

Table 1 Dose adjustment scheme for hand-foot syndrome (HFS)

Toxicity grade	Dose adjustment
Grade 1 (mild erythema, oedema, or peeling that does not interfere with daily activities)	The dose will be continued unless the patient has a history of grade 3 or 4 HFS. If the patient has a history of grade 3 or 4 HFS, administration will be delayed for 2 weeks, and the dose will be reduced once before the normal dose interval is resumed.
Grade 2 (erythema, oedema, or peeling that interferes with but does not prevent normal activities; small blisters or ulcers less than 2 cm)	The dose will be delayed for 2 weeks or until the symptoms return to grade 0 or 1. If the symptoms do not improve after 2 weeks, the drug will be discontinued. If the symptoms decrease to grade 0 or 1 within 2 weeks and there is no history of grade 3 or 4 HFS, the previous dose and interval will be continued. If the patient has a history of grade 3 or 4 hand-foot-mouth syndrome, the dose will be reduced once before the normal dosing interval is resumed.

Grade 3 (fever, ulcer or swelling that interferes with walking or normal activities and prevents the participant from dressing normally)	The dose will be delayed for 2 weeks or until the symptoms decrease to grade 0 or 1. The dose will be reduced once before the normal dosing interval is resumed. If the symptoms do not improve after 2 weeks, the drug will be discontinued.
Grade 4 (spread or local infection, or bedridden or hospitalized)	The dose will be delayed for 2 weeks or until the symptoms decrease to grade 0 or 1. The dose will be reduced once before the normal dosing interval is resumed. If the symptoms do not improve after 2 weeks, the drug will be discontinued.

2) Oral mucositis:

The subjects are recommended to maintain their oral hygiene during the study period, rinse their mouth with salt water after meals, and brush their teeth before going to bed. Patients with mild oral mucositis should avoid eating hard, cold, hot, and spicy food. For moderate oral mucositis, dose adjustment is not required, and topical antibacterial agents and mucosal protective agents (sucralfate, honey, aloe vera) will be used for symptomatic treatment. For severe oral mucositis, doxorubicin hydrochloride liposomes will be discontinued. For patients with severe ulcers, compound chlorhexidine and dexamethasone pellicles will be used. For patients with severe pain, local anaesthetics will be used as appropriate for pain relief, and 1% lidocaine can be used as a gargle. If oral mucositis occurs during the treatment period, the treatment dose can be adjusted or delayed based on the scheme detailed in Table 2.

Table 2 Dose adjustment scheme for oral mucositis

Toxicity grade	Dose adjustment
Grade 1 (painless ulcer, erythema, or mild pain)	The dose will be continued unless the patient has a history of grade 3 or 4 oral mucositis. If the patient has a history of grade 3 or 4 oral mucositis, the dose will be delayed for 2 weeks. The dose will be reduced once before the normal dosing interval is resumed.
Grade 2 (painful erythema, oedema or ulcer but able to eat)	The dose will be delayed for 2 weeks or until the symptoms decrease to grade 0 or 1. If the symptoms do not improve after 2 weeks, the drug will be discontinued. If the symptoms decrease to grade 0 or 1 within 2 weeks and the patient has no history of grade 3 or 4 oral mucositis, then treatment will resume with the previous dose and interval. If the patient has a history of grade 3 or 4 oral mucositis, the dose will be reduced once before the normal dosing interval is resumed.

Grade 3 (painful erythema, oedema, or ulcer that causes an inability to eat)	The dose will be delayed for 2 weeks or until the symptoms decrease to grade 0 or 1. The dose will be reduced once before the normal dosing interval is resumed. If the symptoms do not improve after 2 weeks, the drug will be discontinued.
Grade 4 (necessity for enteral or parenteral nutrition support)	The dose will be delayed for 2 weeks or until the symptoms decrease to grade 0 or 1. The dose will be reduced once before the normal dosing interval is resumed. If the symptoms do not improve after 2 weeks, the drug will be discontinued.

3) Hypertension:

The US National Cancer Institute (NCI) cardiovascular toxicity research group recommends that the baseline blood pressure of subjects should be determined before the start of treatment with VEGF/VEGFR inhibitors. During treatment, every effort will be made to keep blood pressure stable and <140/90 mmHg. Blood pressure monitoring will be initiated before dosing and throughout the entire treatment process, especially during the initial 2 weeks of treatment, when daily monitoring will occur. The subjects will be fully informed when their blood pressure is >140/90 mmHg, and when subject experience symptoms associated with increased blood pressure (such as headache, dizziness, and visual impairment), they should contact the investigators immediately for guidance.

The selection of antihypertensive drugs will follow the relevant guidelines for the prevention and treatment of hypertension, with reference to the risk of cardiovascular events for the subjects. For patients with proteinuria, angiotensin-converting enzyme inhibitors (ACEIs) and angiotensin receptor antagonists (ARBs) will be recommended. Regarding drug selection, it should be noted that apatinib is mainly metabolized by the CYP3A4 enzyme in the liver; therefore, non-dihydropyridine calcium channel blockers (CCBs, such as verapamil and diltiazem) that can inhibit the CYP3A4 system are not recommended for blood pressure control. If grade 3-4 hypertension occurs, antihypertensive treatment will be actively carried out under the guidance of a cardiovascular specialist, and the dose of apatinib will be closely observed and/or adjusted. If hypertension is still not well controlled, drug administration should be stopped. For patients in a hypertensive crisis, the drug will be discontinued immediately and permanently.

For patients with hypertension during the treatment period, the dose can be adjusted or delayed based on the scheme detailed in Table 3.

Table 3 Hypertension criteria (NCI-CTCAE 4.03) and prevention and treatment recommendations

Grade	Definition	Prevention and treatment recommendations
1	SBP = 120-139 mmHg<br>or DBP = 80-89 mmHg	Blood pressure will be closely monitored. Salt intake should be limited, and smoking and alcohol use should be stopped. The administration of apatinib will continue without dose adjustment.
2	SBP = 140-159 mmHg<br>or DBP = 90-99 mmHg	Blood pressure will be monitored closely. The administration of apatinib will continue without dose adjustment. Antihypertensive drugs (ACEI/CCB/ARB antihypertensive drugs are recommended) will be used for treatment and should not be discontinued without medical advice.
3	SBP ≥ 160 mmHg,<br>or DBP ≥ 100 mmHg	The administration of apatinib will be suspended. Cardiovascular specialists will be consulted for treatment, and the combined use of antihypertensive drugs will be considered. Blood pressure will be monitored closely. If the drug is postponed for more than 2 weeks, the subject should stop the medication and withdraw from the study. If the blood pressure returns to normal and is well controlled within 2 weeks, the dose of apatinib will be reduced once. If grade 3 or above hypertension occurs, the subject should stop the medication and withdraw from the study.
4	Life-threatening (malignant hypertension or persistent nerve damage, hypertensive crisis)	The administration of apatinib will be discontinued immediately and permanently. Cardiovascular specialists will be consulted for the active management of hypertension, and blood pressure and other vital signs will be monitored closely.

4) Proteinuria:

If a subject has grade 2 or above proteinuria while receiving apatinib, apatinib will be suspended until recovery to normal. If grade 2 or above proteinuria reoccurs after resuming the medication, the dose of apatinib will be reduced by 1 dose unit; if the proteinuria persists and worsens, the medication will be discontinued. Once renal dysfunction or nephrotic syndrome occurs, the medication will be discontinued immediately, and supportive and symptomatic treatment will be provided.

Currently, there is no clear treatment regimen for anti-angiogenesis inhibitor-

induced proteinuria. However, ACEIs and ARBs can be used as appropriate because these drugs can reduce proteinuria and possible adverse cardiac events by reducing intratubular pressure.

When proteinuria occurs during the treatment period, the dose can be adjusted or delayed based on the scheme detailed in Table 4.

Table 4 Proteinuria criteria (NCI-CTCAE 4.03) and prevention and treatment recommendations

Grade	Definition	Prevention and treatment recommendations
1	Urine protein (+) or 24-h urine protein < 1.0 g	The administration of apatinib will continue without a dose adjustment. The condition of the patient will be observed closely.
2	Urine protein (++) or 24-h urine protein = 1.0-3.4 g	The administration of apatinib will continue without a dose adjustment. Drug intervention will be considered. The 24-h urinalysis results and 24-h urine protein will be monitored.
3	24-h urine protein > 3.4 g	Apatinib administration will be suspended. Specialists in nephrology will be consulted. Drug intervention will be performed. After the proteinuria decreases to grade 2 or below, apatinib will be continued at a reduced dose. If grade 3 proteinuria still occurs, apatinib treatment will be permanently discontinued.

5) Bleeding

During the administration of apatinib, PT and INR will be closely monitored, and bleeding tendencies and related symptoms will be closely monitored. If a serious abnormality (grade 3-4) occurs, drug administration will stop. If upper gastrointestinal bleeding occurs, apatinib will be discontinued immediately, and the bleeding will be treated in accordance with acceptable clinical practices.

6) Thrombosis

If any arterial thrombosis occurs (such as cerebral ischaemia, stroke, angina pectoris, and myocardial infarction), drug administration will be discontinued immediately, and the subject should withdraw from the study. If grade IV symptomatic venous thrombosis occurs, the drug will be discontinued, and the subject should withdraw from the study. Symptomatic treatment, surgery, or anticoagulant medication will be applied immediately to treat thrombotic symptoms.

The dose adjustment scheme for venous thrombosis is provided in Table 5.

Table 5 Dose adjustment scheme for venous thrombosis

Venous thrombosis	Dose adjustment rules
Grade II (uncomplicated deep venous thrombosis that requires medical intervention)	The original dose of apatinib will be maintained, and the condition of the patient will be monitored closely.
Grade III (uncomplicated venous pulmonary embolism or non-embolic cardiovascular thrombosis that requires medical intervention) or grade IV (pulmonary embolism, cerebrovascular events, arterial insufficiency, or haemodynamic or neurological dysfunction that requires acute intervention)	1) The administration of apatinib will be suspended.<br>2) Anticoagulants (small-molecular-weight heparins) will be administered.<br>3). The anticoagulants will be administered for at least 1 week. After the thrombotic symptoms improve without severe bleeding (grade III or IV), apatinib will be continued at a reduced dose (1 dose level less) based on the investigator's judgement.

6.2.3.2 Haematological toxicity

The common haematological side effects to doxorubicin hydrochloride liposomes include leukopenia and thrombocytopenia. When mild haematological toxicity occurs, dose adjustment is unnecessary, and regular examinations will be implemented. For moderate or severe haematological toxicity, drug administration will be stopped immediately, and symptomatic treatment will be provided. If haematological toxicity decreases, drug administration will resume with a reduced dose.

Granulocyte colony-stimulating factor (G-CSF) promotes the differentiation of bone marrow stem cells and the proliferation of granulocytes, and it is currently the preferred drug for the treatment of leukopenia. Patients with thrombocytopenia will be treated with platelet growth-promoting factors, such as recombinant human interleukin-11 (rhIL-II) and recombinant human thrombopoietin (rhTPO). For patients with treatment-induced marked short-term thrombocytopenia, low-dose glucocorticoid treatment can be used. In severe cases, platelet transfusion will be required.

If haematological toxicity occurs during the treatment period, the dose can be adjusted or delayed based on the scheme detailed in Table 6. When the ANC count is

less than 1000/mm^3, G-CSF or granulocyte-macrophage colony-stimulating factor (GM-CSF) can be administered concurrently to maintain the number of blood cells.

Table 6 Dose adjustment scheme for haematological toxicity

Toxicity grade	ANC (cells/mm^3)	PLT count (/mm^3)	Dose adjustment
1	1500 – 1900	75000 – 150000	Treatment will continue without dose adjustment.
2	1000 – < 1500	50000 – < 75000	Treatment will be suspended until ANC > 1500 and PLT count > 75000. Then, the treatment will continue at the original dose.
3	500 – < 1000	25000 – < 50000	Treatment will be suspended until ANC > 1500 and PLT count > 75000. Then, the treatment will continue at the original dose.
4	< 500	< 25000	Treatment will be suspended until ANC > 1500 and PLT count > 75000. Then, the dose will be reduced once, or the treatment will be continued at the original dose together with supportive cytokine therapy.

6.3 Concomitant therapy

During the study period, some drugs can be used concomitantly based on the needs and the usage conditions. When the investigational drug causes an AE that needs to be treated, symptomatic drugs can be provided. When the chemotherapeutic drug causes toxic side effects, such as leukopenia, symptomatic drugs can be provided. Anti-vomiting agents can be used to treat chemotherapy-induced vomiting or given in advance to prevent nausea and vomiting. Symptoms due to other causes can be treated with symptomatic drugs. All concomitantly used drugs will be recorded and described on the CRF.

During the drug administration period, other oncotherapy drugs (including other chemotherapeutic drugs, traditional Chinese medicines with antitumour indications, and thymosin) cannot be used.

6.4 Drug policy

Apatinib (investigational drug): The group A will be given apatinib free of charge for the first 3 cycles.

Doxorubicin liposomes: A third cycle is free of charge for every 2 cycles purchased.

Blood genetic testing: This testing is free of charge.

7. Study endpoint evaluation

7.1 Efficacy evaluation

The efficacy measures include PFS, ORR, DCR, and OS. Tumour efficacy will be evaluated based on the RECIST 1.1 criteria.

PFS: PFS will be calculated from the time of randomization to disease progression or death.

ORR and DCR: Tumour imaging will be performed at baseline and every 2 subsequent cycles (8 weeks). If chemotherapy is postponed due to toxicity, the patient will receive an efficacy evaluation once every 8 weeks.

Evaluation of quality of life: The EORTC QLQ-C30 will be used to assess the quality of life of patients at baseline and before every 2 subsequent cycles.

OS: OS will be calculated from the time of randomization to death due to any cause.

7.2 Safety evaluation

7.2.1 Definition

1. AEs:

An AE refers to any adverse medical event that occurs in clinical subjects and does not necessarily have a causal relation to treatment. Therefore, an AE can be any adverse or unintended physical sign (e.g., abnormal laboratory results), symptom, or transient drug-related disease. In addition, whether an AE is related to the investigational medication will be considered.

AEs that occur before and after treatment are both considered AEs. Therefore,

safety monitoring [reporting of AEs or serious adverse events (SAEs)] will be conducted from the start of the enrolment to the end of the study. AEs that occur after the signing of the ICF until the start of the investigational treatment are also considered AEs.

2. ADR:

All toxic and unintentional dose-dependent reactions to drugs will be considered ADRs. A reaction to a drug indicates that there is a rational causal relationship between the drug and an AE; that is, this relationship cannot be excluded.

3. SAEs:

SAEs refer to all lethal or life-threatening adverse medical events that occur under any drug dose. “Serious” and “life-threatening” are defined as follows: when an AE occurs, the subject is at risk of death, rather that assuming that the AE may lead to death if the AE becomes more serious. If an SAE occurs during the study, the investigators will immediately take appropriate protective measures for the subject and report the SAE to the principal investigator within 24 h. The investigator will complete an SAE report form and sign and date the report.

SAEs include the following AEs:

A. Lethal or life-threatening AEs;

B. AEs that cause hospitalization or prolong hospitalization;

C. AEs that cause permanent disability;

D. Carcinogenic AEs; and

E. Disability-causing AEs.

4. Other events that should be treated as SAEs

Drug exposure during pregnancy/lactation – In principle, patients who are pregnant or lactating will be excluded. If pregnancy occurs during the study period, the patient should immediately withdraw from the study and should inform the investigators immediately; the patient will be followed up throughout the pregnancy and postpartum period. Even if the mother and child are completely healthy and do not have any AEs, observations will be recorded. Even if pregnancy is not an SAE, it will be reported using an SAE report form.

5. Events that should not be treated as SAEs:

Disease progression is generally not considered an SAE (but if the symptoms and

signs of disease progression meet the SAE criteria, it will be reported as an SAE).

Death itself is a consequence and not considered an SAE (the major cause of death, i.e., the major AE leading to death, will be recorded and reported as an SAE, and the death will be reported as the consequence of the corresponding AE; if there is no exact cause of death, then death itself can be reported as an SAE).

The death of a subject within 1 month after the initiation of treatment will be reported as an SAE. If a subject dies 1 month after the initiation of treatment and the death is caused by disease progression, then the death will not be reported as an SAE.

In this study, due to the severity of the disease, some conditions identified as SAEs will be excluded from the immediate report:

A. Optional hospitalization and surgical treatment; and

B. Optional inpatient treatment for the purpose of simplifying treatment or research measures.

7.2.2 Recording and evaluating AEs

Medical terms will be used to describe AEs. All AEs will be recorded in the corresponding section of the CRF. In addition, an SAE report form (including the initial or follow-up report) will be completed. All patients who participate in the study will be included in the summary, and the reasons for withdrawal during the treatment period or exclusion from the summary will be explained. Detailed case reports will be written for deaths and SAEs. For deaths, the cause of death will be ascertained, focusing on the relationship between death and the investigational drug. Follow-up of unresolved AEs will continue until they are properly resolved or the condition of the patient is stable.

The following aspects of each AE will be recorded in the CRF.

1. Occurrence time (start time) and recovery time (end time);

2. Severity, which will be evaluated and graded by the investigators based on the definitions in NCI-CTC version 4.0 (see Appendix 3):

Degree I (mild): discomfort caused by the AE does not interfere with daily activities;

Degree II (moderate): discomfort caused by the AE reduces or interferes with daily activities;

Degree III (severe): discomfort caused by the AE makes patients unable to work or perform daily activities;

Degree IV: life-threatening or disability-causing AE; and

Degree V: death;

3. The possible association between the AE and the investigational drug will evaluated based on a five-level classification scale: definitely related, highly likely related, possibly related, possibly unrelated, and unrelated. The first 3 levels indicate that an AE is related to the investigational drug. The incidence of AEs will be calculated by dividing the total number of subjects with AEs among the first 3 levels with the total number of subjects who participate in the safety evaluation;

Table 7-1 Criteria for determining the relationship between an adverse event and the investigational drug

Circumstances (to be met at the same time)	Definitely related	Highly likely related	Possibly related	Possibly unrelated	Unrelated
A reasonable temporal sequence	Yes	Yes	Yes	Yes	No
A known event	Yes	Yes	Yes	No	No
Improvement after stopping exposure	Yes	Yes	Yes or no	Yes or no	No
Reappearance of the event on re-exposure	Yes	?	?	?	No
Another cause of the event is most plausible	No	No	No	Yes	Yes

Note: "Yes" means affirmative, "No" means dissentient, "Yes or No" means difficult to affirm or deny, and "?" means unclear.

4. Measures taken for the investigational drug (none, suspended treatment, reduced dose, delayed treatment, and slowed intravenous infusion) and other measures (none, concomitant medication, required hospitalization or prolonged hospitalization, underwent surgery, delayed chemotherapy, discontinued chemotherapy, and reduced chemotherapy dose);

5. Consequences are defined as follows: recovery with sequelae, recovery without sequelae, partial recovery with no need for further treatment, partial recovery with the need for further treatment, and death. Whether the change in toxicity grade/severity is serious will be recorded as yes or no. If the patient has the same AE several times, the AE will be recorded and re-evaluated at each occurrence;

6. The criteria for determining whether an abnormal objective examination result should be reported as an AE are as follows:

(1) The examination result is related to the accompanying symptoms (and/or);

(2) Other diagnostic tests or treatment measures/surgical interventions are required based on the examination results (and/or);

(3) The examination results lead to a change in the drug dose or withdrawal from the study, and other concomitant medications or other treatments are required (and/or);

(4) The investigator or the sponsor believes that the examination result should be reported as an AE.

The result of an examination that is only for the purpose of confirming an abnormality but does not meet any of the above criteria does not constitute an AE. Any abnormal examination results that are determined to be wrong are not required to be reported as AEs.

7.2.3 Reporting system for SAEs

The reporting period for SAEs will begin from the signing of informed consent by the subjects until 30 calendar days (including 30 days) after the last dose of the investigational drug. If an SAE occurs, regardless of whether it is the initial report or follow-up report, an SAE Report Form for Clinical Studies must be filled out immediately, signed and dated, and faxed within 24 h of the investigator's knowledge of the event to the Drug Safety Group of the Oncology Business Unit of Hengrui Medicine, the clinical research associate, the principal investigator, the lead institution, the ethics review committee of the clinical research organization, the CFDA, and the investigator's regional (provincial or metropolitan) FDA (the form should be sent to the CFDA via EMS).

SAEs that occur beyond 30 days after the last drug administration will generally not be reported unless they are suspected to be related to the investigational drug.

For SAEs, the symptoms, severity, correlation with the investigational drug, time of occurrence, treatment time, measures taken, time and method of follow-up, and outcome will be recorded in detail. If the investigators believe that an SAE is not related to the investigational drug but is potentially related to the study conditions (such as discontinuation of the original treatment or comorbidities), this relationship will be elaborated on the SAE page of the CRF. If the severity of an ongoing SAE or its relationship with the investigational drug changes, a follow-up report for the SAE will

be sent immediately to the sponsor. All SAEs will be followed up until recovery or SD.

7.2.4 SAE reporting procedures

Any SAE that occurs during the clinical treatment period and within 30 days after drug withdrawal will be reported immediately to the clinical research associate of the sponsor institution and the principal investigator and the ethics review committee of the clinical research organization and will be reported within 24 h of the investigator's knowledge of the event to the Department of Drug and Cosmetics Registration and the Department of Safety Supervision of the CFDA and the health administrative department. The relevant contact information is provided in the table below:

Organization	Contact person	Fax/Telephone/Address
Cancer Hospital of the Chinese Academy of Medical Sciences	Institutional Ethics Committee	Telephone/Fax: 010-87788495
CFDA	Division of Drug Research Supervision, Department of Drug and Cosmetics Registration	Address: Building 2, No.26 Compound, Xuanwumen West Street, Xicheng District, Beijing<br>ZIP Code: 100053<br>Telephone: 010-88330732

8. Data management

8.1 Data entry and modification

Based on the principles of good clinical practice (GCP), the investigators will keep all the detailed original documents of the patients and record relevant content related to study progress, medication status, laboratory test data, safety data and efficacy evaluation in the CRF. The recorded data must be complete, timely and clear. The CRF, original documents, and medical records will be clear, detailed, and easy to identify by the personnel participating in the clinical study.

An independent data management organization will be responsible for data entry

and management. The data administrator will use SPSS 20.0 software for data entry and management. To ensure the accuracy of the data, 2 data administrators will independently perform double entry and proofreading. For problematic content in the CRF, the data administrator will record such issues on the Deep Reasoning Question (DRQ) form and send the form to the investigators through the clinical research associate. The investigator will provide answers as soon as possible, and the data administrator use the responses by the investigator to modify, confirm, and enter the data. If necessary, the DRQ form can be sent again.

8.2 Database security

After data review and verification, the statisticians will lock the database. No modification to the data files will be allowed after locking. Errors found after locking will be verified and corrected through statistical analysis software.

8.3 Data storage

Based on the GCP principles in China, the data will be kept for more than 5 years for the investigators.

9. Statistical analysis

9.1 Sample size determination

On the basis of the previously published data, median progression-free survival with PLD alone was approximately 3·0 months.[3,4] We hypothesized that addition of apatinib could improve the median progression-free survival from 3·0 to 5·5 months. Assuming a power of 80%, a significance level of 0·05 (two-sided), and a dropout rate of 20%, it was calculated by R that a total of 126 patients were required to be enrolled over a period of 24 months and followed up for 4 months, with the primary analysis planned when approximate 86 progression events or deaths had occurred.

9.2 Statistical analysis of datasets

1. Intension-to-treat set (ITT)

All patients randomised will be included in the ITT set. The dataset will be used for the analysis of PFS and OS.

2. Modified Intension-to-treat set (mITT)

All randomised patients who received at least 1 dose of investigational drug and have at least 1 post-baseline tumor assessment will be included in the mITT set. The dataset will be used for the efficacy analysis except for PFS and OS.

3. Safety analysis set

All patients enrolled in the study who receive at least 1 dose of the investigational drug and have a safety record after drug administration will be included in the safety analysis set. This dataset will be used for the safety analysis.

9.3 Statistical analysis methods

The ITT population will be used for efficacy data analysis. The safety population will be used for safety data analysis. Safety set will be used for the safety analysis. The continuous data will be summarized bv using descriptive statistics, including mean, standard deviation, median, maximum, and minimum values. Categorical data will be presented as frequencies and percentages. Progression-free survival and overall survival were estimated using the Kaplan-Meier method, and the corresponding 95% CIs were calculated with Brookmeyer Crowley method. Hazard ratios (HRs) were estimated using a stratified Cox proportional hazards model with corresponding 95% CIs. The stratified log-rank test was used to compare the difference in survival between the two groups. For objective response rate and disease control rate, the 95% CIs were calculated using the Clopper-Pearson method, and compared using the stratified Cochran-Mantel-Haenszel test. The randomization stratification factor, platinum-free interval (≤ 3 months vs. 3–6 months), will be included in all the stratified statistical models and tests as strata. Statistical analyses were conducted using SAS® software (version 9.4, SAS Institute Inc, Cary, USA).

10. Ethical norms and quality control

10.1 Informed consent

It is the responsibility of the investigators to explain in detail the purpose, methods, and benefits and potential risks of the study to each subject and then obtain a written ICF signed by each subject. For subjects who cannot sign the ICF, their legal representative must sign the consent form. If a subject and his/her legal representative do not have the ability to read, a notary public will be present during the entire notification process. After the subject and his/her legal representative consent to participate in the study, the notary public will sign the ICF, proving that the content in the ICF has been accurately explained and understood.

The investigators will also explain to the subjects that they have the full right to deny participation in or exit the study at any time for any reason. The CRF in this study is accompanied by an ICF, which must be completed in full. If new safety information changes the risk/benefit evaluation, the content of the ICF will be modified/updated if necessary. If such a modification/update is made, all subjects (including those who have been treated) will be informed of the new information, and a revised ICF will be provided to the subjects to obtain their consent to continue participating in the study.

10.2 Ethical norms and policies and regulations

This study will be conducted in accordance with GCP guidelines and the latest edition of the Declaration of Helsinki. Approval by the ethics review committee of the Chinese Academy of Medical Sciences will be obtained before the start of this study. During the clinical study, modifications to the protocol will be reported to the ethics review committee and filed for the record. The investigators will report research progress and SAEs to the institutional review board. When the study is complete, the investigators will inform the ethics review committee.

10.3 Quality Assurance

To ensure that this study will be conducted in strict accordance with the clinical research protocol, clinical investigators and clinical sponsors will strictly follow the

GCP requirements during the entire clinical study process, follow the experimental procedures and assure the accuracy of the test data and the reliability of research conclusions.

11. References

1. Nishida N, Yano H, Komai K, Nishida T, Kamura T, Kojiro M. Vascular endothelial growth factor C and vascular endothelial growth factor receptor 2 are related closely to the prognosis of patients with ovarian carcinoma. *Cancer* 2004; **101**(6): 1364-74.

2. Poveda AM, Selle F, Hilpert F, et al. Bevacizumab Combined With Weekly Paclitaxel, Pegylated Liposomal Doxorubicin, or Topotecan in Platinum-Resistant Recurrent Ovarian Cancer: Analysis by Chemotherapy Cohort of the Randomized Phase III AURELIA Trial. *J Clin Oncol* 2015; **33**(32): 3836-8.

3. Naumann RW, Coleman RL, Burger RA, et al. PRECEDENT: a randomized phase II trial comparing vintafolide (EC145) and pegylated liposomal doxorubicin (PLD) in combination versus PLD alone in patients with platinum-resistant ovarian cancer. *J Clin Oncol* 2013; **31**(35): 4400-6.

4. Mutch DG, Orlando M, Goss T, et al. Randomized phase III trial of gemcitabine compared with pegylated liposomal doxorubicin in patients with platinum-resistant ovarian cancer. *J Clin Oncol* 2007; **25**(19): 2811-8.

12. The requirements for biological sample collection from the enrolled patients

12.1 Tissue sections

1. Paraffin sections were generated from tumours obtained during the initial surgery involving enrolled patients; paraffin sections of tumours from patients who underwent their initial surgery in other hospitals will be obtained from those hospitals.

Sectioning requirements: A total of 10 5-μm-thick or 5 10-μm-thick unstained tissue sections, of which nucleated cells compose more than 80%, with the local tumour cell content exceeding 70%, will be stored at room temperature (the paraffin sections

of tumours from patients enrolled in other sites will be sent to the Cancer Hospital of the Chinese Academy of Medical Sciences).

12.2 Blood collection requirements

2. Peripheral blood collection time: Peripheral blood samples (10 mL) will be collected a total of 4 times, namely, before the first, third, and fifth cycles of chemotherapy and one month after the end of the sixth cycle of chemotherapy. If a patient withdraws from the study due to disease progression, another blood sample (10 mL) will be collected. For patients receiving maintenance treatment in the group A, blood samples (10 mL) will be collected during follow-up examinations every 2 months. All blood samples will be collected using 10-ml cfDNA sample storage tubes. After blood samples are collected, the tubes will be inverted 10 times and stored at room temperature (blood samples from the patients enrolled in other sites will be sent to the Cancer Hospital of the Chinese Academy of Medical Sciences within 5 days).

12.3 Technical roadmap

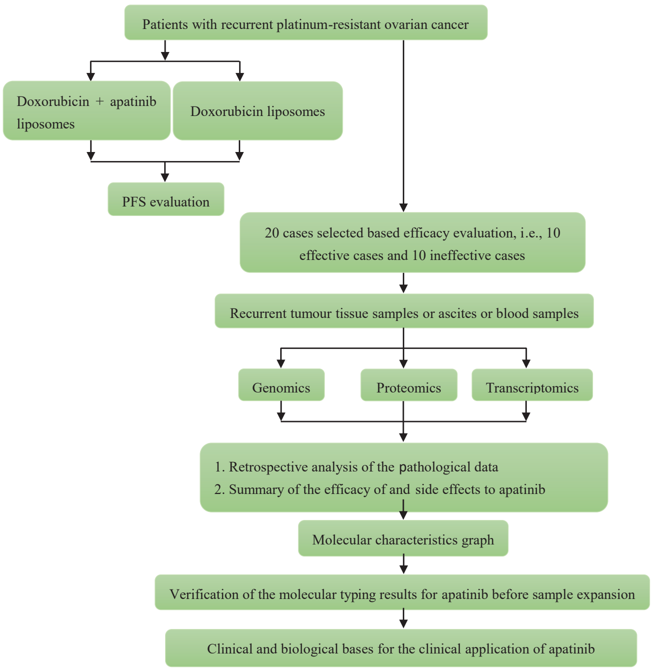

Appendix

Appendix 1. ECOG performance status scoring criteria

Level	Performance status
0	Fully active, capable of all pre-disease performance without restriction
1	Ambulatory and capable of light or sedentary work (light house work or office work) but restricted in physically strenuous activity
2	Ambulatory and capable of all self-care but incapable of any work activities; up and about more than 50% of waking hours
3	Capable of limited self-care; confined to bed or chair more than 50% of waking hours
4	Completely bedridden, incapable of any self-care
5	Dead

Appendix 2. EORTC QLQ-C30 V3.0

We are interested in some things about you and your health. Please answer all of the questions yourself by circling the number that best applies to you. There are no “right” or “wrong” answers. The information that you provide will remain strictly confidential.

Please fill in your code (number):__________

Date of birth:_______year_______month_______day

Today's date: _______year_______month_______day

	Not at all A little Quite a bit Very
1. Do you have trouble performing strenuous activities,	much
Such as carrying a heavy shopping bag or a suitcase?	1 2 3 4
2. Do you have trouble taking a long walk?	
3. Do you have trouble taking a short walk?	1 2 3 4
4. Do you need to stay in bed or a chair during the day?	1 2 3 4
5. Do you need help eating, dressing, washing yourself, or using the	1 2 3 4
toilet?	1 2 3 4
During the past week:	Not at all A little Quite a bit Very
6. Were you limited in performing your work or other daily activities?	much
7. Were you limited in pursuing your hobbies or other leisure time	1 2 3 4
activities?	1 2 3 4
8. Were you short of breath?	1 2 3 4
9. Have you had pain?	1 2 3 4
10. Did you need to rest?	1 2 3 4
11. Have you had trouble sleeping?	1 2 3 4
12. Have you felt weak?	1 2 3 4
13. Have you lacked an appetite?	1 2 3 4
14. Have you felt nauseated?	1 2 3 4
15. Have you vomited?	1 2 3 4
16. Have you been constipated?	1 2 3 4
During the past week:	
17. Have you had diarrhoea?	1 2 3 4
18. Were you tired?	1 2 3 4
19. Did pain interfere with your daily activities?	1 2 3 4
20. Have you had difficulty in concentrating on things, such as reading	1 2 3 4
a newspaper or watching televisions?	1 2 3 4
21. Did you feel tense?	1 2 3 4
22. Did you worry?	1 2 3 4
23. Did you feel irritable?	1 2 3 4
24. Did you feel depressed?	1 2 3 4
25. Have you had difficulty remembering things?	1 2 3 4
26. Has your physical condition or medical treatment interfered with	1 2 3 4
your family life?	1 2 3 4
27. Has your physical condition or treatment interfered with your	1 2 3 4

social activities?

28. Has your physical condition or treatment caused you financial difficulties? 1 2 3 4

For the following questions, please circle the number between 1 and 7 that best applies to you.

29. How would you rate your overall health in the past week?

1 2 3 4 5 6 7

Very poor Very good

30. How would you rate your overall quality of life in the past week?

1 2 3 4 5 6 7

Very poor Very good

Note:

1. Description of quality of life rating

The EORTC QLQ-C30 (V3.0) is a core scale for all cancer patients, with a total of 30 items. Items 29 and 30 are scored on a 1 to 7 scale. The other items are scored on 1 to 4 scale: not at all, a little, quite a bit, and very much, respectively.

2. Calculation of the EORTC QLQ-C30 domain (dimension) scores [raw score (RS)]

For the statistical analysis and interpretation, the scale is often divided into domains. Each domain is a component of quality of life. A domain is also known as a dimension. It is an independent variable in analyses. The 30 items on the EORTC QLQ-C30 (V3.0) are categorised into 15 domains, including 5 function domains (physical, role, cognitive, emotional, and social), 3 symptom domains (fatigue, pain, and nausea/vomiting), 1 general health status/quality of life domain, and 6 single items (each as a domain). The classification is shown in the table below.

The score (RS) for a domain can be obtained by dividing the sum of the scores of all items included in the domain by the total number of items included in the domain, i.e., RS = (Q1+Q2+…+Qn)/n.

EORTC's QLQ-C30 domain classification

	Entry	Item number
Physical functioning	5	1-5
Role functioning	2	6-7
Emotional functioning	4	21-24
Cognitive functioning	2	20-25
Social functioning	2	26-27
General health status	2	29-30
Fatigue	3	10, 12, 18
Nausea/vomiting	2	14-15
Pain	2	9, 19
Shortness of breath	1	8
Insomnia	1	11
Loss of appetite	1	13
Constipation	1	16
Diarrhoea	1	17

Financial difficulties	1	28

3. Calculation of the EORTC QLQ-C30 standard scores

To compare the scores for each domain, the RS must be converted by linear transformation into the standard score (SS) with values ranging from 0-100. In addition, the transformation has another purpose, namely, changing the scoring direction. The items on the QLQ-C30 scale, except for items 29 and 30, are all reverse-scored items (the higher the score, the worse the quality of life), and the scoring rules clearly stipulate that higher scores for function domains and the general health status domain indicate better a functioning status and quality of life and that higher scores for the symptom domains indicate more symptoms or problems (worse quality of life). Therefore, the scoring direction must be changed in the calculation of the SS for the function domains. Specifically, the following equations are used for the calculation (where R is the full range of scores for each domain or item).

Function domains: SS=[1-(RS-1)/R]*100

Symptom domains and general health status domain: SS=[(RS-1)/R]*100

Appendix 3. Response Evaluation Criteria in Solid Tumours (RECIST 1.1) criteria

1 The measurability of the tumour at the baseline level

1.1 Definition:

At the baseline level, tumours/lymph nodes are classified as measurable and unmeasurable based on the following definitions.

1.1.1 Measurable lesions

Tumours: For measurable lesions, there must be at least 1 diameter that can be accurately measured (recorded as the longest diameter). The longest diameter should be **≥ 10 mm on CT (recommended CT slice thickness is ≤ 5 mm),** ≥10 mm on clinical examination using conventional instruments (tumours that cannot be accurately measured by conventional calliper devices will be recorded as unmeasurable), and ≥ 20 mm on chest X-ray. **Malignant lymph nodes are pathologically enlarged and measurable, and the short-axis diameter (SAD) of a single lymph node should be ≥ 15 mm on CT (recommended CT slice thickness is ≤ 5 mm).** At baseline and follow-up, only the SAD will be measured and followed up.

1.1.2 Unmeasurable lesions

All other lesions include small lesions (longest diameter <10 mm or pathological lymph node SAD ≥ 10 mm to < 15 mm) and unmeasurable lesions. Unmeasurable lesions include meningeal disease, ascites, pleural or pericardial effusion, inflammatory breast cancer, cutaneous/pulmonary lymphangitis carcinomatosis, abdominal mass that cannot be diagnosed and followed up by imaging, and cystic lesions.

1.1.3 Special concerns for lesion measurement

Bone lesions, cystic lesions, and lesions that have received local treatment will be noted:

➢ Bone lesions:

- Bone scans, positron emission tomography (PET) scans or plain films are not suitable for measuring bone lesions but will be used to confirm the presence or disappearance of bone lesions.

- If osteolytic lesions or mixed osteolytic/osteogenic lesions have a definite soft tissue composition that meets the above definition of measurability and are evaluable using tomographic imaging techniques such as CT or MRI, then these lesions will be considered measurable.
- Osteogenic lesions are unmeasurable lesions.

➢ Cystic lesions:

- Lesions that meet the definition criteria for simple cysts on radiography will not be considered malignant just because they are simple cysts by definition. They are neither measurable lesions nor unmeasurable lesions.
- If a cystic metastatic lesion meets the definition criteria for measurability, it can be regarded as measurable. However, if cystic and noncystic lesions simultaneously present in the same patient, noncystic lesions will be preferentially selected as target lesions.

Locally treated lesions:

- Lesions located at sites that have received radiotherapy or other local treatments will generally be regarded as unmeasurable lesions unless there is clear progression of the lesion. The study protocol will elaborate the conditions under which these lesions are measurable.

1.2 Measurement method description

1.2.1 Measurement of lesions

During clinical evaluations, all tumour measurements will be recorded in the record system. All baseline evaluations of tumour size will be completed before and as close as possible to the start of treatment and **must be completed within 28 days (4 weeks) before the start of treatment.**

1.2.2 Evaluation methods

The same techniques and methods will be used for baseline evaluations and subsequent lesion measurements. Except for lesions that cannot be evaluated by imaging but can only be evaluated by clinical examination, all lesions will be evaluated by imaging.

Clinical lesions: Clinical lesions will only be considered measurable when they are

superficial and have a diameter ≥ 10 mm at the time of measurement (such as skin nodules). For patients with skin lesions, it is recommended to archive colour photographs containing scales to measure lesion sizes. When the lesions are evaluable using both imaging and clinical examinations, imaging will be selected whenever possible because imaging is more objective and can be reviewed repeatedly after the end of the study.

Chest X-ray: When tumour progression is set as a primary endpoint, chest CT is preferred because CT is more sensitive than X-ray, especially for new lesions. Chest X-ray is only applicable when the measured lesion has a clear boundary and the lungs are well ventilated.

CT and MRI: CT is currently the best reproducible method for efficacy evaluations. The measurability in this guideline is defined based on a CT slice thickness ≤ 5 mm. If the CT slice thickness is >5 mm, the minimum measurable lesion will be 2 times the slice thickness. MRI is also acceptable in some cases (e.g., whole-body scan).

Ultrasound: Ultrasound will not be used to measure lesion size. Due to its operational dependence, ultrasound is not repeatable and technical and operational homogeneity cannot be guaranteed among different measurements. New lesions detected by ultrasound during the treatment period will be confirmed by CT or MRI. If CT radiation exposure is a concern, MRI will be used instead.

Endoscopy and laparoscopy: These techniques are not recommended for the objective evaluation of tumours. However, they will be used to obtain biopsy specimens for CR confirmation or for confirmation of recurrence after CR or in surgical resection.

Tumour markers: Tumour markers alone will not be used to evaluate objective tumour response. However, if the baseline level of a tumour marker exceeds the ULN, it will not be used to evaluate CR unless its level returns to normal. The variation in tumour markers in different diseases needs to be taken into account when formulating the measurement criteria into the protocol. Specific criteria for CA-125 (recurrent ovarian cancer) and prostate-specific antigen (recurrent prostate cancer) responses have been published. In addition, the Gynecologic Cancer Intergroup (GCIG) has developed CA-125 progression criteria, which will soon be added to the objective tumour

evaluation criteria of the first-line treatment regimens for ovarian cancer.

Cytological/histological techniques: Under conditions specified in the protocol, these techniques can be used to identify PR and CR (such as the presence of residual benign tumour tissue in germ cell tumours). When exudation may be a potential side effect of a certain therapy (such as treatment with taxane compounds or angiogenesis inhibitors) and a measurable tumour meets the criteria for response or SD, the occurrence or exacerbation of tumour-related exudation during the treatment will be diagnosed by cytological techniques to distinguish between response (or SD) and disease progression.

2 Tumour response evaluation

2.1 Evaluation of all tumours and measurable lesions

To evaluate objective response or possible future progression, it will be necessary to perform a baseline evaluation of the total tumour burden as a reference for the subsequent measurements. In the clinical protocols with objective response as the primary endpoint, only patients with measurable lesions at baseline are eligible. Measurable lesions are defined as the presence of at least one measurable lesion. For trials with disease progression (disease progression time or degree of progression on a fixed date) as the primary endpoint, the inclusion criteria for the protocols will clarify whether only patients with measurable lesions are eligible or patients with no measurable lesions are also eligible.

2.2 Baseline records of target lesions and nontarget lesions

When there is more than 1 measurable lesion during a baseline evaluation, all measurable lesions will be recorded and measured, but the **total number of measurable lesions will not exceed 5 (no more than 2 in each organ)**. For patients with 1 or 2 organs involved, up to 2 or 4 target lesions will be selected for baseline measurements. The target lesions will be selected based on the size (the longest diameter) and will represent all the involved organs, and the measurement will be highly reproducible. If the measurement of the largest lesion is not reproducible, the largest one among reproducible lesions will be selected.

Special attention will be paid to lymph nodes because they are normal tissues and can be detected by imaging even without tumour metastasis. Pathological lymph nodes defined as measurable nodules or even target lesions must meet the following criteria. The CT measurement of the SAD must be ≥ 15 mm. Only the SAD will be measured at baseline. Radiologists usually use the SAD of a nodule to determine whether the nodule has undergone metastasis. The size of a nodule is generally represented by the two-dimensional data from imaging (the axial plane on CT and the axial, sagittal or coronal plane on MRI). The minimum value is the SAD. For example, a 20 mm × 30 mm abdominal nodule has an SAD of 20 mm and can be regarded as malignant and measurable. In this example, 20 mm is the measured value of the nodule. Nodules with a diameter ≥ 10 mm but < 15 mm will not be considered target lesions. Nodules < 10 mm will not be categorized as pathological nodules and will not be recorded and further observed.

The sum of the diameters of all target lesions (including the longest diameter of nonnodular lesions and the SAD of nodular lesions) will be reported as the baseline sum of the diameters. If the diameters of lymph nodes are included, as mentioned above, only the SAD is included. The baseline sum of the diameters will be used as the reference value for the baseline level of the disease.

All other lesions, including pathological lymph nodes, will be considered nontarget lesions and will not need be measured but will be recorded during the baseline evaluation. For example, the lesion will be classified as "existing", "missing" or, in rare cases, "clear progression". Extensive target lesions will be recorded with target organs (e.g., numerous enlarged pelvic lymph nodes or large-scale liver metastasis).

2.3 Criteria for response

2.3.1 Evaluation of target lesions

CR: All target lesions have disappeared, and the SADs of all pathological lymph nodes (including target nodules and nontarget nodules) have reduced to <10 mm.

PR: The sum of the diameters of all target lesions is at least 30% higher than the diameter sum at baseline.

PD: The sum of the diameters of all target lesions is at least 20% higher than the

reference diameter sum, which is the minimum value of the sum of the diameters of all target lesions measured during the entire study period (if the diameter sum at baseline is the smallest, the baseline value is used as the reference). In addition, the absolute value of the diameter sum must have increased by at least 5 mm (**the appearance of 1 or more new lesions is also considered PD**).

SD: SD is a status between PR and PD, where the reduction in the target lesion does not reach PR and the increase in the target lesion does not reach PD. The minimum value of the diameter sum will be used as a reference.

2.3.2 Precautions for target lesion evaluations

Lymph nodes: Even if the size of the lymph nodes identified as target lesions decreases to less than 10 mm, the actual SAD corresponding to the baseline will be recorded for each measurement (consistent with the anatomical plane of the baseline measurement). This means that if a lymph node is the target lesion, even if CR is achieved, it cannot be concluded that the lesion has disappeared completely because the SAD of a normal lymph node is defined as <10 mm. In the CRF or other recording forms, it is necessary to specifically record the target lesion (lymph node) at a specific location: for CR, the SADs of all lymph nodes must be <10 mm; for PR, SD and PD, the measured value of the SAD of the target lymph node will be included in the sum of the diameters of the target lesions.

Target lesions that are too small to be measured: In clinical studies, all lesions (nodules or non-nodules) recorded at baseline should be measured again and recorded, even if the lesions are very small (e.g., 2 mm). However, sometimes a lesion may be too small, resulting in very fuzzy CT images. In this case, the radiologists cannot accurately define the exact size of the lesion and may report it as "too small to be measured". However, it is very important to record a value on the CRF in this condition. In this trial, even if the radiologist believes that the lesion may have disappeared, he/she will still record 0 mm on the CRF. If the lesion does exist but is too fuzzy to be accurately measured, the default size will be 5 mm (note: this condition is unlikely to occur in lymph nodes because they generally have a measurable size under normal conditions or are often surrounded by adipose tissue as in the retroperitoneal cavity,

whereas if the lymph nodes cannot be measured accurately, the default size will also be 5 mm). The default size of 5 mm is derived from the slice thickness of the CT scan (however, this value does not change with the slice thickness of the CT scan). Because the probability of the repeated occurrence of the same measurement value is small, the use of this default value will reduce the risk of an erroneous evaluation. However, it should be reiterated that if the radiologist can obtain the exact lesion size, even if the diameter of the lesion is less than 5 mm, the actual size will be recorded.

Dissociated or combined lesions: When a nonnodular lesion dissociates into fragments, the sum of the diameters of the fragments will be calculated by adding the longest diameters of all fragments. Similarly, the planes between combined lesions can be used to distinguish each lesion, and then the longest diameter of each lesion can be calculated. However, if the combined lesions fuse into an inseparable lesion, the longest diameter should be the longest diameter of the entire fused lesion.

2.3.3 Evaluation of nontarget lesions

This section defines the response criteria for nontarget tumours. Although some nontarget lesions are measurable, they will not be measured but will be qualitatively evaluated at the time points planned in the protocol.

CR: All nontarget lesions have disappeared, and tumour markers have returned to normal levels; all lymph nodes are of nonpathological size (SAD $\leq$ 10 mm).

PR/non-PD: The presence of one or more nontarget lesions and/or the persistent presence of abnormally high tumour marker levels.

PD: Clear progression of existing nontarget lesions. **Note: The appearance of one or more new lesions is also considered PD**.

2.3.4 Special considerations for rating the progression of nontarget lesions

The following is a supplementary explanation of the definition of the progression of nontarget lesions: when patients have measurable nontarget lesions, to achieve ‘unequivocal progression’ on the basis of the non-target disease, there must be an overall level of substantial worsening in non-target disease such that, even in presence of SD or PR in target disease, the overall tumour burden has increased sufficiently to merit discontinuation of therapy. However, a general size increase in 1 or more

nontarget lesions often cannot meet the criteria for progression. The designation of overall progression solely on the basis of change in non-target disease in the face of SD or PR of target disease will therefore be extremely rare..

The situation that all nontarget lesions are unmeasurable may occur in some phase III trials, in which the inclusion criteria do not stipulate the necessity of measurable lesions. The overall evaluation is based on the above criteria, but measurable lesion data are absent in this situation. Because worsening in non-target disease cannot be easily quantified (by definition: if all lesions are truly non-measurable) a useful test that can be applied when assessing patients for unequivocal progression is to consider if the increase in overall disease burden based on the change in non-measurable disease is comparable in magnitude to the increase that would be required to declare PD for measurable disease. For example, such progression can be described as an increase in tumour burden equivalent to an additional 73% increase in volume (equivalent to a 20% increase in the diameters of measurable lesions), the change in peritoneal exudation from “minimal” to “massive”, the change in lymphatic lesions from “local” to “extensively expanded”, or “sufficient to change the treatment method” in the protocol. Examples include pleural exudate increasing from a trace amount to a large amount, lymphatic involvement spreading distally from the primary site, or a description of "necessity for treatment changes" in the protocol. In this clinical study, if there is clear progression, the patient will be considered to have disease progression at that time point. It is best to have objective criteria that can be applied to the evaluation of unmeasurable lesions. Note that the supplementary criteria must be reliable.

2.3.5 New lesions

The appearance of new malignant lesions indicates disease progression; therefore, the evaluation of new lesions is critical. Currently, there is no specific standard for the imaging detection of lesions. However, the finding of a new lesion should be unequivocal. For example, progression cannot be attributed to different imaging techniques, changes in imaging morphology, or lesions other than tumours (e.g., some so-called new bone lesions are merely cured original lesions or the recurrence of original lesions). This is particularly important when the patient’s baseline lesions show

partial or complete response. For example, a necrotized liver lesion may be classified as a new cystic lesion on the CT report, although it is not.

Lesions that are detected during the follow-up but were not found in the baseline examination will be considered new lesions and suggestive of disease progression. For example, if a patient with visceral lesions in the baseline examination is found to have metastases during a CT or MRI cranial examination, intracranial metastases will be considered the basis of disease progression even if the patient did not undergo a cranial examination during the baseline examination. If a new lesion is equivocal, for example because of its small size, continued therapy and follow-up evaluation will clarify if it represents truly new disease. If repeat scans confirm there is definitely a new lesion, then progression should be declared using the date of the initial scan.

In addition to fluorodeoxyglucose (FDG)-PET, additional tests are generally required for supplementary confirmation. It is rational to combine FDG-PET and complementary CT results to evaluate progression (especially for new suspected diseases). New lesions can be confirmed by FDG-PET using the following procedures.

Negative baseline FDG-PET results together with the positive FDG-PET results in a subsequent follow-up indicate disease progression.

In cases with no baseline FDG-PET and positive FDG-PET results in a subsequent follow-up, if the new lesion indicated by the positive FDG-PET is confirmed by CT, then disease progression is confirmed; if the new lesion indicated by the positive results of the follow-up FDG-PET is not confirmed by CT, another CT should be performed to confirm the lesion (if confirmed, the disease progression time is counted from the initial discovery of the lesion); if the lesion is confirmed to be an existing lesion recorded in a past CT examination and no progression of the lesion has been found by imaging, then there is no disease progression.

2.4 Evaluation of the best overall efficacy

The best overall efficacy will be evaluated based on the best response from the start of treatment to the end of treatment, and all requisites will be considered for confirmation. Sometimes, the response occurs after the end of treatment. Therefore, the protocol will clarify whether an efficacy evaluation after the end of the treatment is

included in the best overall efficacy evaluation. The protocol will clarify how any new treatment before progression affects the best response. The best response mainly depends on target lesion and nontarget lesion results, as well as the presence of new lesions. In addition, depending on the nature of the study and the protocol requirements, it may also require confirmatory measurement. Specifically, in nonrandomized trials, response is the primary goal, and efficacy, i.e., PR or CR, must be confirmed to determine the best overall efficacy.

2.4.1 Time point response

It is assumed that at each protocol specified time point, a response assessment occurs. Table 1 summarises the overall response of a patient population with measurable disease at baseline at each time point. The evaluation of patients with no measurable lesions (no target lesions) is detailed in Table 2.

2.4.2 Missing assessments and inevaluable designation

When no imaging/measurement is done at all at a particular time point, the patient is not evaluable (NE) at that time point. If only a subset of lesion measurements are made at an assessment, usually the case is also considered NE at that time point, unless a convincing argument can be made that the contribution of the individual missing lesion(s) would not change the assigned time point response. This would be most likely to happen in the case of PD. For example, if a patient had a baseline sum of 50 mm with three measured lesions and at follow-up only two lesions were assessed, but those gave a sum of 80 mm, the patient will have achieved PD status, regardless of the contribution of the missing lesion.

2.4.3 Best overall response

At all time points, once all the patient data are available, the best overall response will be determined.

Evaluation of the best overall response when confirmation of CR or PR is not required: The best treatment response is the best response at all time points (e.g., if the efficacy in a patient is rated SD for the first cycle, PR for the second cycle, and PD for the last cycle, then the best overall response for the patient is PR). When the best overall response is rated SD, the requirement for the minimum interval from baseline to SD

specified in the protocol must be met. If the minimum interval requirement is not met, even if the best overall response is rated SD, the evaluation result will not recognized, and the best overall response of the patient will be determined by a subsequent evaluation. For example, if a patient response is rated SD for the first cycle and PD for the second cycle but does not meet the minimum interval requirement, the best overall response is PD. Similarly, if a patient response is rated SD for the first cycle but the patient becomes lost to follow-up after the first cycle, the best overall response is considered NE.

Evaluation of the best overall response when confirmation of CR or PR is required: If each subject meets the criteria for PR or CR and the efficacy is confirmed at a subsequent time point (usually 4 weeks later) specifically mentioned in the protocol, the best overall response can be declared CR or PR. In this situation, the best overall response is detailed in Table 3.

2.4.4 Special tips for efficacy evaluations

When nodular lesions are included in the total target lesion evaluation and the size of the nodule decreases to the "normal" size (<10 mm), a lesion size scan report will be generated. To avoid the over-evaluation of efficacy based on an increase in nodule size, the measurement results will be recorded even if the nodule is normal. As mentioned earlier, this means that even for subjects with a CR, the nodule size will not be recorded as 0 on the CRF. If the efficacy needs to be confirmed during treatment, repeated "unmeasurable" time points will complicate the evaluation of the best efficacy. The analysis plan must state that these missing data/evaluations will be clearly explained when determining efficacy. For example, in most trials, PR-NE-PR can be regarded as confirmation of efficacy.

When treatment must be discontinued due to the deterioration in the general health status of a participant that is not supported by objective evidence, the efficacy will be reported as symptomatic progression. Even after the discontinuation of treatment, objective progression will be evaluated whenever possible. Symptomatic deterioration is a reason for the discontinuation of treatment but is not an objective response evaluation. The objective response of such subjects will be evaluated based on the

conditions of target and nontarget lesions, as detailed in Tables 1 to 3.

Conditions defined as early progression, early death and NE are special cases and will be clearly described for each regimen (depending on the treatment interval and treatment cycle).

In some circumstances it may be difficult to distinguish residual disease from normal tissue. When the evaluation of complete response depends upon this determination, it is recommended that the residual lesion be investigated (fine needle aspirate/biopsy) before assigning a status of complete response. If abnormal imaging results for local lesions are considered to represent lesion fibrosis or scar formation, FDG-PET can be used as an evaluation standard, similar to biopsy, to confirm CR. In this situation, the application of FDG-PET will be prospectively described in the protocol, and the specialized medical literature reporting this situation will be cited as support. However, limitations of FDG-PET and biopsy (including their resolution and sensitivity) will lead to false positive results in the evaluation of CR.

Table 1 Time point response: patients with target (+/–non-target) disease

Target lesions	Nontarget lesions	New lesions	Overall response
CR	CR	No	CR
CR	non-CR/non-PD	No	PR
CR	Not fully evaluable	No	PR
PR	non-PD or not fully evaluable	No	PR
SD	non-PD or not fully evaluable	No	SD
Not fully evaluable	non-PD	No	NE
PD	Any	Yes or no	PD
Any	PD	Yes or no	PD
Any	Any	Yes	PD
CR = complete response; PR = partial response; SD = stable disease; PD = progressive disease; NE =non-evaluable			

Table 2 Time point response: patients with non-target disease only

Nontarget lesions	New lesions	overall response
CR	No	CR
Non-CR or non-PD	No	Non-CR or non-PD
Not fully evaluable	No	NE

Unconfirmed PD	Yes or no	PD
Any	Yes	PD

Note: For nontarget lesions, "non-CR/non-PD" refers to an efficacy superior to SD. As SD is increasingly used as an endpoint for rating efficacy, non-CR/non-PD has been formulated to target conditions without measurable lesions. For unclear progression findings (such as very small unconfirmed new lesions and cystic degeneration of original lesions or necrotized original lesions), treatment will be continued until the next evaluation. If disease progression is confirmed in the next evaluation, the date of progression will be the date when progression is first suspected.

Table 3 Confirmation of best overall response as CR or PR

Overall response at the first time point	Overall response at the subsequent time point	Best overall response
CR	CR	CR
CR	PR	SD, PD or PR
CR	SD	If the minimum interval requirement for SD is satisfied, then the best overall response is SD; otherwise, it is PD.
CR	PD	If the minimum interval requirement for SD is satisfied, then the best overall response is SD; otherwise, it is PD.
CR	NE	If the minimum interval requirement for SD is satisfied, then the best overall response is SD; otherwise, it is NE.
PR	CR	PR
PR	PR	PR
PR	SD	SD
PR	PD	If the minimum interval requirement for SD is satisfied, then the best overall response is SD; otherwise, it is PD.
PR	NE	If the minimum interval requirement for SD is satisfied, then the best overall response is SD; otherwise, it is NE.
NE	NE	NE

Note: CR is complete response, PR is partial response, SD is stable disease, PD is progressive disease, and NE is non-evaluable. Superscript "a": If CR truly occurs at the first time point and any disease occurs at the subsequent time point, then treatment efficacy at the subsequent time point is still rated PD (because the disease will occur after a CR) even if the efficacy meets the PR criteria relative to baseline. The best response depends on whether SD occurs within the minimum treatment interval. Sometimes, although the evaluation at the first time point is CR, scans at subsequent

time points suggest small lesions; therefore, the treatment efficacy for the subject at the first time point should be PR rather than CR. In this situation, the first judgement of CR should be modified to PR, and the best response is PR.

2.5 Frequency of tumour re-evaluations

The frequency of tumour re-evaluations during treatment will be determined by the treatment regimens and will occur in accordance with the type of and schedule for treatment. However, in the phase II trial in which the benefit of treatment was not clear, it is reasonable to conduct follow-up every 6 to 8 weeks (at the end of a cycle), and the length of the time interval will be adjusted under special schemes or circumstances. The protocol will specifically indicate which tissue sites need to be evaluated at baseline (usually those tissue sites that are most likely to be closely related to the metastatic lesions of the tumour type under study) and the frequency of re-evaluation. Under normal circumstances, target lesions and nontarget lesions will be rated at each evaluation. In some optional cases, the evaluation frequency of some nontarget lesions will be lower; for example, bone scans should be repeated only when the treatment efficacy for the target disease is confirmed to be CR or when bone lesion progression is suspected.

After the end of treatment, the re-evaluation of tumours depends on whether the response rate or the occurrence of a certain event (progression/death) is used as the clinical trial endpoint. If the clinical trial endpoint is the occurrence of a certain event (e.g., TTP/DFS 1/PFS), routine re-evaluations, as specified in the protocol, are required. In randomized comparative trials, the scheduled evaluations should be listed in the study schedule (e.g., 6-8 weeks of treatment or 3-4 months after the treatment) and should not be affected by other factors, such as treatment delays, dosing intervals, and any other events that may cause an imbalance in the treatment arm regarding the selection of disease evaluation time.

2.6 Efficacy evaluation/response confirmation

2.6.1 Confirmation

For nonrandomized clinical trials with efficacy as the primary endpoint, PR and

CR must be confirmed to ensure that the efficacy is not the result of an erroneous evaluation. Confirmation also allows a reasonable interpretation of the results in the presence of historical data, and efficacy in the historical data of these trials should also be confirmed. However, in all other cases, such as randomized trials (Phase II or III) or studies with SD or PD as the primary endpoint, efficacy confirmation is no longer necessary because it is of no value for the interpretation of the experimental results. However, elimination of the requirement for response confirmation may increase the importance of central review to protect against bias, in particular in studies which are not blinded. In the case of SD, at least 1 measurement must meet the SD criteria specified in the protocol within the minimum time interval after the start of treatment (generally no less than 6-8 weeks).

2.6.2 Total response period

The total response period is the interval from the time when the CR or PR (whichever is measured first) criteria are first met to the time of the first actual recording of disease recurrence or progression (the minimum measured value recorded during treatment is used as the reference for disease progression). The total CR period is the interval from the time when the CR criteria are first met to the time of the first actual recording of disease recurrence or progression.

2.6.3 SD period

The SD period is the interval from the start of treatment to disease progression (in a randomized trial, from the time of randomization), and the smallest sum during treatment is used as the reference (if the baseline sum is the smallest, it is used as the reference for PD calculations). The clinical relevance of SD varies in different studies and different diseases. If the proportion of patients achieving stable disease for a minimum period of time is an endpoint of importance in a particular trial, the protocol should specify the minimal time interval required between two measurements for determination of stable disease. Note: The response period, SD period, and PFS are affected by the follow-up frequency after the baseline evaluation. Defining the standard follow-up frequency is outside the scope of this guideline. The frequency of follow-up should consider many factors, such as disease type and stage, treatment cycle and

standard specifications. However, if intertrial comparisons are required, the limitations of the accuracy of these measurement endpoints should be considered.

Appendix 4. 2014 FIGO staging for ovarian cancer, fallopian tube cancer, and peritoneal cancer

I		Tumour confined to the ovaries or fallopian tube(s)
	IA	Tumour limited to 1 ovary (capsule intact) or fallopian tube, no tumour on the ovarian or fallopian tube surface, and no malignant cells in the ascites or peritoneal washings
	IB	Tumour limited to both ovaries (capsule intact) or fallopian tubes, no tumour on the ovarian or fallopian tube surface, and no malignant cells in the ascites or peritoneal washings
	IC	Tumour limited to one or both ovaries or fallopian tubes, with any of the following:<br>IC1 Surgical spill intraoperatively;<br>IC2 Capsule ruptured before surgery or tumour on the ovarian or fallopian tube surface; or<br>IC3 Malignant cells in the ascites or peritoneal washings
II		Tumour involves 1 or both ovaries or fallopian tubes with pelvic extension (below the pelvic brim) or peritoneal cancer (Tp)
	IIA	Extension and/or implants on the uterus and/or fallopian tubes and/or ovaries
	IIB	Extension to other pelvic intraperitoneal tissues
III		Tumour involves 1 or both ovaries or fallopian tubes or primary peritoneal cancer with cytologically or histologically confirmed spread to the peritoneum outside the pelvis and (or) metastasis to the retroperitoneal lymph nodes
	IIIA	Metastasis to retroperitoneal lymph nodes with or without microscopic peritoneal involvement beyond the pelvis<br>IIIA1 Positive retroperitoneal lymph node only (cytologically or histologically confirmed)<br>IIIA1(i) Metastasis ≤ 10 mm in greatest dimension (note: this is the tumour dimension and not the lymph node dimension)<br>IIIA1(ii) Metastasis >10 mm in the greatest dimension<br>IIIA2 Microscopic extrapelvic (above the pelvic brim), peritoneal involvement with or without positive retroperitoneal lymph nodes
	IIIB	Macroscopic peritoneal metastasis beyond the pelvic brim ≤ 2 cm in greatest dimension, with or without positive retroperitoneal lymph nodes
	IIIC	Macroscopic peritoneal metastasis beyond the pelvic brim > 2 cm in greatest dimension, with or without positive retroperitoneal lymph nodes (Note 1)

IV		Distant metastasis excluding peritoneal metastasis
	IVA	Pleural effusion with positive cytology
	IVB	Metastasis to extra-abdominal organs (including inguinal lymph nodes and lymph nodes outside of abdominal cavity) (Note 2)

Note 1: Includes extension of the tumour to the capsule of the liver and spleen without parenchymal involvement of either organ.
Note 2: Parenchymal metastases are stage IVB.

Appendix 5. New York Heart Association (NYHA) classification

Grade I	No limitation in physical activity; ordinary physical activity does not cause undue fatigue, dyspnoea, or palpitations, namely, cardiac compensation
Grade II	Slight limitation in physical activity; ordinary physical activity causes fatigue, dyspnoea, palpitations, or angina, also known as grade I or mild heart failure
Grade III	Marked limitation in physical activity; less than ordinary physical activity leads to the above symptoms, also known as grade II or moderate heart failure
Grade IV	Unable to carry out any physical activity; symptoms of congestive heart failure or angina occur at rest and aggravate after any physical activity, also known as grade III or severe heart failure

Apatinib Combined with Standard Chemotherapy for Platinum-Resistant Recurrent Ovarian Cancer: The APPROVE Study

Study protocol

Version 3.0

Version date: January 31, 2020

**Project:**	**Optimization of treatment regimens and clinical pathways for ovarian cancer**
**Programme:**	**Research on the prevention and control of major chronic non-communicable diseases**
**Lead Site:**	**Cancer Hospital, Chinese Academy of Medical Sciences**
**Research Site:**	**Cancer Hospital, Chinese Academy of Medical Sciences**
**Principal investigator:**	**Lingying Wu**
**Implementation period:**	**September 2016-December 2020**

Collaborating institutions and contacts

Serial number	Institution name	Responsible person/Contact
1	Cancer Hospital, Chinese Academy of Medical Sciences	Lingying Wu/Ning Li
2	Peking Union Medical College Hospital	Lingya Pan/Yu Gu
3	West China Second University Hospital of Sichuan University	Rutie Yin/Qingli Li/Xinxin Jiang
4	Chongqing University Cancer Hospital	Dongling Zou
5	Peking University Cancer Hospital	Hong Zheng/Qian Li
6	Fudan University Shanghai Cancer Center	Xiaojun Chen/Wei Zhang
7	Hunan Cancer Hospital	Jie Tang/Ying Wang
8	The Second Affiliated Hospital of Dalian Medical University	Kui Jiang
9	The First Hospital of Jilin University	Ziling Liu/Zhi Li
10	Xiangya Hospital of Central South University	Lanqin Cao
11	Liaoning Cancer Hospital & Institute	Jingru Zhang
12	Beijing Obstetrics and Gynecology Hospital, Capital Medical University	Jinwei Miao/Di Wu
13	Guangxi Medical University Affiliated Tumor Hospital	Li Li/Bingbing Zhao
14	Hubei Cancer Hospital	Yi Huang
15	Yunnan Cancer Hospital	Hongying Yang/Yi Zhang
16	Tianjin Medical University Cancer Institute & Hospital	Ke Wang/Li Zhang
17	Shandong Cancer Hospital	Qingshui Li

Table of contents

Synopsis......5
1. **Background**......11
    1.1 Progress in antiangiogenic therapy for recurrent platinum-resistant ovarian cancer 11
    1.2 Drug name......14
    1.3 Results of preclinical studies of apatinib......14
    1.4 Results of the phase I clinical trial of apatinib 17
2. **Study purpose**......22
    2.1 Primary outcome measures 22
    2.2 Secondary outcome measures 22
3. **Study design**......23
4. **Subjects**......23
    4.1 Inclusion criteria 24
    4.2 Exclusion criteria 24
    4.3 Criteria for termination or withdrawal 26
    4.4 Relevant post-withdrawal regulations for patients in the monochemotherapy group 27
    4.5 Early termination of the research project 27
5. **Study schedule and evaluation**......27
    5.1 Screening examination and baseline evaluation (will be completed within 21 days before the start of treatment) 28
    5.2 Evaluation of the treatment period......28
    5.3 End-of-study evaluation......29
    5.4 Follow-up......30
    5.5 Evaluation of early exit and withdrawal 30
    5.6 Study duration......30
    5.7 Study schedule 31
6. **Investigational drug**......34
    6.1 Treatment regimens......34
    6.2 Dose adjustment......34
    6.3 Concomitant therapy......43
    6.4 Drug policy 44
7. Study endpoint evaluation......44
    7.1 Efficacy evaluation......44
    7.2 Safety evaluation......44
8. Data management 49
    8.1 Data entry and modification......49
    8.2 Database security 50
    8.3 Data storage......50
9. Statistical analysis 50
    9.1 Sample size determination 50
    9.2 Statistical analysis of datasets 50
    9.3 Statistical analysis methods......51
10. Ethical norms and quality control 52
    10.1 Informed consent......52

10.2 Ethical norms and policies and regulations 52
10.3 Quality Assurance 52
11. References 53
12. The requirements for biological sample collection from the enrolled patients 53
12.1 Tissue sections 53
12.2 Blood collection requirements 54
12.3 Technical roadmap 54
Appendix 55
Appendix 1. ECOG performance status scoring criteria 55
Appendix 2. EORTC QLQ-C30 V3.0 56
Appendix 3. Response Evaluation Criteria in Solid Tumours (RECIST 1.1) criteria 59
Appendix 4. 2014 FIGO staging for ovarian cancer, fallopian tube cancer, and peritoneal cancer 74
Appendix 5. New York Heart Association (NYHA) classification 75

Synopsis

Title	Apatinib Combined with Standard Chemotherapy for Platinum-Resistant Recurrent Ovarian Cancer: The APPROVE Study
Sponsor	National Cancer Center/National Clinical Research Center for Cancer/Cancer Hospital, Chinese Academy of Medical Sciences and Peking Union Medical College
Principal investigator	Lingying Wu
Study population	Patients with platinum-resistant recurrent ovarian cancer
Study design	A randomized, parallel-controlled, multicentre, phase II study
Study objectives	Primary endpoint: progression-free survival (PFS)<br>Secondary endpoints: overall survival (OS), objective response rate (ORR), disease control rate (DCR), and safety
Treatment regimens	Drugs: doxorubicin hydrochloride liposomes, apatinib mesylate tablets<br>Treatment group (group A):<br>Doxorubicin hydrochloride liposomes: 40 mg/m^2 Q4W + apatinib (250 mg po qd);<br>Control group (group B):<br>Doxorubicin hydrochloride liposomes: 40 mg/m^2 Q4W;<br>1. The enrolled patients are required to have received at least 4 cycles of platinum-based first- or second-line chemotherapy and have experienced disease progression within 6 months after platinum-based chemotherapy.<br>2. Patients in groups A and B who achieve effective treatment will not receive more than 6 cycles of chemotherapy. Afterwards, group A will receive apatinib orally for maintenance therapy until disease progression or intolerance, and group B will be followed up. Patients who progress during treatment will stop treatment and withdraw from the study.
Sample collection requirements	3. Paraffin sections of tumours from the initial surgery will be collected from the enrolled patients, and paraffin sections of tumours from patients

	who underwent the initial surgery in other hospitals will be obtained from those hospitals.<br>Sectioning requirements: A total of 10 5-μm-thick or 5 10-μm-thick unstained tissue sections, of which nucleated cells compose more than 80%, with the local tumour cell content exceeding 70%, will be stored at room temperature (paraffin sections of tumours from patients enrolled in other sites will be sent to the Cancer Hospital of the Chinese Academy of Medical Sciences).<br>4. Peripheral blood collection time: Peripheral blood samples (10 mL) will be collected a total of 4 times, namely, before the first, third, and fifth cycles of chemotherapy and one month after the end of the sixth cycle of chemotherapy. If a patient withdraws from the study due to disease progression, another blood sample (10 mL) will be collected. For patients receiving maintenance treatment in the group A, blood samples (10 mL) will be collected during follow-up examinations every 2 months. All blood samples will be collected using 10-mL cfDNA sample storage tubes. After the blood samples are collected, the tubes will be turned upside down 10 times and stored at room temperature (blood samples from the patients enrolled in other sites shall be sent to the Cancer Hospital of the Chinese Academy of Medical Sciences within 5 days).
Planned total number of patients	126 patients (1:1)
Inclusion criteria	1. Previous pathological diagnosis of ovarian cancer, fallopian tube cancer, or primary peritoneal cancer, of which the pathological type is non-mucinous adenocarcinoma, with available paraffin sections from a previous surgery;<br>2. Platinum-resistant relapse (relapse within 6 months after the last chemotherapy)<br>3. Combined with malignant pleural effusion or ascites or with clinically

	evaluable recurrent lesions;<br>4. Eastern Cooperative Oncology Group (ECOG) performance status of 0 or 1;<br>5. Expected survival ≥ 4 months;<br>6. No history of anti-vascular targeted therapy;<br>7. Patients without pleural effusion or ascites confirmed by computed tomography or magnetic resonance imaging are required to have at least 1 measurable lesion as the target lesion based on the Response Evaluation Criteria in Solid Tumours (RECIST 1.1) criteria; if the target lesion is a lymph node, the diameter must be greater than 1.5 cm, and it must be unsuitable for surgical treatment; and the target lesion must be free of radiotherapy or relapsed within the radiotherapy field;<br>8. The baseline blood routine meets the following criteria:<br>a) Neutrophil count ≥ 1.5 x 10^9/L,<br>b) Platelet count ≥ 100 x 10^9/L, and<br>c) Haemoglobin ≥ 9 g/dL (allowing blood transfusion to achieve or maintain this level);<br>9. Liver function meets the following criteria:<br>a) Total bilirubin < 1.5 times the upper limit of normal (ULN);<br>b) Aspartate aminotransferase (AST) and alanine aminotransferase (ALT) < 2.5 × ULN in patients without hepatic metastasis and < 5 × ULN in patients with hepatic metastasis;<br>10. Serum creatinine ≤ 1.25 × ULN or the calculated creatinine clearance ≥ 50 mL/min;
Exclusion criteria	1. Having received more than 2 chemotherapy regimens;<br>2. Current or recent (within 30 days prior to enrolment) usage of another investigational drug or participating in another clinical study;<br>3. Other malignancies occurred within 5 years (except for fully treated cervical carcinoma in situ or skin squamous cell carcinoma or controlled

	basal cell carcinoma of the skin);<br>4. Hypertension that cannot be resolved, i.e. out of the normal range, by antihypertensive drug treatment (systolic blood pressure ≥ 140 mmHg or diastolic blood pressure ≥ 90 mmHg);<br>5. Grade II or higher myocardial ischaemia or myocardial infarction and poorly controlled arrhythmias (including heart rate-corrected QT (QTc) ≥ 450 ms for males and QTc ≥ 470 ms for females);<br>6. Previous or current cardiac insufficiency of grade II or above based on the New York Heart Association (NYHA) criteria, or the left ventricular ejection fraction (LVEF) lower than 50% or the lower limit of normal as evidenced by echocardiography;<br>7. Coagulation disorders [international normalized ratio (INR) >1.5, prothrombin time (PT) > ULN + 4 s, or activated partial thromboplastin time (APTT) >1.5 × ULN], bleeding tendency, or current thrombolysis or anticoagulation therapy;<br>8. Clinically significant bleeding symptoms or clear bleeding tendency, such as gastrointestinal bleeding, haemorrhagic gastric ulcer, baseline faecal occult blood of ++ or above, and vasculitis, within 3 months before randomization;<br>9. Major surgery or severe traumatic injury, fracture or ulcer within 4 weeks before randomization;<br>10. Factors that significantly affect the absorption of oral drugs, such as the inability to swallow, chronic diarrhoea, and intestinal obstruction;<br>11. Urinary protein ≥ ++ indicated by urinalysis or 24-h urinary protein ≥ 1.0 g;<br>12. Other conditions that may affect the clinical study or the interpretation of the study results according to the researcher's judgement;<br>13. Allergy to doxorubicin and/or related substances or the presence of idiosyncratic reactions;

	14. An expected cumulative dose of doxorubicin (including previous anthracyclines, if any) reaching or exceeding 550 mg/m^2 after 4 cycles of doxorubicin hydrochloride injection;<br>15. Uncontrollable arrhythmia or other ECG abnormalities indicative of a research risk, as determined by the principal investigator;<br>16. Doxorubicin liposome treatment in the past 6 months;<br>17. History of local radiation therapy of the pelvis or lower abdomen.
Criteria for termination or withdrawal	1. Patients can freely withdraw from the study at any time without any reason;<br>2. Concurrent radiotherapy on target lesions for efficacy observation;<br>3. Violation of the protocol that affects the evaluation of the results;<br>4. The investigators terminate the study due to safety profile among the enrolled patients [such as intolerable adverse events (AEs), pregnancy during treatment, or other events that affect the safety of the subjects];<br>5. When the inclusion/exclusion criteria are violated or the subject lacks compliance with or has to use unauthorized drugs, the investigator and/or the sponsor can decide to discontinue the treatment;<br>6. Progressive disease (PD);<br>7. Medical or ethical reasons that affect continuation in the study;<br>8. Poor quality data and incomplete and inaccurate information;<br>9. Patients receive other antitumour drugs during the clinical study period;<br>10. The sponsor terminates the study.
Efficacy evaluation	The contrast-enhanced thoraco-abdominal pelvic CT will be performed every 2 cycles (i.e., 8 weeks). Efficacy will be evaluated based on the RECIST 1.1 criteria or the Gynecologic Cancer Intergroup (GCIG) CA-125 criteria.
Safety evaluation	AEs will be graded and recorded in accordance with the National Cancer Institute's Common Terminology Criteria for Adverse Events (NCI-CTCAE) version 4.0. During the treatment period, a full physical

	examination, vital signs, laboratory safety evaluation, and AEs will be recorded at each visit.
Statistical analysis	Primary outcome measures: PFS will be analysed using the Kaplan-Meier method and the K-M plots will be provided. The median PFS and the corresponding 95% confidence interval will also be reported. The difference in survival between the two groups will be compared by using the log-rank test. Hazard ratio will be estimated by using the Cox proportional hazards model.<br>Secondary outcome measures: ORR and DCR will be described using a frequency table and two-sided 95% confidence intervals will be calculated by using Clopper-Pearson method. OS will be analysed using the Kaplan-Meier method and the K-M plots will be provided. The median OS and the corresponding 95% confidence interval will be reported. Hazard ratio will be estimated by using the Cox proportional hazards model. Safety: The number of occurrences and the incidence of safety events will be provided in a tabular format.
Study plan	Enrolment began in March 2018 and is expected to last for 2 years. The end of the study is defined as the completion of at least 2 efficacy evaluations for the last subject under continuous treatment or the occurrence of disease progression or intolerable toxicity.

7. Background

1.1 Progress in antiangiogenic therapy for recurrent platinum-resistant ovarian cancer

Ovarian cancer is a gynaecological malignancy with a poor prognosis. Although most ovarian cancer patients can achieve a clinical response after surgery and first-line chemotherapy, the vast majority of patients experience the painful process of recurrence, chemotherapy, recurrence, and retreatment and eventually develop drug resistance, leading to treatment failure. The main purpose of recurrent ovarian cancer treatment is to improve the quality of life and prolong the survival of patients. In the past 20 years, developments in surgery, chemotherapy and radiotherapy have played a limited role in improving the prognosis of patients with recurrent ovarian cancer. Targeted therapy is a medical treatment method that has emerged in recent years. Its emergence and development depend on the continuous exploration of tumourigenesis and development mechanisms via tumour molecular biology. Targeted therapy uses the characteristic changes in tumour cells at the molecular level as targets and exerts antitumour effects by interfering with molecules that play important roles in the development and progression of tumours. In the past 10 years, myriad clinical studies have evaluated the efficacy and side effects of targeted drugs for recurrent ovarian cancer and have achieved notable results.

Among the various types of targeted drugs, antiangiogenic drugs have been used the most to treat recurrent ovarian cancer, and encouraging results have been achieved. Angiogenesis is an indispensable step for the growth and metabolism of normal cells and tumour cells. However, the angiogenesis process and the composition and distribution of blood vessels in tumour tissues are not completely the same as those in normal tissues. Under the action of some angiogenic factors, tumour tissues are prone to vasodilation, local tissue pressure increases, and neovascular leakage, resulting in compromised oxygen and nutrient supplies. This compromise may be one of the reasons for the necrotic tendency in most solid tumours and may inhibit drug delivery to tumour tissues, thus affecting efficacy. In addition, endothelial cell proliferation, migration, and

invasion during tumour metastasis are closely related to a variety of angiogenic factors. Therefore, drugs targeting angiogenesis should have good prospects for antitumour therapy.

There are many factors involved in angiogenesis, of which vascular endothelial growth factor (VEGF) and its receptor (VEGFR) and angiogenin have become the main targets of existing drugs. Numerous studies have shown that VEGF and its receptors play important roles in both physiological and pathological angiogenesis. VEGF also plays an important role in the development of ovarian cancer and the formation of malignant ascites. VEGF has been reported to be overexpressed in 53% to 97% of ovarian cancers.[1] *In vitro* studies have shown that anti-VEGF drugs can inhibit angiogenesis in tumour tissues, reduce the tumour feeding, and slow tumour growth. In addition, anti-VEGF drugs can normalize the vascular structure of tumour tissues, promote the effective reach of chemotherapeutic drugs to tumour tissues, and have a synergistic effect with chemotherapy.

Bevacizumab is a recombinant, humanized monoclonal anti-VEGF antibody. It inhibits VEGF, suppressing angiogenesis and thereby resulting in 3 effects: (1) tumour vascular degeneration (cutting off the nutrient supply to tumour cells), (2) inhibition of angiogenesis and revascularization (continuous inhibition of residual and new tumour cells), and (3) normalization of surviving vessels (reducing plasma leakage and increasing drug delivery under interstitial pressure). The development of the theory of vascular dependence of tumours has formed a therapeutic strategy that emphasizes both anti-angiogenesis and anti-cell proliferation approaches.

In 2014, a randomized, open-label, phase III clinical trial (AURELIA trial) reported by Andres Poveda et al. from Spain showed that bevacizumab combined with monochemotherapy significantly improved the progression-free survival (PFS) of and objective response rate (ORR) for patients with recurrent platinum-resistant ovarian cancer (PROC).[2] In the AURELIA trial, patients were assigned to a control group (chemotherapy alone) or an experimental group (chemotherapy plus bevacizumab), and the chemotherapy regimen [paclitaxel, pegylated liposomal doxorubicin (PLD) or topotecan] for each patient was chosen by the investigators. The results indicated that

the PFS of the patients in the experimental group improved significantly. The hazard ratios (HRs) of PFS were 0.46 for patients who received paclitaxel (median, 10.4 months vs 3.9 months in the experimental and control groups, respectively), 0.57 for patients who received PLD (median, 5.4 months vs 3.5 months), and 0.32 in patients who received topotecan (median, 5.8 months vs 2.1 months). Different chemotherapy regimens combined with bevacizumab all demonstrated PFS benefits.

Based on the Response Evaluation Criteria in Solid Tumours (RECIST 1.1) criteria, patients who received bevacizumab plus paclitaxel (53.3% vs 30.2%) and patients who received bevacizumab plus topotecan (17.0% % vs 0) had a significantly higher ORR than did patients who received monochemotherapy, while patients who received PLD had a similar ORR to that for patients who received monochemotherapy (13.7% vs. 7.8%).

Patients' self-reported outcomes were evaluated using the European Organisation for Research and Treatment of Cancer (EORTC) Quality of Life Questionnaire-Ovarian Cancer Module (QLQ-OV28). The abdominal/gastrointestinal symptoms improved by ≥ 15% in the 8th and 9th weeks. In each cohort, the proportions of patients whose abdominal/gastrointestinal symptoms improved were higher among those who received bevacizumab combined with chemotherapy (25.0% of patients who received bevacizumab plus paclitaxel, 20.0% of patients who received bevacizumab plus topotecan, and 21.1% of patients who received bevacizumab plus PLD) than among those who received chemotherapy alone (13.0% of patients who received paclitaxel alone, 8.8% of patients who received topotecan alone, and 6.8% of patients who received PLD alone).

Based on the results of the phase III AURELIA trial, the Food and Drug Administration (FDA) approved bevacizumab for the treatment of recurrent PROC in 2014. Although bevacizumab has not been approved in China, it is a clinical treatment option for recurrent PROC. However, because of the high price of bevacizumab, many patients cannot afford it, limiting its clinical application to a certain extent. In summary, antiangiogenic therapy has broad application prospects in cancer treatment. However, more clinical studies are still needed to confirm the efficacy of antiangiogenic targeted

drugs in ovarian cancer.

1.2 Drug name

The chemical name of apatinib mesylate (abbreviated as apatinib) is N-[4-(1-cyanocyclopentyl)phenyl]-2-[(4-pyridylmethyl)amino]-3-pyridinecarboxamide methanesulfonate. Its molecular formula is $C_{25}H_{27}N_{5}0_{3}S$, with a molecular mass of 493.58 (methanesulfonate).

1.3 Results of preclinical studies of apatinib

1.3.1 Pharmacokinetics study

The results of the pharmacokinetics study in beagle dogs indicated that after a single dose of 5 mg/kg apatinib administered intravenously, the highest plasma concentrations (C_{max}) of apatinib were 6058 ng/kg in male beagle dogs and 3523 ng/mL in female beagle dogs. The area under the plasma concentration-time curve ($AUC_{0\to24\ h}$) was 12599 ng/mL·h (male) and 9106 ng/mL·h (female). The elimination half-lives ($T_{1/2}$) were 2.15 h (male) and 3.22 h (female), and the elimination rate constants (Kel) were 0.328 h^{-1} (male) and 0.216 h^{-1} (female). The mean retention times (MRTs) were 3.08 h (male) and 3.81 h (female). The total plasma drug clearance rates (CL) were 0.385 L/h/kg (male) and 0.515 L/h/kg (female). The apparent distribution volumes (Vd) were 1.19 L/kg (male) and 1.94 L/kg (female).

1.3.2 Preclinical pharmacodynamics study

In vitro: The sulforhodamine B (SRB) or 3-(4,5-dimethylthiazol-2-yl)-2,5-diphenyl tetrazolium bromide (MTT) assay was used to evaluate the growth inhibitory effect of apatinib on a variety of *in vitro* cultured cancer cells, such as colon cancer cells, lung cancer cells, gastric cancer cells, renal carcinoma cells and leukaemia cells. The half-maximal inhibitory concentration (IC_{50}) of apatinib against the above cancer cell lines exceeds 20 μM, a concentration much higher than that needed to inhibit tyrosine kinase receptors, including VEGFR, indicating that apatinib has no cytotoxicity. *In vivo*: Apatinib has a marked antitumour effect on a variety of human cancer xenografts in

nude mice, including colon cancer, lung cancer, and gastric cancer. In addition, apatinib enhances the efficacy of conventional cytotoxic drugs including oxaliplatin, 5-fluorouracil (5-FU), docetaxel and doxorubicin, and its efficacy is markedly better than that of PTK787 and comparable to that of ZD6474 and AMG706.

Antitumour mechanism: Apatinib can effectively inhibit VEGFR2 at extremely low concentrations and can inhibit kinases, such as PDGFR, c-Kit and c-Src, at high concentrations, as shown in the table below. Apatinib is 13.7 times more effective than PTK787 in inhibiting VEGFR2 activity. Moreover, apatinib inhibits downstream signal transduction mediated by VEGFR2. Apatinib also inhibits the growth of KDR/NIH3T3 high-expressing cell lines, VEGF-induced proliferation and migration of human umbilical vein endothelial cells and lumen formation, and microvascular genesis in rat arterial rings. The *in vitro* antiangiogenic effect of apatinib is stronger or equivalent to that of the control compound PTK787.

1.3.3 Toxicological studies of apatinib

1.3.3.1 Acute toxicity tests in animals

(1) Mice: A total of 40 ICR mice were randomly divided into a 5 g/kg dose group and a solvent control group, with 20 mice in each group and a male-to-female ratio of 1:1. An immediate response was observed after a single intragastric administration of apatinib. The mice were continuously observed for 14 days, and toxicity responses and death were recorded. All mice were dissected on the 15th day, and no drug-related changes were observed in various organs. During the observation period, except for the slightly slower body weight gain of the mice in the 5 g/kg dose group, no significant clinical toxic reactions or death were observed.

(2) Rats: A total of 80 SD rats were randomly divided into 4 groups, namely, a 2 g/kg apatinib group, a 5 g/kg apatinib group, and their respective control groups, with 20 rats in each group. An immediate response was observed after a single intragastric administration of apatinib. The mice were continuously observed for 14 days, and toxicity responses and death were recorded. During the observation period, the mortality rates in the 5 g/kg apatinib group were 50% in female rats and 20% in male

rats. In the 2 g/kg apatinib group, 1 female rat died; therefore, the mortality rate for female rats was 10%, while no male rats died. The anatomy of a dead female mouse in the 5 g/kg apatinib group revealed bilateral enlargement of the adrenal glands and yellow plaques in the right kidney. Hepatic and renal function tests revealed that alanine aminotransferase (ALT), aspartate aminotransferase (AST), and blood urea nitrogen (BUN) concentrations were elevated more than 3 times baseline levels. A pathological examination revealed scattered punctate necrosis of hepatocytes, adrenal haemorrhage, thymic atrophy, and lymphocyte reduction in the germinal centre of splenic white pulp. The other rats that died and the rats that were dissected after 14 days showed no obvious abnormalities.

(3) Beagle dogs: Six beagle dogs (body weight, approximately 7 kg; male-to-female ratio, 1:1) were orally administered apatinib. The doses for the 3 female dogs were 1050, 1575, and 2363 mg/kg (50% increments), and the doses for the 3 male dogs were 1575, 2363, and 3545 mg/kg (50% increments). After the administration of apatinib, female beagle dogs showed reduced activity, weakness in the limbs, and gait instability, and the female dog that received 2363 mg/kg apatinib exhibited a 5-fold increase in BUN levels. Male dogs began to vomit 4 h after the administration of apatinib, and they had reduced activity, weakness in the limbs, and reduced food intake. The degree of the toxic reaction increased with increasing dose, but no dog died.

1.3.2 Long-term toxicity test in animals

(1) Rats: Sprague-Dawley rats received apatinib intragastrically for 13 weeks, with a 4-week recovery period, during which they were observed. The rats were divided into 4 groups, namely, 3 dose groups [5, 15 and 50 mg/(kg·d) groups] and 1 solvent control group, with 14 female rats and 14 male rats in each group. No drug-related changes were observed in any rat in the 5 mg/(kg·d) group. The ALT levels in the male rats in the 15 mg/(kg·d) group were slightly elevated during the treatment period but recovered after the drug was discontinued. All rats in the 50 mg/(kg·d) group experienced slow body weight gain, decreased food consumption, weight loss, and mild increases in the liver function indicators ALT, AST, and ALP during the treatment period; however, no drug-related changes in organs were observed during pathological examinations. In

addition, the female rats in this group exhibited incisor fractures (11/14, 78.6%) and a mild reduction in tibial bone mineral density. After drug withdrawal, except for bone changes, which partially recovered, other changes fully reverted. Histological examinations revealed that compared with solvent control group, the 15 mg/(kg·d) group mainly suffered incisor fractures, which partially healed during the 4-week recovery period, and in severe cases, the incisors completely fell out. No drug-related pathological changes were observed in the other 2 dose groups.

(2) Beagle dogs: Twenty-four beagle dogs (body weight, approximately 7 kg; male-to-female ratio, 1:1) were randomly assigned into 4 groups, namely, 3 dose groups [3, 10, and 30 mg/(kg·d) groups] and a control group, with 6 dogs in each group (3 female dogs and 3 male dogs). The dogs were administered apatinib for 13 weeks. The haematology, blood biochemistry, electrocardiogram and bone mineral density indicators in each dose group were similar to those in the control group, and all fluctuated within the normal range, suggesting that liver function and kidney function were essentially normal. During the treatment period, for dogs in the 30 mg/(kg·d) group, the skin colour of the nose and corners of the mouth became pale, and the pathology indicated that their nasal skin became thinner; no abnormalities were observed in the control group.

1.4 Results of the phase I clinical trial of apatinib

1.4.1 Phase I apatinib drug tolerance study

In accordance with the modified Fibonacci method, patients who received apatinib were assigned to 1 of 5 dose groups, namely, a 250 mg group, a 500 mg group, a 750 mg group, an 850 mg group, and a 1000 mg group. Haematological toxicity above grade 4 and non-haematological toxicity above grade 3 were regarded as dose-limiting toxicity (DLT). A total of 18 patients were evaluable for tolerance to apatinib, including 6 patients in the 850 mg group and 3 patients in each of the other dose groups.

Apatinib DLT occurred in the 3 patients in the 1000 mg group, 2 of whom had grade III hypertension and 1 had grade III hand-foot skin reaction (HFSR). The patients recovered from the toxicity after dose interruption, and subsequently were

administrated a reduced dose to further control toxicity. No DLT occurred in the 6 patients in the 850 mg group. For further safety considerations, the observation time of patients in the 850 mg group was extended to 2 cycles; no DLT occurred. Therefore, 850 mg was determined to be the maximum tolerated dose (MTD).

The major adverse events (AEs) in the first cycle of the tolerance test included HFSR, hypertension, leukopenia, oral mucositis, fever, thrombocytopenia, fatigue, elevated bilirubin, headache, abdominal pain, nausea, and elevated transaminases. Most cases were mild to moderate. The main drug-related side effects included HFSR, leukopenia, hypertension and fever, elevated bilirubin, oesophagitis, skin toxicity, thrombocytopenia, nausea, fatigue, headache, elevated transaminases, oral mucositis, upper abdominal discomfort, tongue pain, hoarseness, stomach discomfort, chest tightness and coughing, proteinuria, and sinus bradycardia. In the 850 mg and lower dose groups, the haematological toxicities, including leukopenia and thrombocytopenia, were grade I-III, and non-haematological toxicities were grade I and II, all of which were cured after symptomatic treatment.

1.4.2 Phase I clinical pharmacodynamics observation of apatinib

From May 2007 to December 2008, a phase I clinical trial on apatinib was carried out at Fudan University Shanghai Cancer Centre; the trial specifically included a tolerance study, a pharmacokinetics study, and a phase I clinical supplementary study. A total of 81 patients with advanced solid tumours who either did not respond to or did not receive standard treatment were enrolled. Among them, efficacy was not evaluated in 9 patients, and the dose of apatinib to another 3 patients was low, only 250 mg/day. A total of 69 subjects received apatinib treatment at 500-1000 mg/day (only 3 subjects received 1000 mg/day), and a total of 56 patients were evaluable. The tumour types in the 69 subjects and the efficacy of apatinib in the 56 evaluable patients are provided in Table 1-2 below:

Table 1-2 The efficacy of apatinib on different solid tumours

	Gastric cancer	Colorectal cancer	Lung cancer	Breast cancer	Nasopharyngeal carcinoma	Kidney cancer	Oesophageal cancer	Liver cancer	Small intestinal stromal tumour	Malignant schwannoma of the left iliac fossa
NE	1	7	1	1	1	0	1	1	0	0
CR	0	0	0	0	0	0	0	0	0	0
PR	2	2	0	0	0	1	0	0	1	0
SD	5	15	3	4	1	0	4	1	0	0
PD	1	7	1	2	0	0	0	0	0	1
Death before evaluation	3	1	0	0	0	0	0	0	0	0
Objective response rate	18.1%	8%	0	0	0	100%	0	0	100%	0
Disease control rate	63.6%	68%	75%	66.7%	100%	100%	100%	100%	100%	0
Total	12	32	5	7	2	1	5	2	1	1

Results:

A total of 69 subjects with various types of solid tumours were included in the statistical analysis; the efficacy for 13 subjects was not evaluable. Among the 69 subjects included in the statistical analysis, the dose was 500 mg for 16 subjects, 750 mg for 37 subjects, 850 mg for 13 subjects, and 1000 mg for 3 subjects.

Based on the ORR, only 1 patient with renal cancer and 1 patients with a small intestinal stromal tumour were enrollable or evaluable, and they both achieved a PR. Eleven evaluable patients with gastric cancer and 25 evaluable patients with colorectal cancer were included, and they achieved high response rates of 18.1% and 8%, respectively.

Based on the disease control rate (DCR), only 1 patient with nasopharyngeal carcinoma, 1 patient with liver cancer, and 1 patient with a gastrointestinal stromal tumour were evaluable, and they all achieved stable disease (SD). Due to the small number of cases, efficacy should be interpreted with caution. Four patients with oesophageal cancer, 4 patients with lung cancer, and 6 patients with breast cancer were evaluable, and their DCRs were 100%, 75%, and 66.7%, respectively. However, due to the small number of cases, efficacy was difficult to evaluate.

1.4.3 Phase I clinical pharmacokinetics study of apatinib

Three doses, namely, 500, 750, and 850 mg/case, were used in single-dose oral administration tests. Seven males with tumours and 5 females with tumours were assigned to the low-dose group, 6 males with tumours and 3 females with tumours were assigned to the medium-dose group, and 6 males with tumours and 6 females with tumours were assigned to the high-dose group. In this study, all patients took apatinib 0.5 h after meals, and blood samples from multiple sites and urine samples were collected within 48 h after administration. The results indicated that M1 is a major metabolite of apatinib in humans; the AUC_{M1}/AUC of apatinib was 1.15-5.06. The 48-h cumulative urinary excretion of apatinib ($Cum.Ae_{M1}$/Cum.Ae) was 24.5-353. The $T_{1/2}$ values for apatinib and M1 were 8.93±0.81 h and 12.5±1.666 h, respectively. At the same dose, the individual differences in the levels of apatinib and M1 exposure (AUC and C_{max}) were large. The high-dose group showed significant sex differences ($P < 0.05$), including an AUC 1.96 times higher in females than in males and a C_{max} 3.57 times higher in females than in males. The exposure levels of apatinib and M1 in male and female subjects were nonlinearly but positively correlated with the oral dose of apatinib.

Food intake effect test: The focus of this test was to compare the difference between the absorption of apatinib by oral administration 1 h before food intake and 0.5 h after food intake. The *in vivo* concentration of M1 was also examined. In this test, blood samples from multiple sites and urine samples were collected within 48 h of the administration of apatinib. The results indicated that there were no significant differences in the blood T_{max} and C_{max} of apatinib between male and female subjects ($P > 0.05$). In addition, the AUC, $T_{1/2}$ and Cum.Ae of apatinib, as well as the related pharmacokinetics parameters of M1, were not affected by the order of food intake and the administration of apatinib ($P > 0.05$).

Because M1 has a longer half-life ($T_{1/2}$: 12.9±1.85 h) than apatinib, the multiple administration test focused on the *in vivo* accumulation of M1. In addition, the concentration of apatinib ($T_{1/2}$: 9.24±1.40 h) was observed during the test. In this test, the frequency of administration of apatinib was once per day, and the test period was 4

weeks. Blood samples from multiple sites and urine samples were collected on days 1, 14 and 28 after the start of the test. The test results indicated that although M1 and apatinib have long half-lives, no significant accumulation of M1 and apatinib ($P > 0.05$) was observed in the body of the subjects on day 14 after the start of the test compared with that on day 1 after the start of the test. However, the M1 exposure levels in male subjects increased significantly ($P < 0.05$) on day 28 compared with day 1. The AUC (1 d), AUC (14 d) and AUC (28 d) of apatinib were 9260±4308, 7256±4709, and 21930±20098 ng·h/ml, respectively. The C_{max} (1d) C_{max} (14 d), and C_{max} (28 d) of apatinib were 1285±776, 693±430, and 1602±1755 ng/ml, respectively. The AUC (1 d), AUC (14 d) and AUC (28 d) of M1 were 13846±7061, 10899±6702, and 39784±21900 ng·h/ml, respectively. The C_{max} (1 d), C_{max} (14 d) and C_{max} (28 d) of M1 were 1193±673, 908±597, and 1728±1233 ng/ml, respectively. There was no significant cumulative urinary and renal excretion of apatinib and M1 (P >0.05). The Cum.Ae (1 d), Cum.Ae (14 d) and Cum.Ae (28 d) of M1 were 30281±8141, 34537±15867, and 33466±17737 µg, respectively.

1.4.4 Safety data of apatinib

The results of the phase I trial of apatinib indicated that patients well tolerated the application of 850 mg apatinib alone, that the application of 250 mg, 500 mg and 750 mg apatinib was safe in patients, and that the initial dose of 250 mg was effective on tumours. The main manifestations of toxicity observed in the phase I trial included hypertension (10/16), hand-foot syndrome (HFS) (7/16), bone marrow suppression (7/16), oral ulcers (5/16), oesophagitis (2/16), chest and back pain (2/16), hoarseness (2/16), fatigue (2/16), diarrhoea (1/16), and proteinuria (1/16). In the 750 mg and lower dose groups, haematological toxicities, including leukopenia and thrombocytopenia, were grade I to III, and non-haematological toxicities were grade I and II; these toxicities were cured after symptomatic treatment. The results of phases II and III gastric cancer trials also confirmed that the main side effects to apatinib are leukopenia (36.36%), neutropenia (34.09%), thrombocytopenia (23.03%), proteinuria (44.32%), hypertension (35.23%), HFS (27.84%), fatigue (18.18%), loss of appetite (10.23%),

diarrhoea (8.52%), and hoarseness (7.93%). The incidence of unexpected side effects was low in the trials. Most side effects were transient or reversible after dose adjustment or drug discontinuation, and all of them were controllable.

Based on the results of clinical trials, the Food and Drug Administration of China (CFDA) approved apatinib for the treatment of advanced gastric cancer on November 17, 2014, becoming the first small-molecule targeted drug approved for the treatment of advanced gastric cancer in the world thus far.

In summary, based on the current research status and clinical need for antiangiogenic drugs to treat recurrent PROC, it is necessary to further explore the efficacy and safety of apatinib for the treatment of recurrent PROC.

8. Study purpose

(1) To clarify the efficacy of apatinib in patients with first recurrence of PROC.

(2) To clarify the molecular subtypes of the population who benefits from apatinib and screen the molecular markers that can assess the efficacy of apatinib, thus assisting in the selection of the target population for apatinib.

(3) To study the safety of apatinib, collect relevant safety information in the ovarian cancer population, and observe side effects.

2.1 Primary outcome measures

Progression-free survival (PFS) is defined as the time from randomization to tumour progression or death due to any cause.

2.2 Secondary outcome measures

- Overall survival (OS), defined as the time from the date of randomization to death (months). For subjects who are still alive or lost to follow-up by the cut-off date for data analysis, survival will be censored based on the last known survival time of the subject.
- ORR, defined as the proportion of patients whose tumour volume is reduced to a

predetermined value. The response period refers to the period from the first time of patients achieving CR or PR to disease progression. The imaging results will be evaluated according to the RECIST 1.1 criteria and classified as complete response (CR), partial response (PR), SD, and progressive disease (PD). The ORR is (CR+PR)%.

- DCR, defined as the proportion of the number of patients that have achieved a response (PR+CR) and SD after treatment to the number of evaluable cases. That is, DCR=CR+PR+SD.
- Safety.

9. Study design

This is a randomized, parallel-controlled, multicentre, phase II study. The planned number of enrolled patients is 126. Patients who meet the enrolment criteria will be randomly allocated to one of the following treatment regimens:

1) Doxorubicin hydrochloride liposomes 40 mg/m^2 D1 Q4W; or

2) Doxorubicin hydrochloride liposomes 40 mg/m^2 D1 Q4W plus apatinib (250 mg po qd)

The stratification factors include previous platinum-sensitive relapsed (yes vs. no) and platinum-free interval (≤3 vs. >3 months).

This study will be divided into 3 stages:

1. The baseline period (within 21 days before the start of treatment) – Patients will complete the screening examination during the baseline period to assess whether they meet the inclusion criteria;

2. Treatment period (from the first administration of apatinib to the completion of the last treatment cycle) – Tumours were evaluated every 8 weeks;

3. Follow-up period – After the end of the study treatment, the survival status and the follow-up antitumour treatment data will be collected by telephone follow-up or patient visits to the research centre every 3 months until death or loss to follow-up.

10. Subjects

4.1 Inclusion criteria

1. Previous pathological diagnosis of ovarian cancer, fallopian tube cancer, or primary peritoneal cancer, of which the pathological type is non-mucinous adenocarcinoma, and available paraffin sections from a previous surgery;

2. PROC that first reoccurs less than 6 months after the last chemotherapy;

3. Combined with malignant pleural effusion or ascites or with clinically evaluable recurrent lesions;

4. Eastern Cooperative Oncology Group (ECOG) performance status score of 0-1;

5. Expected survival ≥ 4 months;

6. No history of anti-vascular targeted therapy;

7. Pleural effusion or ascites confirmed by computed tomography (CT) or magnetic resonance imaging (MRI) according to the RECIST 1.1 criteria and at least 1 measurable lesion as the target lesion; in cases the target lesion is a lymph node, the target lesion must be greater than 1.5 cm, and it must be unsuitable for surgical treatment; and the target lesion must be free of radiotherapy or reoccurred within the radiotherapy field;

8. Baseline complete blood count meets the following criteria:

a) Neutrophil count ≥ 1.5 x 10^9/L;

b) Platelet (PLT) count ≥ 100 x 10^9/L; and

c) Haemoglobin ≥ 9 g/dL (allowing blood transfusion to achieve or maintain this level).

9. Liver function meets the following criteria:

a) Total bilirubin <1.5 times the upper limit of normal (ULN); and

b) AST and ALT < 2.5 × ULN in patients without hepatic metastasis and < 5 × ULN in patients with liver metastases; and

10. Serum creatinine ≤ 1.25 × ULN or the calculated creatinine clearance ≥ 50 mL/min.

4.2 Exclusion criteria

1. Having received more than 2 chemotherapy regimens;

2. Currently using or recently used (within 30 days prior to enrolment) another investigational drug or participating in another clinical study;

3. Other malignancies occurred within 5 years (except for fully treated cervical carcinoma in situ or skin squamous cell carcinoma or controlled basal cell carcinoma of the skin);

4. Hypertension that cannot be resolved, i.e., out of the normal range, by antihypertensive drug treatment (systolic blood pressure (SBP) ≥ 140 mmHg or diastolic blood pressure (DBP) ≥ 90 mmHg);

5. Grade II or higher myocardial ischaemia or myocardial infarction and poorly controlled arrhythmia (including heart rate-corrected QT (QTc) ≥ 450 ms for males and QTc ≥ 470 ms for females);

6. Previous or current cardiac insufficiency of grade II or above based on the New York Heart Association (NYHA) criteria or left ventricular ejection fraction (LVEF) lower than 50% or the lower limit of normal as evidenced by echocardiography;

7. Coagulation disorders [international normalized ratio (INR) >1.5, prothrombin time (PT) >ULN + 4 s or activated partial thromboplastin time (APTT) >1.5 × ULN], bleeding tendency, or current thrombolysis or anticoagulation therapy;

8. Clinically significant bleeding symptoms or clear bleeding tendency, such as gastrointestinal bleeding, haemorrhagic gastric ulcer, baseline faecal occult blood of ++ or above, and vasculitis, within 3 months before randomization;

9. Major surgery or severe traumatic injury, fracture or ulcer within 4 weeks before randomization;

10. Factors that significantly affect the absorption of oral drugs, such as inability to swallow, chronic diarrhoea, and intestinal obstruction;

11. Urinary protein ≥ ++ indicated by urinalysis or 24-h urinary protein ≥ 1.0 g;

12. Other conditions that may affect the clinical study or the interpretation of the study results;

13. Allergy to doxorubicin and/or related substances or the presence of idiosyncratic reactions;

14. The expected cumulative dose of doxorubicin (including previous

anthracyclines, if any) reaches or exceeding 550 mg/m^2 after 4 courses of doxorubicin hydrochloride injection;

15. Uncontrollable arrhythmia or other ECG abnormalities indicative of research risk, as determined by the principal investigator;

16. Doxorubicin liposome treatment in the past 6 months; and

17. History of local radiation therapy of the pelvis or lower abdomen.

4.3 Criteria for termination or withdrawal

1. Patients can freely withdraw from the study at any time without any reason;

2. Concurrent radiotherapy on target lesions for efficacy observation;

3. Violation of the protocol that affects the evaluation of the results;

4. The investigators terminate the study due to safety events among the enrolled patients (such as intolerable AEs, pregnancy during treatment, or other events that affect the safety of the subjects).

5. When the inclusion/exclusion criteria are violated, or the subject lacks compliance with or has to use unauthorized drugs, the investigator and/or sponsor can decide to discontinue the treatment;

6. PD;

7. Medical or ethical reasons that affect the continuation of the study;

8. Poor quality data and incomplete and inaccurate information;

9. The patients receive other antitumour drugs during the clinical study period; and

10. The sponsor terminates the study.

Once a subject withdraws, the investigator will record the reason for withdrawal on the subject's case report form (CRF) and medical record. All subjects who withdraw due to AEs or abnormal clinical laboratory test results will followed up until the subjects recover or achieve SD, and the subsequent outcomes will be recorded. The efficacy will not be evaluated for participants who withdraw due to side effects, but their adverse drug reactions (ADRs) will be included in the statistics.

4.4 Relevant post-withdrawal regulations for patients in the monochemotherapy group

After patients with doxorubicin liposomal monochemotherapy withdraw from the group due to disease progression during treatment, they can select the paclitaxel monochemotherapy regimen at 80 mg/m^2 qw based on their actual clinical needs. If patients have no contraindications for apatinib and have the desire to use apatinib, they can receive apatinib in combination with the paclitaxel monochemotherapy regimen under the guidance and follow-up of the clinicians following the same medication principle used for the group receiving combined therapy. Apatinib will be given to these patients free of charge. During treatment, patients will be encouraged to pay close attention to side effects and record them and to undergo regular laboratory tests. The researchers will provide close follow-up regarding the side effects and laboratory indexes of patients and provide corresponding treatments in a timely manner.

4.5 Early termination of the research project

If any of the following situations occur, the study can be terminated early after a discussion and judgement by the project team:

1) New information leads to adverse risk-benefit outcomes for the investigational drug, such as obtaining evidence that the investigational treatment is ineffective and observing any significant but previously unknown side effects or unexpected increases in the severity or incidence of known side effects or other evidence of poor safety;

2) The project undertaker believes that the research cannot be continued for both medical and ethical considerations; and

3) The investigational drug is withdrawn from the market for safety reasons.

11. Study schedule and evaluation

A signed and dated ICF will be obtained from each patient before any screening procedure is performed. The ICF will be reviewed and approved by an ethics committee.

5.1 Screening examination and baseline evaluation (will be completed within 21 days before the start of treatment)

After the patients sign the ICF, the following items will be examined to determine whether they meet the inclusion criteria:

Evaluation items	Key inspection items	Time from evaluation to the first dose
1. Written informed consent form	Signature	Signed before the first dose
2. Disease history and physical examination	The medical history should include the present medical history, past medical history, and history of allergies; the physical examination should include a pelvic examination.	7 days
3. CBC (venous blood)	Haemoglobin, platelet count, white blood cell count, neutrophil count	3 days
4. Biochemical profile	Blood biochemistry (ALT, AST, AKP, BIL, BUN, and Cr)	7 days
5. Urinalysis	Urine pH, urinary protein, urinary red blood cells, and urinary white blood cells	14 days
6. Stool test	Occult blood, red blood cells, and white blood cells	14 days
7. Pregnancy test	Pregnancy test (test of urine β-HCG or blood β-HCG in women of childbearing age who have not undergone total hysterosalpingo-oophorectomy)	14 days
8. Tumour evaluation	CT or MRI	21 days
9. Tumour marker	CA125	7 days
10. Quality of Life evaluation	EORTC QLQ-C30	7 days
11. ECG	Heart rate, heart rhythm, QTc interval	7 days
12. Cardiac function	Echocardiography	21 days

5.2 Evaluation of the treatment period

The combination regimen will be applied for a maximum of 6 cycles. After the

completion of the combination regimen, patients with CR, PR or SD will be treated with apatinib monotherapy. The application of apatinib monotherapy will be continued until disease progression or intolerance.

1. The following examinations and evaluations will be completed 5-7 days before the start of each treatment cycle:

(1) CBC, biochemical, and coagulation tests;

(2) Urinalysis and stool test;

(3) Evaluation of tumour markers;

(4) Concomitant medication evaluation;

(5) Physical examination (including body weight) and vital signs;

(6) ECOG physical performance status evaluation;

(7) 12-lead ECG and echocardiography;

(8) Evaluation of peripheral neurotoxicity; and

(9) Evaluation of AEs and clinical toxicity.

2. During treatment, CBC and urinalysis will be performed at least once per week.

3. Contrast-enhanced thoracic-abdominal-pelvic CT examinations will be performed before and after the first, third, and fifth cycles of treatment:

Tumour evaluations will always use the same method used for the baseline evaluation. If possible, for patients who have achieved an objective response (CR or PR), efficacy will be confirmed no less than 4 weeks after the first response.

4. The number of CBCs, blood biochemical tests, and urinalyses can be increased based on clinical needs. If the results are out of the normal range, the investigators will re-examine the results as appropriate. Any treatment-related abnormality in clinical examination or laboratory test results will be followed until the abnormality subsides or has no clinical significance.

5.3 End-of-study evaluation

When a patient decides to exit the study due to disease progression, the following evaluations will be performed when the patient withdraws from the trial:

1. Concomitant medication evaluation;

2. Physical examination;

3. ECOG performance status score;

4. Evaluation of peripheral neurotoxicity;

5. Evaluation of AEs;

6. 12-lead ECG and echocardiography;

7. Clinical laboratory tests (including CBC, coagulation, blood biochemistry, tumour markers, urinalysis, stool test, etc.); and

8. Imaging examination of tumours (CT or MRI).

5.4 Follow-up

All reasons for and dates of study discontinuation will be recorded in the CRF (e.g., loss to follow-up, patient refusal, and AEs). AE reporting will be completed within 30 days after the last dose of the investigational drug. The AEs related to the investigational drug will be monitored, and the outcomes will be recorded until the AEs resolve, until the correlation does not hold, or until the investigators judge that no further follow-up is needed from the medical point of view. The corresponding treatments for these AEs will be recorded in the CRF. After the evaluation, each enrolled patient will enter the follow-up period. The study physicians will collect information on survival status by phone follow-up or patient visits to the research centre every 2-3 months.

5.5 Evaluation of early exit and withdrawal

Patients who request early exit during the treatment period will receive a study exit evaluation. The reason for and date of exit will be recorded in the patient's medical record and CRF. When a patient withdraws from the study, the investigators will make every effort to complete all withdrawal-related examinations.

5.6 Study duration

The enrolment period is planned to be 12 months. The end of the study is defined as the completion of at least 2 efficacy evaluations for the last subject under continuous treatment or the occurrence of disease progression or intolerable toxicity.

5.7 Study schedule

**Evaluation content**	**Screening period**[1]	**During treatment**							
		**1st cycle**	**2ndcycle**	**3rdcycle**	**4thcycle**	**5thcycle**	**xth cycle**	**(x+1)th cycle**	**…….**[8]
	(-21 ±5 days)	Day 28±5[2]	Day 56±5	Day 84±5	Day 112±5	Day 140±5	Day 28x±5	Day 28(x+1)± 5	Efficacy will be evaluated once every 2 cycles
Written informed consent form	✓								
Demographics	✓								
Medical history	✓								
Inclusion/exclusion criteria	✓								
**Efficacy evaluation**									
Disease evaluation/tumour measurement (CT, MRI, etc.)	✓		✓		✓		✓		✓ Within the last 5 days of even-numbered cycles
Tumour marker (CA125)	✓	✓	✓	✓	✓	✓	✓	✓	✓ Within the last 5 days of even-numbered cycles

EORTC QLQ-C30 (V3.0) Chinese version	✓		✓		✓		✓		✓
**Safety evaluation**									
Concomitant medication	✓	✓	✓	✓	✓	✓	✓	✓	✓ Record at any time
Physical examination[3]	✓		✓		✓		✓		✓
Vital signs	✓	✓	✓	✓	✓	✓	✓	✓	✓
ECOG performance status	✓	✓	✓	✓	✓	✓	✓	✓	✓
Weight	✓	✓	✓	✓	✓	✓	✓	✓	✓
Height	✓								
ECG	✓	✓	✓	✓	✓	✓	✓	✓	✓
Echocardiography[4]	✓	✓	✓	✓	✓	✓	✓		
**Laboratory evaluation**									
CBC[5]	✓	✓	✓	✓	✓	✓	✓	✓	✓
Urinalysis[5]	✓	✓	✓	✓	✓	✓	✓	✓	✓
Biochemical profile[6]	✓	✓	✓	✓	✓	✓	✓	✓	✓
Stool test[7]	✓		✓		✓		✓		✓
Prothrombin time[8]	✓	✓	✓	✓	✓	✓	✓	✓	✓
**Investigational drug**									
Doxorubicin hydrochloride liposome		✓	✓	✓	✓	✓	✓		
Apatinib		✓	✓	✓	✓	✓	✓	✓	✓
Adverse event observation	Recorded at any time until 5 days after the end of treatment								
Study termination[10]	Record at any time								

Note:

1. Screening will be evaluated within a specified time frame before the first use of the investigational drug.

2. Laboratory evaluations will be carried out within 5 days before the use of investigational

drugs but will be repeated only when clinically necessary.

3. Physical examinations will be performed within 7 days before the visit at the earliest.

4. Echocardiography can be discontinued after treatment with doxorubicin hydrochloride liposomes.

5. The number of CBCs [haemoglobin, white blood cell (WBC) count, absolute neutrophil count (ANC), and platelet (PLT) count] and urinalyses (urinary pH, urine protein, urinary red blood cells, and urinary WBC) can be increased based on clinical needs. CBC should be performed within 3 days before each treatment.

6. The number of blood biochemical tests, including liver function tests (ALT, AST, and TBIL), renal function tests (BUN and Cr), and electrolyte tests (serum potassium, sodium, and chloride) can be adjusted based on clinical needs. The laboratory screening results will be reviewed. If the results are out of the normal range, the investigator will re-examine the results as appropriate. Subjects exhibiting treatment-related abnormalities in clinical examinations or laboratory tests will be followed up until the abnormalities have subsided or are no longer clinically significant.

7. Examinations will be performed once during the screening period and then performed as needed by the investigators.

8. Apatinib (250 mg, qd) will be administered until disease progression or intolerable toxicity.

9. The combination regimen can be used for a maximum of 6 cycles. After the completion of the combination regimen, patients with CR, PR or SD will be treated with apatinib monotherapy. Apatinib monotherapy will be continued until disease progression or intolerance.

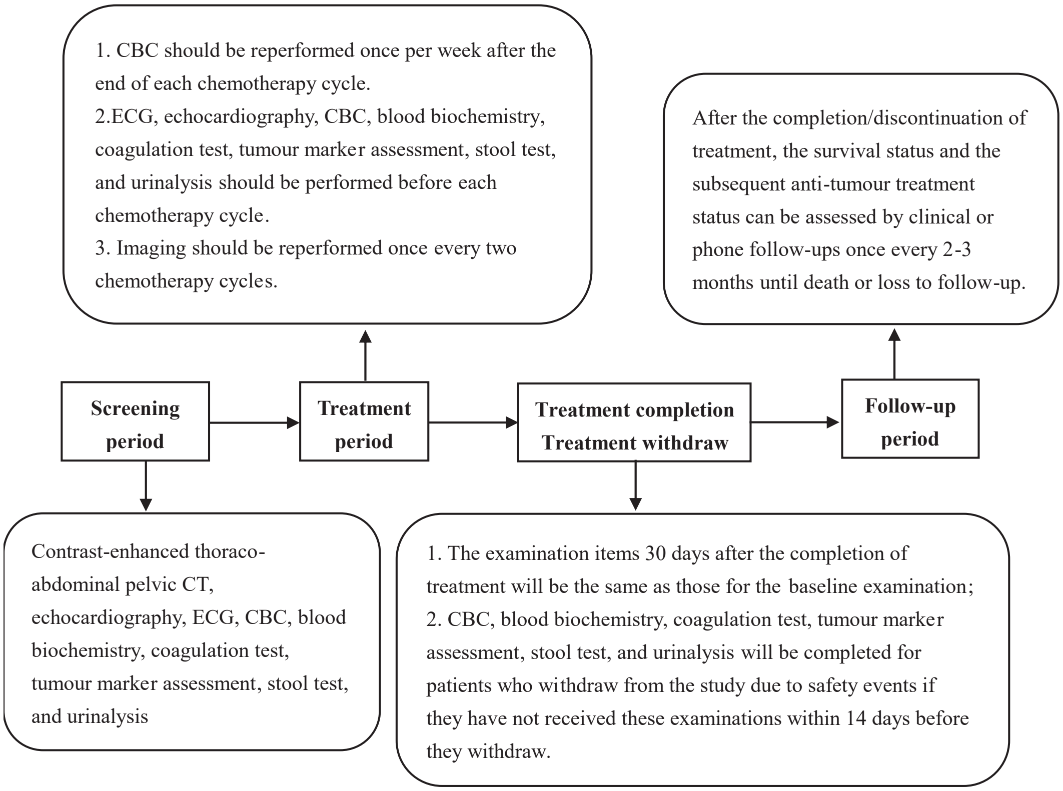

12.Investigational drug

6.1 Treatment regimens

The 126 patients were divided into 2 groups, the patients were randomly assigned to the following 2 treatment regimens by 1:1:

Group A: doxorubicin hydrochloride liposomes 40 mg/m^2 D1 (1 course of treatment = 28 days) + apatinib (250 mg po qd) * 6 cycles; the combination regimen can be applied for a maximum of 6 cycles. After the completion of the combination regimen, patients with CR, PR or SD will be treated with apatinib monotherapy. Apatinib monotherapy will be continued until disease progression or intolerance; and

Group B: doxorubicin hydrochloride liposomes 40 mg/m^2 D1 (1 course of treatment = 28 days) * 6 cycles.

6.2 Dose adjustment

6.2.1 Criteria for drug discontinuation or dose reduction

If a patient has side effects between 2 sequential follow-up visits, the patient should contact the doctor as soon as possible to determine the next treatment regimen. Drug discontinuation and dose reduction are only allowed when haematological toxicity reaches grade III or non-haematological toxicity reaches grade II or above. For non-haematological toxicity, nausea, vomiting and fever with definite cause (below 38℃) should be actively treated symptomatically without drug discontinuation or a dose reduction.

If a severe adverse reaction occurs, the dose will be adjusted or paused until the next cycle. If severe haematological and non-haematological toxic reactions occur, the dose of the drug with a significant causal relationship with the toxic reaction will be adjusted; the dose of only one drug will be adjusted per cycle. The dose of doxorubicin hydrochloride liposomes for intravenous chemotherapy will only be reduced twice (the dose will be reduced by 25% of the standard dose each time), and the dose of apatinib for oral chemotherapy will only be reduced once (from 250 mg, po, qd to 250 mg, po, qod). Otherwise, the patient must withdraw from the study.

During treatment, if a subject has not recovered from a drug toxicity, drug administration will be suspended. The cumulative duration of drug discontinuation for each dosing cycle will not exceed 2 weeks, and the drug discontinuation will not exceed 2 times within each cycle to maintain the drug concentration in each subject who receives treatment. The dose will be delayed for no more than 2 weeks; otherwise, the subject must withdraw from the study.

If multiple toxic reactions occur in 1 treatment cycle, the dose will be adjusted based on the highest grade of toxic reactions. For patients who receive combination therapy, if a toxic reaction is related to 2 drugs, the dose of oral apatinib will be reduced first. After the 1-time apatinib dose reduction, if the toxic reaction persists or is not reduced to grade 1, the dose of doxorubicin hydrochloride liposomes will be reduced no more than 2 times.

6.2.2 Details of drug discontinuation or dose reduction

To ensure the consistency of dose adjustments throughout the study, drug discontinuation will occur before dose reductions in each dosing cycle. The rules for

drug discontinuation and dose reductions are provided in the table below (as long as the conditions for dose reduction are met, the drug is considered poorly tolerated, and the dose must be reduced).

Rules for dose adjustment

NCI Common Toxicity Criteria	Dose adjustment for haematological toxicity
Grade I haematological toxicity	Maintain the original dose level (appropriate symptomatic treatment)
Grade II haematological toxicity	Maintain the original dose level (appropriate symptomatic treatment)
Grade III haematological toxicity	First, the drug will be suspended for symptomatic treatment. When toxicity decreases to ≤ grade II, the dose will be reduced by 1 level, and administration will be resumed.
Grade IV haematological toxicity	First, the drug will be suspended for symptomatic treatment. When the toxicity decreases to ≤ grade II, the dose will be reduced by 1 level, and administration will be resumed.
	Dose adjustment for non-haematological toxicity
Grade I non-haematological toxicity	Maintain the original dose level (appropriate symptomatic treatment)
Grade II non-haematological toxicity	Symptomatic treatment will be performed without discontinuing the drug. If the toxicity decreases to ≤ grade I 2 weeks after symptomatic treatment, drug administration will continue; if the toxicity does not improve or is aggravated, drug administration will be suspended, and the treatment will be continued at the original dose level when the toxicity decreases to ≤ grade I.
Grade III non-haematological toxicity	The drug will be suspended for symptomatic treatment. When the toxicity decreases to ≤ grade I, the treatment will be continued at the original dose, or the investigators may reduce the dose by 1 level as appropriate. If grade III or above side effects reoccur, the treatment will be discontinued, and the subject should withdraw from the clinical study.
Grade IV non-haematological toxicity	Drug administration will be suspended for symptomatic treatment. When the toxicity decreases to ≤ grade I, the treatment will be continued at the original dose. If grade IV toxicity reoccurs, the treatment will be discontinued, and the subject should withdraw from the clinical study (if life-threatening side effects, including grade IV renal damage, neurotoxicity, heart toxicity, and liver toxicity, occur, the treatment will be immediately discontinued, and the subject should withdraw from the clinical study).
Side effects that require special attention	
If hypertensive crises, cerebral haemorrhage, ≥ grade II pulmonary haemorrhage, ≥ grade III haemorrhage elsewhere, arterial thrombosis, grade IV venous thrombosis, leukoencephalopathy syndrome, or gastrointestinal perforation occur, the treatment will be immediately discontinued, and the subject should withdraw from the clinical study and receive active symptomatic treatment.	

6.2.3 Symptomatic treatment and dose adjustment schemes for common side effects

6.2.3.1 Non-haematological side effects

3) HFS:

During the administration of apatinib, the subjects should avoid mechanical injury and friction on the palms and soles by wearing loose and breathable shoes and soft cotton gloves and socks and using gel insoles and avoid physically strenuous exercise. The exposure of the hands and feet to high heat and direct sunlight should be avoided. The skin should be protected using moisturizers that contain lanolin or urea. Spicy foods should be avoided. If moderate HFSR occurs, some necessary supportive and symptomatic treatments can be provided, e.g., routine skin care, cleansing and moisturization, the avoidance of secondary infection, compression, mild abrasives, and mild moisturizer or lubricants. Topical exfoliators, including urea ointments, urea creams, 5% salicylic acid preparations, and corticosteroid-containing emulsions or lubricants can be applied. If necessary, the affected skin area can be soaked in magnesium sulphate dissolved in warm water. Topical antifungal or antibiotic treatment can be applied. B vitamins (B1, B6, and riboflavin) and celecoxib can be administered as appropriate.

If HFS occurs during the treatment period, the treatment dose can be adjusted or delayed based on the scheme detailed in Table 1.

Table 1 Dose adjustment scheme for hand-foot syndrome (HFS)

Toxicity grade	Dose adjustment
Grade 1 (mild erythema, oedema, or peeling that does not interfere with daily activities)	The dose will be continued unless the patient has a history of grade 3 or 4 HFS. If the patient has a history of grade 3 or 4 HFS, administration will be delayed for 2 weeks, and the dose will be reduced once before the normal dose interval is resumed.
Grade 2 (erythema, oedema, or peeling that interferes with but does not prevent normal activities; small blisters or ulcers less than 2 cm)	The dose will be delayed for 2 weeks or until the symptoms return to grade 0 or 1. If the symptoms do not improve after 2 weeks, the drug will be discontinued. If the symptoms decrease to grade 0 or 1 within 2 weeks and there is no history of grade 3 or 4 HFS, the previous dose and interval will be continued. If the patient has a history of grade 3 or 4 hand-foot-mouth syndrome, the dose will be reduced once before the normal dosing interval is resumed.
Grade 3 (fever, ulcer or swelling	The dose will be delayed for 2 weeks or until the symptoms

that interferes with walking or normal activities and prevents the participant from dressing normally)	decrease to grade 0 or 1. The dose will be reduced once before the normal dosing interval is resumed. If the symptoms do not improve after 2 weeks, the drug will be discontinued.
Grade 4 (spread or local infection, or bedridden or hospitalized)	The dose will be delayed for 2 weeks or until the symptoms decrease to grade 0 or 1. The dose will be reduced once before the normal dosing interval is resumed. If the symptoms do not improve after 2 weeks, the drug will be discontinued.

4) Oral mucositis:

The subjects are recommended to maintain their oral hygiene during the study period, rinse their mouth with salt water after meals, and brush their teeth before going to bed. Patients with mild oral mucositis should avoid eating hard, cold, hot, and spicy food. For moderate oral mucositis, dose adjustment is not required, and topical antibacterial agents and mucosal protective agents (sucralfate, honey, aloe vera) will be used for symptomatic treatment. For severe oral mucositis, doxorubicin hydrochloride liposomes will be discontinued. For patients with severe ulcers, compound chlorhexidine and dexamethasone pellicles will be used. For patients with severe pain, local anaesthetics will be used as appropriate for pain relief, and 1% lidocaine can be used as a gargle. If oral mucositis occurs during the treatment period, the treatment dose can be adjusted or delayed based on the scheme detailed in Table 2.

Table 2 Dose adjustment scheme for oral mucositis

Toxicity grade	Dose adjustment
Grade 1 (painless ulcer, erythema, or mild pain)	The dose will be continued unless the patient has a history of grade 3 or 4 oral mucositis. If the patient has a history of grade 3 or 4 oral mucositis, the dose will be delayed for 2 weeks. The dose will be reduced once before the normal dosing interval is resumed.
Grade 2 (painful erythema, oedema or ulcer but able to eat)	The dose will be delayed for 2 weeks or until the symptoms decrease to grade 0 or 1. If the symptoms do not improve after 2 weeks, the drug will be discontinued. If the symptoms decrease to grade 0 or 1 within 2 weeks and the patient has no history of grade 3 or 4 oral mucositis, then treatment will resume with the previous dose and interval. If the patient has a history of grade 3 or 4 oral mucositis, the dose will be reduced once before the normal dosing interval is resumed.

Grade 3 (painful erythema, oedema, or ulcer that causes an inability to eat)	The dose will be delayed for 2 weeks or until the symptoms decrease to grade 0 or 1. The dose will be reduced once before the normal dosing interval is resumed. If the symptoms do not improve after 2 weeks, the drug will be discontinued.
Grade 4 (necessity for enteral or parenteral nutrition support)	The dose will be delayed for 2 weeks or until the symptoms decrease to grade 0 or 1. The dose will be reduced once before the normal dosing interval is resumed. If the symptoms do not improve after 2 weeks, the drug will be discontinued.

3) Hypertension:

The US National Cancer Institute (NCI) cardiovascular toxicity research group recommends that the baseline blood pressure of subjects should be determined before the start of treatment with VEGF/VEGFR inhibitors. During treatment, every effort will be made to keep blood pressure stable and <140/90 mmHg. Blood pressure monitoring will be initiated before dosing and throughout the entire treatment process, especially during the initial 2 weeks of treatment, when daily monitoring will occur. The subjects will be fully informed when their blood pressure is >140/90 mmHg, and when subject experience symptoms associated with increased blood pressure (such as headache, dizziness, and visual impairment), they should contact the investigators immediately for guidance.

The selection of antihypertensive drugs will follow the relevant guidelines for the prevention and treatment of hypertension, with reference to the risk of cardiovascular events for the subjects. For patients with proteinuria, angiotensin-converting enzyme inhibitors (ACEIs) and angiotensin receptor antagonists (ARBs) will be recommended. Regarding drug selection, it should be noted that apatinib is mainly metabolized by the CYP3A4 enzyme in the liver; therefore, non-dihydropyridine calcium channel blockers (CCBs, such as verapamil and diltiazem) that can inhibit the CYP3A4 system are not recommended for blood pressure control. If grade 3-4 hypertension occurs, antihypertensive treatment will be actively carried out under the guidance of a cardiovascular specialist, and the dose of apatinib will be closely observed and/or adjusted. If hypertension is still not well controlled, drug administration should be stopped. For patients in a hypertensive crisis, the drug will be discontinued immediately and permanently.

For patients with hypertension during the treatment period, the dose can be adjusted or delayed based on the scheme detailed in Table 3.

Table 3 Hypertension criteria (NCI-CTCAE 4.03) and prevention and treatment recommendations

Grade	Definition	Prevention and treatment recommendations
1	SBP = 120-139 mmHg<br>or DBP = 80-89 mmHg	Blood pressure will be closely monitored. Salt intake should be limited, and smoking and alcohol use should be stopped. The administration of apatinib will continue without dose adjustment.
2	SBP = 140-159 mmHg<br>or DBP = 90-99 mmHg	Blood pressure will be monitored closely. The administration of apatinib will continue without dose adjustment. Antihypertensive drugs (ACEI/CCB/ARB antihypertensive drugs are recommended) will be used for treatment and should not be discontinued without medical advice.
3	SBP ≥ 160 mmHg,<br>or DBP ≥ 100 mmHg	The administration of apatinib will be suspended. Cardiovascular specialists will be consulted for treatment, and the combined use of antihypertensive drugs will be considered. Blood pressure will be monitored closely. If the drug is postponed for more than 2 weeks, the subject should stop the medication and withdraw from the study. If the blood pressure returns to normal and is well controlled within 2 weeks, the dose of apatinib will be reduced once. If grade 3 or above hypertension occurs, the subject should stop the medication and withdraw from the study.
4	Life-threatening (malignant hypertension or persistent nerve damage, hypertensive crisis)	The administration of apatinib will be discontinued immediately and permanently. Cardiovascular specialists will be consulted for the active management of hypertension, and blood pressure and other vital signs will be monitored closely.

4) Proteinuria:

If a subject has grade 2 or above proteinuria while receiving apatinib, apatinib will be suspended until recovery to normal. If grade 2 or above proteinuria reoccurs after resuming the medication, the dose of apatinib will be reduced by 1 dose unit; if the proteinuria persists and worsens, the medication will be discontinued. Once renal dysfunction or nephrotic syndrome occurs, the medication will be discontinued immediately, and supportive and symptomatic treatment will be provided.

Currently, there is no clear treatment regimen for anti-angiogenesis inhibitor-

induced proteinuria. However, ACEIs and ARBs can be used as appropriate because these drugs can reduce proteinuria and possible adverse cardiac events by reducing intratubular pressure.

When proteinuria occurs during the treatment period, the dose can be adjusted or delayed based on the scheme detailed in Table 4.

Table 4 Proteinuria criteria (NCI-CTCAE 4.03) and prevention and treatment recommendations

Grade	Definition	Prevention and treatment recommendations
1	Urine protein (+) or 24-h urine protein < 1.0 g	The administration of apatinib will continue without a dose adjustment. The condition of the patient will be observed closely.
2	Urine protein (++) or 24-h urine protein = 1.0-3.4 g	The administration of apatinib will continue without a dose adjustment. Drug intervention will be considered. The 24-h urinalysis results and 24-h urine protein will be monitored.
3	24-h urine protein > 3.4 g	Apatinib administration will be suspended. Specialists in nephrology will be consulted. Drug intervention will be performed. After the proteinuria decreases to grade 2 or below, apatinib will be continued at a reduced dose. If grade 3 proteinuria still occurs, apatinib treatment will be permanently discontinued.

5) Bleeding

During the administration of apatinib, PT and INR will be closely monitored, and bleeding tendencies and related symptoms will be closely monitored. If a serious abnormality (grade 3-4) occurs, drug administration will stop. If upper gastrointestinal bleeding occurs, apatinib will be discontinued immediately, and the bleeding will be treated in accordance with acceptable clinical practices.

6) Thrombosis

If any arterial thrombosis occurs (such as cerebral ischaemia, stroke, angina pectoris, and myocardial infarction), drug administration will be discontinued immediately, and the subject should withdraw from the study. If grade IV symptomatic venous thrombosis occurs, the drug will be discontinued, and the subject should withdraw from the study. Symptomatic treatment, surgery, or anticoagulant medication will be applied immediately to treat thrombotic symptoms.

The dose adjustment scheme for venous thrombosis is provided in Table 5.

Table 5 Dose adjustment scheme for venous thrombosis

Venous thrombosis	Dose adjustment rules
Grade II (uncomplicated deep venous thrombosis that requires medical intervention)	The original dose of apatinib will be maintained, and the condition of the patient will be monitored closely.
Grade III (uncomplicated venous pulmonary embolism or non-embolic cardiovascular thrombosis that requires medical intervention) or grade IV (pulmonary embolism, cerebrovascular events, arterial insufficiency, or haemodynamic or neurological dysfunction that requires acute intervention)	1) The administration of apatinib will be suspended.<br>2) Anticoagulants (small-molecular-weight heparins) will be administered.<br>3). The anticoagulants will be administered for at least 1 week. After the thrombotic symptoms improve without severe bleeding (grade III or IV), apatinib will be continued at a reduced dose (1 dose level less) based on the investigator's judgement.

6.2.3.2 Haematological toxicity

The common haematological side effects to doxorubicin hydrochloride liposomes include leukopenia and thrombocytopenia. When mild haematological toxicity occurs, dose adjustment is unnecessary, and regular examinations will be implemented. For moderate or severe haematological toxicity, drug administration will be stopped immediately, and symptomatic treatment will be provided. If haematological toxicity decreases, drug administration will resume with a reduced dose.

Granulocyte colony-stimulating factor (G-CSF) promotes the differentiation of bone marrow stem cells and the proliferation of granulocytes, and it is currently the preferred drug for the treatment of leukopenia. Patients with thrombocytopenia will be treated with platelet growth-promoting factors, such as recombinant human interleukin-11 (rhIL-II) and recombinant human thrombopoietin (rhTPO). For patients with treatment-induced marked short-term thrombocytopenia, low-dose glucocorticoid treatment can be used. In severe cases, platelet transfusion will be required.

If haematological toxicity occurs during the treatment period, the dose can be adjusted or delayed based on the scheme detailed in Table 6. When the ANC count is

less than 1000/mm^3, G-CSF or granulocyte-macrophage colony-stimulating factor (GM-CSF) can be administered concurrently to maintain the number of blood cells.

Table 6 Dose adjustment scheme for haematological toxicity

Toxicity grade	ANC (cells/mm^3)	PLT count (/mm^3)	Dose adjustment
1	1500 – 1900	75000 – 150000	Treatment will continue without dose adjustment.
2	1000 – < 1500	50000 – < 75000	Treatment will be suspended until ANC > 1500 and PLT count > 75000. Then, the treatment will continue at the original dose.
3	500 – < 1000	25000 – < 50000	Treatment will be suspended until ANC > 1500 and PLT count > 75000. Then, the treatment will continue at the original dose.
4	< 500	< 25000	Treatment will be suspended until ANC > 1500 and PLT count > 75000. Then, the dose will be reduced once, or the treatment will be continued at the original dose together with supportive cytokine therapy.

6.3 Concomitant therapy

During the study period, some drugs can be used concomitantly based on the needs and the usage conditions. When the investigational drug causes an AE that needs to be treated, symptomatic drugs can be provided. When the chemotherapeutic drug causes toxic side effects, such as leukopenia, symptomatic drugs can be provided. Anti-vomiting agents can be used to treat chemotherapy-induced vomiting or given in advance to prevent nausea and vomiting. Symptoms due to other causes can be treated with symptomatic drugs. All concomitantly used drugs will be recorded and described on the CRF.

During the drug administration period, other oncotherapy drugs (including other chemotherapeutic drugs, traditional Chinese medicines with antitumour indications, and thymosin) cannot be used.

6.4 Drug policy

Apatinib (investigational drug): The group A will be given apatinib free of charge until disease progression.

Doxorubicin liposomes: A third cycle is free of charge for every 2 cycles purchased.

Blood genetic testing: This testing is free of charge.

7. Study endpoint evaluation

7.1 Efficacy evaluation

The efficacy measures include PFS, ORR, DCR, and OS. Tumour efficacy will be evaluated based on the RECIST 1.1 criteria.

PFS: PFS will be calculated from the time of randomization to disease progression or death.

ORR and DCR: Tumour imaging will be performed at baseline and every 2 subsequent cycles (8 weeks). If chemotherapy is postponed due to toxicity, the patient will receive an efficacy evaluation once every 8 weeks.

Evaluation of quality of life: The EORTC QLQ-C30 will be used to assess the quality of life of patients at baseline and before every 2 subsequent cycles.

OS: OS will be calculated from the time of randomization to death due to any cause.

7.2 Safety evaluation

7.2.1 Definition

1. AEs:

An AE refers to any adverse medical event that occurs in clinical subjects and does not necessarily have a causal relation to treatment. Therefore, an AE can be any adverse or unintended physical sign (e.g., abnormal laboratory results), symptom, or transient drug-related disease. In addition, whether an AE is related to the investigational medication will be considered.

AEs that occur before and after treatment are both considered AEs. Therefore,

safety monitoring [reporting of AEs or serious adverse events (SAEs)] will be conducted from the start of the enrolment to the end of the study. AEs that occur after the signing of the ICF until the start of the investigational treatment are also considered AEs.

2. ADR:

All toxic and unintentional dose-dependent reactions to drugs will be considered ADRs. A reaction to a drug indicates that there is a rational causal relationship between the drug and an AE; that is, this relationship cannot be excluded.

3. SAEs:

SAEs refer to all lethal or life-threatening adverse medical events that occur under any drug dose. "Serious" and "life-threatening" are defined as follows: when an AE occurs, the subject is at risk of death, rather that assuming that the AE may lead to death if the AE becomes more serious. If an SAE occurs during the study, the investigators will immediately take appropriate protective measures for the subject and report the SAE to the principal investigator within 24 h. The investigator will complete an SAE report form and sign and date the report.

SAEs include the following AEs:

A. Lethal or life-threatening AEs;

B. AEs that cause hospitalization or prolong hospitalization;

C. AEs that cause permanent disability;

D. Carcinogenic AEs; and

E. Disability-causing AEs.

4. Other events that should be treated as SAEs

Drug exposure during pregnancy/lactation – In principle, patients who are pregnant or lactating will be excluded. If pregnancy occurs during the study period, the patient should immediately withdraw from the study and should inform the investigators immediately; the patient will be followed up throughout the pregnancy and postpartum period. Even if the mother and child are completely healthy and do not have any AEs, observations will be recorded. Even if pregnancy is not an SAE, it will be reported using an SAE report form.

5. Events that should not be treated as SAEs:

Disease progression is generally not considered an SAE (but if the symptoms and

signs of disease progression meet the SAE criteria, it will be reported as an SAE).

Death itself is a consequence and not considered an SAE (the major cause of death, i.e., the major AE leading to death, will be recorded and reported as an SAE, and the death will be reported as the consequence of the corresponding AE; if there is no exact cause of death, then death itself can be reported as an SAE).

The death of a subject within 1 month after the initiation of treatment will be reported as an SAE. If a subject dies 1 month after the initiation of treatment and the death is caused by disease progression, then the death will not be reported as an SAE.

In this study, due to the severity of the disease, some conditions identified as SAEs will be excluded from the immediate report:

A. Optional hospitalization and surgical treatment; and

B. Optional inpatient treatment for the purpose of simplifying treatment or research measures.

7.2.2 Recording and evaluating AEs

Medical terms will be used to describe AEs. All AEs will be recorded in the corresponding section of the CRF. In addition, an SAE report form (including the initial or follow-up report) will be completed. All patients who participate in the study will be included in the summary, and the reasons for withdrawal during the treatment period or exclusion from the summary will be explained. Detailed case reports will be written for deaths and SAEs. For deaths, the cause of death will be ascertained, focusing on the relationship between death and the investigational drug. Follow-up of unresolved AEs will continue until they are properly resolved or the condition of the patient is stable.

The following aspects of each AE will be recorded in the CRF.

1. Occurrence time (start time) and recovery time (end time);

2. Severity, which will be evaluated and graded by the investigators based on the definitions in NCI-CTC version 4.0 (see Appendix 3):

Degree I (mild): discomfort caused by the AE does not interfere with daily activities;

Degree II (moderate): discomfort caused by the AE reduces or interferes with daily activities;

Degree III (severe): discomfort caused by the AE makes patients unable to work or perform daily activities;

Degree IV: life-threatening or disability-causing AE; and

Degree V: death;

3. The possible association between the AE and the investigational drug will evaluated based on a five-level classification scale: definitely related, highly likely related, possibly related, possibly unrelated, and unrelated. The first 3 levels indicate that an AE is related to the investigational drug. The incidence of AEs will be calculated by dividing the total number of subjects with AEs among the first 3 levels with the total number of subjects who participate in the safety evaluation;

Table 7-1 Criteria for determining the relationship between an adverse event and the investigational drug

Circumstances (to be met at the same time)	Definitely related	Highly likely related	Possibly related	Possibly unrelated	Unrelated
A reasonable temporal sequence	Yes	Yes	Yes	Yes	No
A known event	Yes	Yes	Yes	No	No
Improvement after stopping exposure	Yes	Yes	Yes or no	Yes or no	No
Reappearance of the event on re-exposure	Yes	?	?	?	No
Another cause of the event is most plausible	No	No	No	Yes	Yes

Note: "Yes" means affirmative, "No" means dissentient, "Yes or No" means difficult to affirm or deny, and "?" means unclear.

4. Measures taken for the investigational drug (none, suspended treatment, reduced dose, delayed treatment, and slowed intravenous infusion) and other measures (none, concomitant medication, required hospitalization or prolonged hospitalization, underwent surgery, delayed chemotherapy, discontinued chemotherapy, and reduced chemotherapy dose);

5. Consequences are defined as follows: recovery with sequelae, recovery without sequelae, partial recovery with no need for further treatment, partial recovery with the need for further treatment, and death. Whether the change in toxicity grade/severity is serious will be recorded as yes or no. If the patient has the same AE several times, the AE will be recorded and re-evaluated at each occurrence;

6. The criteria for determining whether an abnormal objective examination result should be reported as an AE are as follows:

(1) The examination result is related to the accompanying symptoms (and/or);

(2) Other diagnostic tests or treatment measures/surgical interventions are required based on the examination results (and/or);

(3) The examination results lead to a change in the drug dose or withdrawal from the study, and other concomitant medications or other treatments are required (and/or);

(4) The investigator or the sponsor believes that the examination result should be reported as an AE.

The result of an examination that is only for the purpose of confirming an abnormality but does not meet any of the above criteria does not constitute an AE. Any abnormal examination results that are determined to be wrong are not required to be reported as AEs.

7.2.3 Reporting system for SAEs

The reporting period for SAEs will begin from the signing of informed consent by the subjects until 30 calendar days (including 30 days) after the last dose of the investigational drug. If an SAE occurs, regardless of whether it is the initial report or follow-up report, an SAE Report Form for Clinical Studies must be filled out immediately, signed and dated, and faxed within 24 h of the investigator's knowledge of the event to the Drug Safety Group of the Oncology Business Unit of Hengrui Medicine, the clinical research associate, the principal investigator, the lead institution, the ethics review committee of the clinical research organization, the CFDA, and the investigator's regional (provincial or metropolitan) FDA (the form should be sent to the CFDA via EMS).

SAEs that occur beyond 30 days after the last drug administration will generally not be reported unless they are suspected to be related to the investigational drug.

For SAEs, the symptoms, severity, correlation with the investigational drug, time of occurrence, treatment time, measures taken, time and method of follow-up, and outcome will be recorded in detail. If the investigators believe that an SAE is not related to the investigational drug but is potentially related to the study conditions (such as discontinuation of the original treatment or comorbidities), this relationship will be elaborated on the SAE page of the CRF. If the severity of an ongoing SAE or its relationship with the investigational drug changes, a follow-up report for the SAE will

be sent immediately to the sponsor. All SAEs will be followed up until recovery or SD.

7.2.4 SAE reporting procedures

Any SAE that occurs during the clinical treatment period and within 30 days after drug withdrawal will be reported immediately to the clinical research associate of the sponsor institution and the principal investigator and the ethics review committee of the clinical research organization and will be reported within 24 h of the investigator's knowledge of the event to the Department of Drug and Cosmetics Registration and the Department of Safety Supervision of the CFDA and the health administrative department. The relevant contact information is provided in the table below:

Organization	Contact person	Fax/Telephone/Address
Cancer Hospital of the Chinese Academy of Medical Sciences	Institutional Ethics Committee	Telephone/Fax: 010-87788495
CFDA	Division of Drug Research Supervision, Department of Drug and Cosmetics Registration	Address: Building 2, No.26 Compound, Xuanwumen West Street, Xicheng District, Beijing<br>ZIP Code: 100053<br>Telephone: 010-88330732

8. Data management

8.1 Data entry and modification

Based on the principles of good clinical practice (GCP), the investigators will keep all the detailed original documents of the patients and record relevant content related to study progress, medication status, laboratory test data, safety data and efficacy evaluation in the CRF. The recorded data must be complete, timely and clear. The CRF, original documents, and medical records will be clear, detailed, and easy to identify by the personnel participating in the clinical study.

An independent data management organization will be responsible for data entry

and management. The data administrator will use SPSS 20.0 software for data entry and management. To ensure the accuracy of the data, 2 data administrators will independently perform double entry and proofreading. For problematic content in the CRF, the data administrator will record such issues on the Deep Reasoning Question (DRQ) form and send the form to the investigators through the clinical research associate. The investigator will provide answers as soon as possible, and the data administrator use the responses by the investigator to modify, confirm, and enter the data. If necessary, the DRQ form can be sent again.

8.2 Database security

After data review and verification, the statisticians will lock the database. No modification to the data files will be allowed after locking. Errors found after locking will be verified and corrected through statistical analysis software.

8.3 Data storage

Based on the GCP principles in China, the data will be kept for more than 5 years for the investigators.

9. Statistical analysis

9.1 Sample size determination

On the basis of the previously published data, median progression-free survival with PLD alone was approximately 3·0 months.[3,4] We hypothesized that addition of apatinib could improve the median progression-free survival from 3·0 to 5·5 months. Assuming a power of 80%, a significance level of 0·05 (two-sided), and a dropout rate of 20%, it was calculated by R that a total of 126 patients were required to be enrolled over a period of 24 months and followed up for 4 months, with the primary analysis planned when approximate 86 progression events or deaths had occurred.

9.2 Statistical analysis of datasets

1. Intension-to-treat set (ITT)

All patients randomised will be included in the ITT set. The dataset will be used for the analysis of PFS and OS.

2. Modified Intension-to-treat set (mITT)

All randomised patients who received at least 1 dose of investigational drug and have at least 1 post-baseline tumor assessment will be included in the mITT set. The dataset will be used for the efficacy analysis except for PFS and OS.

3. Safety analysis set

All patients enrolled in the study who receive at least 1 dose of the investigational drug and have a safety record after drug administration will be included in the safety analysis set. This dataset will be used for the safety analysis.

9.3 Statistical analysis methods

The ITT population will be used for efficacy data analysis. The safety population will be used for safety data analysis. Safety set will be used for the safety analysis. The continuous data will be summarized bv using descriptive statistics, including mean, standard deviation, median, maximum, and minimum values. Categorical data will be presented as frequencies and percentages. Progression-free survival and overall survival were estimated using the Kaplan-Meier method, and the corresponding 95% CIs were calculated with Brookmeyer Crowley method. Hazard ratios (HRs) were estimated using a stratified Cox proportional hazards model with corresponding 95% CIs. The stratified log-rank test was used to compare the difference in survival between the two groups. For objective response rate and disease control rate, the 95% CIs were calculated using the Clopper-Pearson method, and compared using the stratified Cochran-Mantel-Haenszel test. The randomization stratification factors, including previous platinum-sensitive relapsed (yes vs. no) and platinum-free interval (≤ 3 months vs. 3–6 months), will be included in all the stratified statistical models and tests as strata. Statistical analyses were conducted using SAS® software (version 9.4, SAS Institute Inc, Cary, USA).

10. Ethical norms and quality control

10.1 Informed consent

It is the responsibility of the investigators to explain in detail the purpose, methods, and benefits and potential risks of the study to each subject and then obtain a written ICF signed by each subject. For subjects who cannot sign the ICF, their legal representative must sign the consent form. If a subject and his/her legal representative do not have the ability to read, a notary public will be present during the entire notification process. After the subject and his/her legal representative consent to participate in the study, the notary public will sign the ICF, proving that the content in the ICF has been accurately explained and understood.

The investigators will also explain to the subjects that they have the full right to deny participation in or exit the study at any time for any reason. The CRF in this study is accompanied by an ICF, which must be completed in full. If new safety information changes the risk/benefit evaluation, the content of the ICF will be modified/updated if necessary. If such a modification/update is made, all subjects (including those who have been treated) will be informed of the new information, and a revised ICF will be provided to the subjects to obtain their consent to continue participating in the study.

10.2 Ethical norms and policies and regulations

This study will be conducted in accordance with GCP guidelines and the latest edition of the Declaration of Helsinki. Approval by the ethics review committee of the Chinese Academy of Medical Sciences will be obtained before the start of this study. During the clinical study, modifications to the protocol will be reported to the ethics review committee and filed for the record. The investigators will report research progress and SAEs to the institutional review board. When the study is complete, the investigators will inform the ethics review committee.

10.3 Quality Assurance

To ensure that this study will be conducted in strict accordance with the clinical

research protocol, clinical investigators and clinical sponsors will strictly follow the GCP requirements during the entire clinical study process, follow the experimental procedures and assure the accuracy of the test data and the reliability of research conclusions.

11. References

1. Nishida N, Yano H, Komai K, Nishida T, Kamura T, Kojiro M. Vascular endothelial growth factor C and vascular endothelial growth factor receptor 2 are related closely to the prognosis of patients with ovarian carcinoma. *Cancer* 2004; **101**(6): 1364-74.

2. Poveda AM, Selle F, Hilpert F, et al. Bevacizumab Combined With Weekly Paclitaxel, Pegylated Liposomal Doxorubicin, or Topotecan in Platinum-Resistant Recurrent Ovarian Cancer: Analysis by Chemotherapy Cohort of the Randomized Phase III AURELIA Trial. *J Clin Oncol* 2015; **33**(32): 3836-8.

3. Naumann RW, Coleman RL, Burger RA, et al. PRECEDENT: a randomized phase II trial comparing vintafolide (EC145) and pegylated liposomal doxorubicin (PLD) in combination versus PLD alone in patients with platinum-resistant ovarian cancer. *J Clin Oncol* 2013; **31**(35): 4400-6.

4. Mutch DG, Orlando M, Goss T, et al. Randomized phase III trial of gemcitabine compared with pegylated liposomal doxorubicin in patients with platinum-resistant ovarian cancer. *J Clin Oncol* 2007; **25**(19): 2811-8.

12. The requirements for biological sample collection from the enrolled patients

12.1 Tissue sections

1. Paraffin sections were generated from tumours obtained during the initial surgery involving enrolled patients; paraffin sections of tumours from patients who underwent their initial surgery in other hospitals will be obtained from those hospitals.

Sectioning requirements: A total of 10 5-μm-thick or 5 10-μm-thick unstained tissue sections, of which nucleated cells compose more than 80%, with the local tumour

cell content exceeding 70%, will be stored at room temperature (the paraffin sections of tumours from patients enrolled in other sites will be sent to the Cancer Hospital of the Chinese Academy of Medical Sciences).

12.2 Blood collection requirements

2. Peripheral blood collection time: Peripheral blood samples (10 mL) will be collected a total of 4 times, namely, before the first, third, and fifth cycles of chemotherapy and one month after the end of the sixth cycle of chemotherapy. If a patient withdraws from the study due to disease progression, another blood sample (10 mL) will be collected. For patients receiving maintenance treatment in the group A, blood samples (10 mL) will be collected during follow-up examinations every 2 months. All blood samples will be collected using 10-ml cfDNA sample storage tubes. After blood samples are collected, the tubes will be inverted 10 times and stored at room temperature (blood samples from the patients enrolled in other sites will be sent to the Cancer Hospital of the Chinese Academy of Medical Sciences within 5 days).

12.3 Technical roadmap

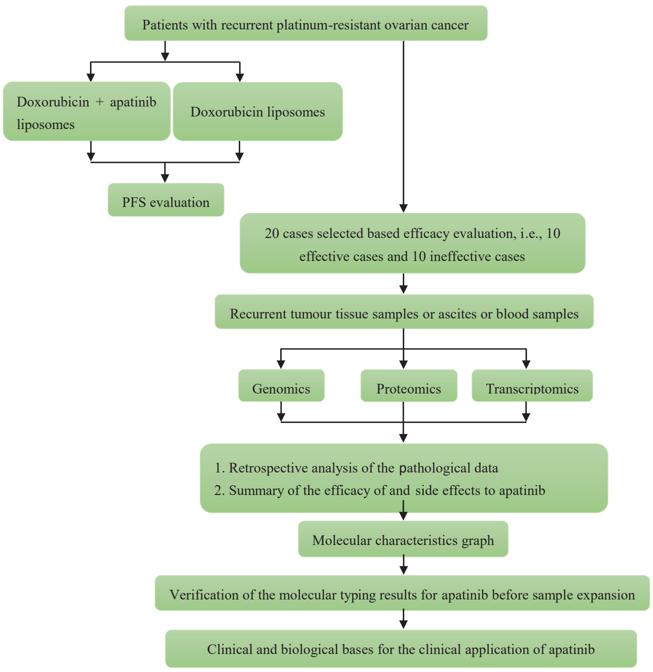

Appendix

Appendix 1. ECOG performance status scoring criteria

Level	Performance status
0	Fully active, capable of all pre-disease performance without restriction
1	Ambulatory and capable of light or sedentary work (light house work or office work) but restricted in physically strenuous activity
2	Ambulatory and capable of all self-care but incapable of any work activities; up and about more than 50% of waking hours
3	Capable of limited self-care; confined to bed or chair more than 50% of waking hours
4	Completely bedridden, incapable of any self-care
5	Dead

Appendix 2. EORTC QLQ-C30 V3.0

We are interested in some things about you and your health. Please answer all of the questions yourself by circling the number that best applies to you. There are no “right” or “wrong” answers. The information that you provide will remain strictly confidential.

Please fill in your code (number):__________

Date of birth:_______year_______month_______day

Today's date: _______year_______month_______day

Not at all A little Quite a bit Very

1. Do you have trouble performing strenuous activities, much

Such as carrying a heavy shopping bag or a suitcase? 1 2 3 4

2. Do you have trouble taking a long walk?

3. Do you have trouble taking a short walk? 1 2 3 4

4. Do you need to stay in bed or a chair during the day? 1 2 3 4

5. Do you need help eating, dressing, washing yourself, or using the 1 2 3 4

toilet? 1 2 3 4

During the past week: Not at all A little Quite a bit Very

6. Were you limited in performing your work or other daily activities? much

7. Were you limited in pursuing your hobbies or other leisure time 1 2 3 4

activities? 1 2 3 4

8. Were you short of breath? 1 2 3 4

9. Have you had pain? 1 2 3 4

10. Did you need to rest? 1 2 3 4

11. Have you had trouble sleeping? 1 2 3 4

12. Have you felt weak? 1 2 3 4

13. Have you lacked an appetite? 1 2 3 4

14. Have you felt nauseated? 1 2 3 4

15. Have you vomited? 1 2 3 4

16. Have you been constipated? 1 2 3 4

During the past week:

17. Have you had diarrhoea? 1 2 3 4

18. Were you tired? 1 2 3 4

19. Did pain interfere with your daily activities? 1 2 3 4

20. Have you had difficulty in concentrating on things, such as reading 1 2 3 4

a newspaper or watching televisions? 1 2 3 4

21. Did you feel tense? 1 2 3 4

22. Did you worry? 1 2 3 4

23. Did you feel irritable? 1 2 3 4

24. Did you feel depressed? 1 2 3 4

25. Have you had difficulty remembering things? 1 2 3 4

26. Has your physical condition or medical treatment interfered with 1 2 3 4

your family life? 1 2 3 4

27. Has your physical condition or treatment interfered with your 1 2 3 4

social activities?

28. Has your physical condition or treatment caused you financial difficulties? 1 2 3 4

For the following questions, please circle the number between 1 and 7 that best applies to you.

29. How would you rate your overall health in the past week?

1 2 3 4 5 6 7

Very poor Very good

30. How would you rate your overall quality of life in the past week?

1 2 3 4 5 6 7

Very poor Very good

Note:

1. Description of quality of life rating

The EORTC QLQ-C30 (V3.0) is a core scale for all cancer patients, with a total of 30 items. Items 29 and 30 are scored on a 1 to 7 scale. The other items are scored on 1 to 4 scale: not at all, a little, quite a bit, and very much, respectively.

2. Calculation of the EORTC QLQ-C30 domain (dimension) scores [raw score (RS)]

For the statistical analysis and interpretation, the scale is often divided into domains. Each domain is a component of quality of life. A domain is also known as a dimension. It is an independent variable in analyses. The 30 items on the EORTC QLQ-C30 (V3.0) are categorised into 15 domains, including 5 function domains (physical, role, cognitive, emotional, and social), 3 symptom domains (fatigue, pain, and nausea/vomiting), 1 general health status/quality of life domain, and 6 single items (each as a domain). The classification is shown in the table below.

The score (RS) for a domain can be obtained by dividing the sum of the scores of all items included in the domain by the total number of items included in the domain, i.e., RS = (Q1+Q2+…+Qn)/n.

EORTC's QLQ-C30 domain classification

	Entry	Item number
Physical functioning	5	1-5
Role functioning	2	6-7
Emotional functioning	4	21-24
Cognitive functioning	2	20-25
Social functioning	2	26-27
General health status	2	29-30
Fatigue	3	10, 12, 18
Nausea/vomiting	2	14-15
Pain	2	9, 19
Shortness of breath	1	8
Insomnia	1	11
Loss of appetite	1	13
Constipation	1	16
Diarrhoea	1	17

Financial difficulties	1	28

3. Calculation of the EORTC QLQ-C30 standard scores

To compare the scores for each domain, the RS must be converted by linear transformation into the standard score (SS) with values ranging from 0-100. In addition, the transformation has another purpose, namely, changing the scoring direction. The items on the QLQ-C30 scale, except for items 29 and 30, are all reverse-scored items (the higher the score, the worse the quality of life), and the scoring rules clearly stipulate that higher scores for function domains and the general health status domain indicate better a functioning status and quality of life and that higher scores for the symptom domains indicate more symptoms or problems (worse quality of life). Therefore, the scoring direction must be changed in the calculation of the SS for the function domains. Specifically, the following equations are used for the calculation (where R is the full range of scores for each domain or item).

Function domains: SS=[1-(RS-1)/R]*100

Symptom domains and general health status domain: SS=[(RS-1)/R]*100

Appendix 3. Response Evaluation Criteria in Solid Tumours (RECIST 1.1) criteria

1 The measurability of the tumour at the baseline level

2.1 Definition:

At the baseline level, tumours/lymph nodes are classified as measurable and unmeasurable based on the following definitions.

2.1.1 Measurable lesions

Tumours: For measurable lesions, there must be at least 1 diameter that can be accurately measured (recorded as the longest diameter). The longest diameter should be **≥ 10 mm on CT (recommended CT slice thickness is ≤ 5 mm),** ≥10 mm on clinical examination using conventional instruments (tumours that cannot be accurately measured by conventional calliper devices will be recorded as unmeasurable), and ≥ 20 mm on chest X-ray. **Malignant lymph nodes are pathologically enlarged and measurable, and the short-axis diameter (SAD) of a single lymph node should be ≥ 15 mm on CT (recommended CT slice thickness is ≤ 5 mm).** At baseline and follow-up, only the SAD will be measured and followed up.

2.1.2 Unmeasurable lesions

All other lesions include small lesions (longest diameter <10 mm or pathological lymph node SAD ≥ 10 mm to < 15 mm) and unmeasurable lesions. Unmeasurable lesions include meningeal disease, ascites, pleural or pericardial effusion, inflammatory breast cancer, cutaneous/pulmonary lymphangitis carcinomatosis, abdominal mass that cannot be diagnosed and followed up by imaging, and cystic lesions.

2.1.3 Special concerns for lesion measurement

Bone lesions, cystic lesions, and lesions that have received local treatment will be noted:

- Bone lesions:
  - Bone scans, positron emission tomography (PET) scans or plain films are not suitable for measuring bone lesions but will be used to confirm the presence or disappearance of bone lesions.

- If osteolytic lesions or mixed osteolytic/osteogenic lesions have a definite soft tissue composition that meets the above definition of measurability and are evaluable using tomographic imaging techniques such as CT or MRI, then these lesions will be considered measurable.
- Osteogenic lesions are unmeasurable lesions.

➢ Cystic lesions:

- Lesions that meet the definition criteria for simple cysts on radiography will not be considered malignant just because they are simple cysts by definition. They are neither measurable lesions nor unmeasurable lesions.
- If a cystic metastatic lesion meets the definition criteria for measurability, it can be regarded as measurable. However, if cystic and noncystic lesions simultaneously present in the same patient, noncystic lesions will be preferentially selected as target lesions.

Locally treated lesions:

- Lesions located at sites that have received radiotherapy or other local treatments will generally be regarded as unmeasurable lesions unless there is clear progression of the lesion. The study protocol will elaborate the conditions under which these lesions are measurable.

1.2 Measurement method description

1.2.1 Measurement of lesions

During clinical evaluations, all tumour measurements will be recorded in the record system. All baseline evaluations of tumour size will be completed before and as close as possible to the start of treatment and **must be completed within 28 days (4 weeks) before the start of treatment.**

1.2.2 Evaluation methods

The same techniques and methods will be used for baseline evaluations and subsequent lesion measurements. Except for lesions that cannot be evaluated by imaging but can only be evaluated by clinical examination, all lesions will be evaluated by imaging.

Clinical lesions: Clinical lesions will only be considered measurable when they are

superficial and have a diameter ≥ 10 mm at the time of measurement (such as skin nodules). For patients with skin lesions, it is recommended to archive colour photographs containing scales to measure lesion sizes. When the lesions are evaluable using both imaging and clinical examinations, imaging will be selected whenever possible because imaging is more objective and can be reviewed repeatedly after the end of the study.

Chest X-ray: When tumour progression is set as a primary endpoint, chest CT is preferred because CT is more sensitive than X-ray, especially for new lesions. Chest X-ray is only applicable when the measured lesion has a clear boundary and the lungs are well ventilated.

CT and MRI: CT is currently the best reproducible method for efficacy evaluations. The measurability in this guideline is defined based on a CT slice thickness ≤ 5 mm. If the CT slice thickness is >5 mm, the minimum measurable lesion will be 2 times the slice thickness. MRI is also acceptable in some cases (e.g., whole-body scan).

Ultrasound: Ultrasound will not be used to measure lesion size. Due to its operational dependence, ultrasound is not repeatable and technical and operational homogeneity cannot be guaranteed among different measurements. New lesions detected by ultrasound during the treatment period will be confirmed by CT or MRI. If CT radiation exposure is a concern, MRI will be used instead.

Endoscopy and laparoscopy: These techniques are not recommended for the objective evaluation of tumours. However, they will be used to obtain biopsy specimens for CR confirmation or for confirmation of recurrence after CR or in surgical resection.

Tumour markers: Tumour markers alone will not be used to evaluate objective tumour response. However, if the baseline level of a tumour marker exceeds the ULN, it will not be used to evaluate CR unless its level returns to normal. The variation in tumour markers in different diseases needs to be taken into account when formulating the measurement criteria into the protocol. Specific criteria for CA-125 (recurrent ovarian cancer) and prostate-specific antigen (recurrent prostate cancer) responses have been published. In addition, the Gynecologic Cancer Intergroup (GCIG) has developed CA-125 progression criteria, which will soon be added to the objective tumour

evaluation criteria of the first-line treatment regimens for ovarian cancer.

Cytological/histological techniques: Under conditions specified in the protocol, these techniques can be used to identify PR and CR (such as the presence of residual benign tumour tissue in germ cell tumours). When exudation may be a potential side effect of a certain therapy (such as treatment with taxane compounds or angiogenesis inhibitors) and a measurable tumour meets the criteria for response or SD, the occurrence or exacerbation of tumour-related exudation during the treatment will be diagnosed by cytological techniques to distinguish between response (or SD) and disease progression.

3 Tumour response evaluation

2.1 Evaluation of all tumours and measurable lesions

To evaluate objective response or possible future progression, it will be necessary to perform a baseline evaluation of the total tumour burden as a reference for the subsequent measurements. In the clinical protocols with objective response as the primary endpoint, only patients with measurable lesions at baseline are eligible. Measurable lesions are defined as the presence of at least one measurable lesion. For trials with disease progression (disease progression time or degree of progression on a fixed date) as the primary endpoint, the inclusion criteria for the protocols will clarify whether only patients with measurable lesions are eligible or patients with no measurable lesions are also eligible.

2.2 Baseline records of target lesions and nontarget lesions

When there is more than 1 measurable lesion during a baseline evaluation, all measurable lesions will be recorded and measured, but the **total number of measurable lesions will not exceed 5 (no more than 2 in each organ)**. For patients with 1 or 2 organs involved, up to 2 or 4 target lesions will be selected for baseline measurements. The target lesions will be selected based on the size (the longest diameter) and will represent all the involved organs, and the measurement will be highly reproducible. If the measurement of the largest lesion is not reproducible, the largest one among reproducible lesions will be selected.

Special attention will be paid to lymph nodes because they are normal tissues and can be detected by imaging even without tumour metastasis. Pathological lymph nodes defined as measurable nodules or even target lesions must meet the following criteria. The CT measurement of the SAD must be ≥ 15 mm. Only the SAD will be measured at baseline. Radiologists usually use the SAD of a nodule to determine whether the nodule has undergone metastasis. The size of a nodule is generally represented by the two-dimensional data from imaging (the axial plane on CT and the axial, sagittal or coronal plane on MRI). The minimum value is the SAD. For example, a 20 mm × 30 mm abdominal nodule has an SAD of 20 mm and can be regarded as malignant and measurable. In this example, 20 mm is the measured value of the nodule. Nodules with a diameter ≥ 10 mm but < 15 mm will not be considered target lesions. Nodules < 10 mm will not be categorized as pathological nodules and will not be recorded and further observed.

The sum of the diameters of all target lesions (including the longest diameter of nonnodular lesions and the SAD of nodular lesions) will be reported as the baseline sum of the diameters. If the diameters of lymph nodes are included, as mentioned above, only the SAD is included. The baseline sum of the diameters will be used as the reference value for the baseline level of the disease.

All other lesions, including pathological lymph nodes, will be considered nontarget lesions and will not need be measured but will be recorded during the baseline evaluation. For example, the lesion will be classified as "existing", "missing" or, in rare cases, "clear progression". Extensive target lesions will be recorded with target organs (e.g., numerous enlarged pelvic lymph nodes or large-scale liver metastasis).

2.3 Criteria for response

2.3.1 Evaluation of target lesions

CR: All target lesions have disappeared, and the SADs of all pathological lymph nodes (including target nodules and nontarget nodules) have reduced to <10 mm.

PR: The sum of the diameters of all target lesions is at least 30% higher than the diameter sum at baseline.

PD: The sum of the diameters of all target lesions is at least 20% higher than the

reference diameter sum, which is the minimum value of the sum of the diameters of all target lesions measured during the entire study period (if the diameter sum at baseline is the smallest, the baseline value is used as the reference). In addition, the absolute value of the diameter sum must have increased by at least 5 mm (**the appearance of 1 or more new lesions is also considered PD**).

SD: SD is a status between PR and PD, where the reduction in the target lesion does not reach PR and the increase in the target lesion does not reach PD. The minimum value of the diameter sum will be used as a reference.

2.3.2 Precautions for target lesion evaluations

Lymph nodes: Even if the size of the lymph nodes identified as target lesions decreases to less than 10 mm, the actual SAD corresponding to the baseline will be recorded for each measurement (consistent with the anatomical plane of the baseline measurement). This means that if a lymph node is the target lesion, even if CR is achieved, it cannot be concluded that the lesion has disappeared completely because the SAD of a normal lymph node is defined as <10 mm. In the CRF or other recording forms, it is necessary to specifically record the target lesion (lymph node) at a specific location: for CR, the SADs of all lymph nodes must be <10 mm; for PR, SD and PD, the measured value of the SAD of the target lymph node will be included in the sum of the diameters of the target lesions.

Target lesions that are too small to be measured: In clinical studies, all lesions (nodules or non-nodules) recorded at baseline should be measured again and recorded, even if the lesions are very small (e.g., 2 mm). However, sometimes a lesion may be too small, resulting in very fuzzy CT images. In this case, the radiologists cannot accurately define the exact size of the lesion and may report it as “too small to be measured”. However, it is very important to record a value on the CRF in this condition. In this trial, even if the radiologist believes that the lesion may have disappeared, he/she will still record 0 mm on the CRF. If the lesion does exist but is too fuzzy to be accurately measured, the default size will be 5 mm (note: this condition is unlikely to occur in lymph nodes because they generally have a measurable size under normal conditions or are often surrounded by adipose tissue as in the retroperitoneal cavity,

whereas if the lymph nodes cannot be measured accurately, the default size will also be 5 mm). The default size of 5 mm is derived from the slice thickness of the CT scan (however, this value does not change with the slice thickness of the CT scan). Because the probability of the repeated occurrence of the same measurement value is small, the use of this default value will reduce the risk of an erroneous evaluation. However, it should be reiterated that if the radiologist can obtain the exact lesion size, even if the diameter of the lesion is less than 5 mm, the actual size will be recorded.

Dissociated or combined lesions: When a nonnodular lesion dissociates into fragments, the sum of the diameters of the fragments will be calculated by adding the longest diameters of all fragments. Similarly, the planes between combined lesions can be used to distinguish each lesion, and then the longest diameter of each lesion can be calculated. However, if the combined lesions fuse into an inseparable lesion, the longest diameter should be the longest diameter of the entire fused lesion.

2.3.3 Evaluation of nontarget lesions

This section defines the response criteria for nontarget tumours. Although some nontarget lesions are measurable, they will not be measured but will be qualitatively evaluated at the time points planned in the protocol.

CR: All nontarget lesions have disappeared, and tumour markers have returned to normal levels; all lymph nodes are of nonpathological size (SAD ≤ 10 mm).

PR/non-PD: The presence of one or more nontarget lesions and/or the persistent presence of abnormally high tumour marker levels.

PD: Clear progression of existing nontarget lesions. **Note: The appearance of one or more new lesions is also considered PD**.

2.3.4 Special considerations for rating the progression of nontarget lesions

The following is a supplementary explanation of the definition of the progression of nontarget lesions: when patients have measurable nontarget lesions, to achieve ‘unequivocal progression’ on the basis of the non-target disease, there must be an overall level of substantial worsening in non-target disease such that, even in presence of SD or PR in target disease, the overall tumour burden has increased sufficiently to merit discontinuation of therapy. However, a general size increase in 1 or more

nontarget lesions often cannot meet the criteria for progression. The designation of overall progression solely on the basis of change in non-target disease in the face of SD or PR of target disease will therefore be extremely rare..

The situation that all nontarget lesions are unmeasurable may occur in some phase III trials, in which the inclusion criteria do not stipulate the necessity of measurable lesions. The overall evaluation is based on the above criteria, but measurable lesion data are absent in this situation. Because worsening in non-target disease cannot be easily quantified (by definition: if all lesions are truly non-measurable) a useful test that can be applied when assessing patients for unequivocal progression is to consider if the increase in overall disease burden based on the change in non-measurable disease is comparable in magnitude to the increase that would be required to declare PD for measurable disease. For example, such progression can be described as an increase in tumour burden equivalent to an additional 73% increase in volume (equivalent to a 20% increase in the diameters of measurable lesions), the change in peritoneal exudation from “minimal” to “massive”, the change in lymphatic lesions from “local” to “extensively expanded”, or “sufficient to change the treatment method” in the protocol. Examples include pleural exudate increasing from a trace amount to a large amount, lymphatic involvement spreading distally from the primary site, or a description of "necessity for treatment changes" in the protocol. In this clinical study, if there is clear progression, the patient will be considered to have disease progression at that time point. It is best to have objective criteria that can be applied to the evaluation of unmeasurable lesions. Note that the supplementary criteria must be reliable.

2.3.5 New lesions

The appearance of new malignant lesions indicates disease progression; therefore, the evaluation of new lesions is critical. Currently, there is no specific standard for the imaging detection of lesions. However, the finding of a new lesion should be unequivocal. For example, progression cannot be attributed to different imaging techniques, changes in imaging morphology, or lesions other than tumours (e.g., some so-called new bone lesions are merely cured original lesions or the recurrence of original lesions). This is particularly important when the patient’s baseline lesions show

partial or complete response. For example, a necrotized liver lesion may be classified as a new cystic lesion on the CT report, although it is not.

Lesions that are detected during the follow-up but were not found in the baseline examination will be considered new lesions and suggestive of disease progression. For example, if a patient with visceral lesions in the baseline examination is found to have metastases during a CT or MRI cranial examination, intracranial metastases will be considered the basis of disease progression even if the patient did not undergo a cranial examination during the baseline examination. If a new lesion is equivocal, for example because of its small size, continued therapy and follow-up evaluation will clarify if it represents truly new disease. If repeat scans confirm there is definitely a new lesion, then progression should be declared using the date of the initial scan.

In addition to fluorodeoxyglucose (FDG)-PET, additional tests are generally required for supplementary confirmation. It is rational to combine FDG-PET and complementary CT results to evaluate progression (especially for new suspected diseases). New lesions can be confirmed by FDG-PET using the following procedures.

Negative baseline FDG-PET results together with the positive FDG-PET results in a subsequent follow-up indicate disease progression.

In cases with no baseline FDG-PET and positive FDG-PET results in a subsequent follow-up, if the new lesion indicated by the positive FDG-PET is confirmed by CT, then disease progression is confirmed; if the new lesion indicated by the positive results of the follow-up FDG-PET is not confirmed by CT, another CT should be performed to confirm the lesion (if confirmed, the disease progression time is counted from the initial discovery of the lesion); if the lesion is confirmed to be an existing lesion recorded in a past CT examination and no progression of the lesion has been found by imaging, then there is no disease progression.

2.4 Evaluation of the best overall efficacy

The best overall efficacy will be evaluated based on the best response from the start of treatment to the end of treatment, and all requisites will be considered for confirmation. Sometimes, the response occurs after the end of treatment. Therefore, the protocol will clarify whether an efficacy evaluation after the end of the treatment is

included in the best overall efficacy evaluation. The protocol will clarify how any new treatment before progression affects the best response. The best response mainly depends on target lesion and nontarget lesion results, as well as the presence of new lesions. In addition, depending on the nature of the study and the protocol requirements, it may also require confirmatory measurement. Specifically, in nonrandomized trials, response is the primary goal, and efficacy, i.e., PR or CR, must be confirmed to determine the best overall efficacy.

2.4.1 Time point response

It is assumed that at each protocol specified time point, a response assessment occurs. Table 1 summarises the overall response of a patient population with measurable disease at baseline at each time point. The evaluation of patients with no measurable lesions (no target lesions) is detailed in Table 2.

2.4.2 Missing assessments and inevaluable designation

When no imaging/measurement is done at all at a particular time point, the patient is not evaluable (NE) at that time point. If only a subset of lesion measurements are made at an assessment, usually the case is also considered NE at that time point, unless a convincing argument can be made that the contribution of the individual missing lesion(s) would not change the assigned time point response. This would be most likely to happen in the case of PD. For example, if a patient had a baseline sum of 50 mm with three measured lesions and at follow-up only two lesions were assessed, but those gave a sum of 80 mm, the patient will have achieved PD status, regardless of the contribution of the missing lesion.

2.4.3 Best overall response

At all time points, once all the patient data are available, the best overall response will be determined.

Evaluation of the best overall response when confirmation of CR or PR is not required: The best treatment response is the best response at all time points (e.g., if the efficacy in a patient is rated SD for the first cycle, PR for the second cycle, and PD for the last cycle, then the best overall response for the patient is PR). When the best overall response is rated SD, the requirement for the minimum interval from baseline to SD

specified in the protocol must be met. If the minimum interval requirement is not met, even if the best overall response is rated SD, the evaluation result will not recognized, and the best overall response of the patient will be determined by a subsequent evaluation. For example, if a patient response is rated SD for the first cycle and PD for the second cycle but does not meet the minimum interval requirement, the best overall response is PD. Similarly, if a patient response is rated SD for the first cycle but the patient becomes lost to follow-up after the first cycle, the best overall response is considered NE.

Evaluation of the best overall response when confirmation of CR or PR is required: If each subject meets the criteria for PR or CR and the efficacy is confirmed at a subsequent time point (usually 4 weeks later) specifically mentioned in the protocol, the best overall response can be declared CR or PR. In this situation, the best overall response is detailed in Table 3.

2.4.4 Special tips for efficacy evaluations

When nodular lesions are included in the total target lesion evaluation and the size of the nodule decreases to the "normal" size (<10 mm), a lesion size scan report will be generated. To avoid the over-evaluation of efficacy based on an increase in nodule size, the measurement results will be recorded even if the nodule is normal. As mentioned earlier, this means that even for subjects with a CR, the nodule size will not be recorded as 0 on the CRF. If the efficacy needs to be confirmed during treatment, repeated "unmeasurable" time points will complicate the evaluation of the best efficacy. The analysis plan must state that these missing data/evaluations will be clearly explained when determining efficacy. For example, in most trials, PR-NE-PR can be regarded as confirmation of efficacy.

When treatment must be discontinued due to the deterioration in the general health status of a participant that is not supported by objective evidence, the efficacy will be reported as symptomatic progression. Even after the discontinuation of treatment, objective progression will be evaluated whenever possible. Symptomatic deterioration is a reason for the discontinuation of treatment but is not an objective response evaluation. The objective response of such subjects will be evaluated based on the

conditions of target and nontarget lesions, as detailed in Tables 1 to 3.

Conditions defined as early progression, early death and NE are special cases and will be clearly described for each regimen (depending on the treatment interval and treatment cycle).

In some circumstances it may be difficult to distinguish residual disease from normal tissue. When the evaluation of complete response depends upon this determination, it is recommended that the residual lesion be investigated (fine needle aspirate/biopsy) before assigning a status of complete response. If abnormal imaging results for local lesions are considered to represent lesion fibrosis or scar formation, FDG-PET can be used as an evaluation standard, similar to biopsy, to confirm CR. In this situation, the application of FDG-PET will be prospectively described in the protocol, and the specialized medical literature reporting this situation will be cited as support. However, limitations of FDG-PET and biopsy (including their resolution and sensitivity) will lead to false positive results in the evaluation of CR.

Table 1 Time point response: patients with target (+/–non-target) disease

Target lesions	Nontarget lesions	New lesions	Overall response
CR	CR	No	CR
CR	non-CR/non-PD	No	PR
CR	Not fully evaluable	No	PR
PR	non-PD or not fully evaluable	No	PR
SD	non-PD or not fully evaluable	No	SD
Not fully evaluable	non-PD	No	NE
PD	Any	Yes or no	PD
Any	PD	Yes or no	PD
Any	Any	Yes	PD
CR = complete response; PR = partial response; SD = stable disease; PD = progressive disease; NE =non-evaluable			

Table 2 Time point response: patients with non-target disease only

Nontarget lesions	New lesions	overall response
CR	No	CR
Non-CR or non-PD	No	Non-CR or non-PD
Not fully evaluable	No	NE

Unconfirmed PD	Yes or no	PD
Any	Yes	PD

Note: For nontarget lesions, "non-CR/non-PD" refers to an efficacy superior to SD. As SD is increasingly used as an endpoint for rating efficacy, non-CR/non-PD has been formulated to target conditions without measurable lesions. For unclear progression findings (such as very small unconfirmed new lesions and cystic degeneration of original lesions or necrotized original lesions), treatment will be continued until the next evaluation. If disease progression is confirmed in the next evaluation, the date of progression will be the date when progression is first suspected.

Table 3 Confirmation of best overall response as CR or PR

Overall response at the first time point	Overall response at the subsequent time point	Best overall response
CR	CR	CR
CR	PR	SD, PD or PR
CR	SD	If the minimum interval requirement for SD is satisfied, then the best overall response is SD; otherwise, it is PD.
CR	PD	If the minimum interval requirement for SD is satisfied, then the best overall response is SD; otherwise, it is PD.
CR	NE	If the minimum interval requirement for SD is satisfied, then the best overall response is SD; otherwise, it is NE.
PR	CR	PR
PR	PR	PR
PR	SD	SD
PR	PD	If the minimum interval requirement for SD is satisfied, then the best overall response is SD; otherwise, it is PD.
PR	NE	If the minimum interval requirement for SD is satisfied, then the best overall response is SD; otherwise, it is NE.
NE	NE	NE

Note: CR is complete response, PR is partial response, SD is stable disease, PD is progressive disease, and NE is non-evaluable. Superscript "a": If CR truly occurs at the first time point and any disease occurs at the subsequent time point, then treatment efficacy at the subsequent time point is still rated PD (because the disease will occur after a CR) even if the efficacy meets the PR criteria relative to baseline. The best response depends on whether SD occurs within the minimum treatment interval. Sometimes, although the evaluation at the first time point is CR, scans at subsequent

time points suggest small lesions; therefore, the treatment efficacy for the subject at the first time point should be PR rather than CR. In this situation, the first judgement of CR should be modified to PR, and the best response is PR.

2.5 Frequency of tumour re-evaluations

The frequency of tumour re-evaluations during treatment will be determined by the treatment regimens and will occur in accordance with the type of and schedule for treatment. However, in the phase II trial in which the benefit of treatment was not clear, it is reasonable to conduct follow-up every 6 to 8 weeks (at the end of a cycle), and the length of the time interval will be adjusted under special schemes or circumstances. The protocol will specifically indicate which tissue sites need to be evaluated at baseline (usually those tissue sites that are most likely to be closely related to the metastatic lesions of the tumour type under study) and the frequency of re-evaluation. Under normal circumstances, target lesions and nontarget lesions will be rated at each evaluation. In some optional cases, the evaluation frequency of some nontarget lesions will be lower; for example, bone scans should be repeated only when the treatment efficacy for the target disease is confirmed to be CR or when bone lesion progression is suspected.

After the end of treatment, the re-evaluation of tumours depends on whether the response rate or the occurrence of a certain event (progression/death) is used as the clinical trial endpoint. If the clinical trial endpoint is the occurrence of a certain event (e.g., TTP/DFS 1/PFS), routine re-evaluations, as specified in the protocol, are required. In randomized comparative trials, the scheduled evaluations should be listed in the study schedule (e.g., 6-8 weeks of treatment or 3-4 months after the treatment) and should not be affected by other factors, such as treatment delays, dosing intervals, and any other events that may cause an imbalance in the treatment arm regarding the selection of disease evaluation time.

2.6 Efficacy evaluation/response confirmation

2.6.1 Confirmation

For nonrandomized clinical trials with efficacy as the primary endpoint, PR and

CR must be confirmed to ensure that the efficacy is not the result of an erroneous evaluation. Confirmation also allows a reasonable interpretation of the results in the presence of historical data, and efficacy in the historical data of these trials should also be confirmed. However, in all other cases, such as randomized trials (Phase II or III) or studies with SD or PD as the primary endpoint, efficacy confirmation is no longer necessary because it is of no value for the interpretation of the experimental results. However, elimination of the requirement for response confirmation may increase the importance of central review to protect against bias, in particular in studies which are not blinded. In the case of SD, at least 1 measurement must meet the SD criteria specified in the protocol within the minimum time interval after the start of treatment (generally no less than 6-8 weeks).

2.6.2 Total response period

The total response period is the interval from the time when the CR or PR (whichever is measured first) criteria are first met to the time of the first actual recording of disease recurrence or progression (the minimum measured value recorded during treatment is used as the reference for disease progression). The total CR period is the interval from the time when the CR criteria are first met to the time of the first actual recording of disease recurrence or progression.

2.6.3 SD period

The SD period is the interval from the start of treatment to disease progression (in a randomized trial, from the time of randomization), and the smallest sum during treatment is used as the reference (if the baseline sum is the smallest, it is used as the reference for PD calculations). The clinical relevance of SD varies in different studies and different diseases. If the proportion of patients achieving stable disease for a minimum period of time is an endpoint of importance in a particular trial, the protocol should specify the minimal time interval required between two measurements for determination of stable disease. Note: The response period, SD period, and PFS are affected by the follow-up frequency after the baseline evaluation. Defining the standard follow-up frequency is outside the scope of this guideline. The frequency of follow-up should consider many factors, such as disease type and stage, treatment cycle and

standard specifications. However, if intertrial comparisons are required, the limitations of the accuracy of these measurement endpoints should be considered.

Appendix 4. 2014 FIGO staging for ovarian cancer, fallopian tube cancer, and peritoneal cancer

I		Tumour confined to the ovaries or fallopian tube(s)
	IA	Tumour limited to 1 ovary (capsule intact) or fallopian tube, no tumour on the ovarian or fallopian tube surface, and no malignant cells in the ascites or peritoneal washings
	IB	Tumour limited to both ovaries (capsule intact) or fallopian tubes, no tumour on the ovarian or fallopian tube surface, and no malignant cells in the ascites or peritoneal washings
	IC	Tumour limited to one or both ovaries or fallopian tubes, with any of the following:<br>IC1 Surgical spill intraoperatively;<br>IC2 Capsule ruptured before surgery or tumour on the ovarian or fallopian tube surface; or<br>IC3 Malignant cells in the ascites or peritoneal washings
II		Tumour involves 1 or both ovaries or fallopian tubes with pelvic extension (below the pelvic brim) or peritoneal cancer (Tp)
	IIA	Extension and/or implants on the uterus and/or fallopian tubes and/or ovaries
	IIB	Extension to other pelvic intraperitoneal tissues
III		Tumour involves 1 or both ovaries or fallopian tubes or primary peritoneal cancer with cytologically or histologically confirmed spread to the peritoneum outside the pelvis and (or) metastasis to the retroperitoneal lymph nodes
	IIIA	Metastasis to retroperitoneal lymph nodes with or without microscopic peritoneal involvement beyond the pelvis<br>IIIA1 Positive retroperitoneal lymph node only (cytologically or histologically confirmed)<br>IIIA1(i) Metastasis ≤ 10 mm in greatest dimension (note: this is the tumour dimension and not the lymph node dimension)<br>IIIA1(ii) Metastasis >10 mm in the greatest dimension<br>IIIA2 Microscopic extrapelvic (above the pelvic brim), peritoneal involvement with or without positive retroperitoneal lymph nodes
	IIIB	Macroscopic peritoneal metastasis beyond the pelvic brim ≤ 2 cm in greatest dimension, with or without positive retroperitoneal lymph nodes
	IIIC	Macroscopic peritoneal metastasis beyond the pelvic brim > 2 cm in greatest dimension, with or without positive retroperitoneal lymph nodes (Note 1)

IV		Distant metastasis excluding peritoneal metastasis
	IVA	Pleural effusion with positive cytology
	IVB	Metastasis to extra-abdominal organs (including inguinal lymph nodes and lymph nodes outside of abdominal cavity) (Note 2)

Note 1: Includes extension of the tumour to the capsule of the liver and spleen without parenchymal involvement of either organ.

Note 2: Parenchymal metastases are stage IVB.

Appendix 5. New York Heart Association (NYHA) classification

Grade I	No limitation in physical activity; ordinary physical activity does not cause undue fatigue, dyspnoea, or palpitations, namely, cardiac compensation
Grade II	Slight limitation in physical activity; ordinary physical activity causes fatigue, dyspnoea, palpitations, or angina, also known as grade I or mild heart failure
Grade III	Marked limitation in physical activity; less than ordinary physical activity leads to the above symptoms, also known as grade II or moderate heart failure
Grade IV	Unable to carry out any physical activity; symptoms of congestive heart failure or angina occur at rest and aggravate after any physical activity, also known as grade III or severe heart failure

Amendment History

Amendment 1: 25 May 2017 (version 1.0-1.1)

- The dosage of apatinib was adjusted from 500 mg q.d. to 250 mg q.d.

Amendment 2: 11 December 2018 (version 1.1-2.0)

- Stratification factors were revised to include platinum-free interval (≤3 months *vs*. 3–6 months) and prior platinum-sensitive relapse (yes *vs*. no).
- Inclusion criteria were revised to previous pathological diagnosis of ovarian cancer, fallopian tube cancer, or primary peritoneal cancer, of which the pathological type is non-mucinous adenocarcinoma, with available paraffin sections from a previous surgery.
- Inclusion criteria were revised to patients without pleural effusion or ascites confirmed by computed tomography or magnetic resonance imaging are required to have at least 1 measurable lesion as the target lesion based on the Response Evaluation Criteria in Solid Tumours (RECIST 1.1) criteria; if the target lesion is a lymph node, the diameter must be greater than 1.5 cm, and it must be unsuitable for surgical treatment; and the target lesion must be free of radiotherapy or relapsed within the radiotherapy field.
- Exclusion criteria of “Refractory patients progressing during previous treatment” was deleted.
- Study plan were revised to enrolment began in March 2018 and is expected to last for 2 years.

Amendment 3: 31 January 2020 (version 2.0-3.0)

- Exclusion criteria were revised to History of local radiation therapy of the pelvis or lower abdomen.
- Drug policy were revised to Apatinib (investigational drug): The group A will be given apatinib free of charge until disease progression. Doxorubicin liposomes: A third cycle is free of charge for every 2 cycles purchased. Blood genetic testing: This testing is free of charge.
