# Supplementary material for: Effect of Apatinib Plus Pegylated Liposomal Doxorubicin vs Pegylated Liposomal Doxorubicin Alone on Platinum-Resistant Recurrent Ovarian Cancer: The APPROVE Randomized Clinical Trial
Source: JAMA Oncol. 2022 Jun 30;8(8):1169–76. doi: 10.1001/jamaoncol.2022.2253 (PMC9247861; doi:10.1001/jamaoncol.2022.2253)
Supplement: Supplement 2. — Statistical analysis plan [file jamaoncol-e222253-s002.pdf]

STATISTICAL ANALYSIS PLAN
(SAP)

APATINIB COMBINED WITH STANDARD CHEMOTHERAPY FOR PLATINUM-RESISTANT RECURRENT OVARIAN CANCER: THE APPROVE STUDY

OC-II-002

VERSION 1.0, 06 FEB 2020

CONFIDENTIALITY STATEMENT

The information is provided to you in confidence to enable you to perform the work. Do not give this document or any copy of it or reveal any proprietary information contained in it to any third party or person without the prior permission of an authorization.

APPROVAL FORM

**Prepared By:**		
Wenqing Zhu/Statistician		
**Print Name/Role**	**Signature**	**Date**
**Approved By:**		
Fenglin She/Medical Lead		
**Print Name/Role**	**Signature**	**Date**
Ni Guan/Statistician		
**Print Name**	**Signature**	**Date**

DOCUMENT REVISION HISTORY		
**Document Version**	**Document Date**	**Reason for Revision**
1.0	06FEB2020	First version – Not applicable

TABLE OF CONTENTS

APPROVAL FORM 2
LIST OF ABBREVIATIONS AND DEFINITIONS OF TERMS 7
1. INTRODUCTION 9
2. STUDY OBJECTIVES 10
2.1. Primary Objectives 10
2.2. Secondary Objectives 10
3. STUDY DESIGN AND METHODS 11
3.1. General Study Design and Plan 11
3.2. Randomization 11
3.3. Blinding 11
4. STUDY ENDPOINTS 12
4.1. Efficacy Endpoint(s) 12
4.1.1. Primary Efficacy Endpoint(s) 12
4.1.2. Secondary Efficacy Endpoint(s) 12
4.2. Safety Endpoint(s) 12
4.3. Health Economics and Outcome Research Endpoint(s) 12
5. SAMPLE SIZE DETERMINATION 13
6. INTERIM ANALYSIS 14
7. GENERAL AND STATISTICAL CONSIDERATIONS 15
7.1. Analysis Sets 15
7.1.1. Intention-To-Treat Set 15
7.1.2. Modified Intention-To-Treat Set 15
7.1.3. Safety Analysis Set 15
7.2. General Considerations 15
7.2.1. Reference Start Date, End Date and Study Day 15
7.2.2. Baseline 15
7.2.3. Definition and Use of Visit Windows 15
7.2.4. Repeated or Unscheduled Assessments of Safety Parameters 16

7.3. Statistical Considerations 16
7.3.1. Missing Date or Incomplete Date 16
7.3.1.1. Missing or Incomplete Date Information of Study Treatment 16
7.3.1.2. Missing or Incomplete Date Information for New Antitumor Therapies 16
7.3.1.3. Missing or Incomplete Date Information for the First Pathology Diagnosis 17
7.3.1.3. Missing or Incomplete Date Information for Adverse Events and Concomitant Medications 17
7.3.1.4. Missing or Incomplete death date 18
7.3.1.5. Missing or Incomplete PD date 18
7.3.2. Character Values of Clinical Laboratory Tests 18
7.3.3. Computing Methods and Reporting Conventions 18
7.3.3.1. Statistical Summary Conventions 18
7.3.3.2. General Reporting Conventions 19
7.3.4. Subgroups 19
8. STATISTICAL ANALYSIS 20
8.1. Summary of Study Data 20
8.1.1. Subject Disposition 20
8.1.2. Protocol Deviations 20
8.1.3. Demographic and Baseline Characteristics 20
8.1.4. Tumor Diagnosis 21
8.1.5. Tumor Treatment History 21
8.1.6. Medical History 22
8.1.7. Concomitant Medications 22
8.2. Efficacy Analyses 23
8.2.1. Analysis of Primary Efficacy Endpoints 23
8.2.1.1. Main analysis of the primary efficacy endpoint 24
8.2.1.2. Sensitivity analysis of the primary efficacy endpoint 24
8.2.1.3. Subgroup analysis 25
8.2.2. Analysis of Secondary Efficacy Endpoints 25
8.2.2.1. Overall survival 25

8.2.2.2. Objective response rate (ORR) and Disease control rate (DCR) 26
8.2.2.3. Duration of Response (DoR) and Time to Response (TTR) 26
8.3. Safety Analyses 27
8.3.1. Dosing and Extent of Exposure 27
8.3.2. Adverse Events 28
8.3.2.1. Treatment-related TEAEs 29
8.3.2.2. Serious adverse events 30
8.3.2.3. TEAE leading to study drug interruption, dose reduction and discontinuation 30
8.3.2.4. Death, Other Serious Adverse Events and Other Significant Adverse Events 30
8.3.3. Clinical Laboratory Evaluations 30
8.3.4. Vital Sign 31
8.3.5. ECG and Echocardiogram 31
8.3.6. Eastern Cooperative Oncology Group (ECOG) PS 31
8.4. Patients Reported Outcome (PRO) Analysis 31
9. SUMMARY OF CHANGES TO THE STATISTICAL ANALYSES SPECIFIED IN PROTOCOL 33
10. REFERENCES 34
11. APPENDICES 35

LIST OF ABBREVIATIONS AND DEFINITIONS OF TERMS

ABBREVIATION	DEFINITION
AE	Adverse event
ALT	Alanine aminotransferase
ANC	Absolute neutrophil count
AST	Aspartate aminotransferase
ATC	Anatomical therapeutic chemical class
BLQ	Below level of quantification
BMI	Body mass index
BOR	Best overall response
BSA	Body surface area
BUN	Blood urea nitrogen
CR	Complete response
DCR	Disease control rate
DoR	Duration of response
ECG	Electrocardiogram
ECHO	Echocardiogram
ECOG	Eastern cooperative oncology group
eCRF	Electronic case report form
EORTC QLQ-C30	European Organization for Research and Treatment of Cancer Quality of Life Questionnaire-Core 30
EOS	End of study
HRQoL	Health-related quality of life
ITT	Intention-to-treat
LVEF	Left ventricular ejection fraction
MedDRA	Medical Dictionary for Regulatory Activities
NE	Not evaluable
ORR	Objective response rate
OS	Overall survival
PD	Progression disease
PFS	Progression-free survival
PLD	Pegylated liposomal doxorubicin
PLT	Platelet count
PR	Partial response
PRO	Patient reported outcome
PS	Performance status
PT	Preferred term
QoL	Quality of life
QTc	Q-T interval corrected for heart rate

ABBREVIATION	DEFINITION
RECIST	Response Evaluation Criteria in Solid Tumors
RS	Raw score
SAE	Serious adverse events
SAP	Statistical analysis plan
SAS	Statistical Analysis System
SD	Stable disease
SOC	System organ class
SS	Survival analysis set
TBIL	Total bilirubin
TEAE	Treatment-emergent adverse event
TRAE	Treatment-related TEAE
TTR	Time to response
WBC	White blood cells

1. INTRODUCTION

This statistical analysis plan (SAP) provides a detailed, technical elaboration of the statistical analyses of efficacy and safety data as described in the study protocol Version 3.0 dated 31-JAN-2020. Specifications for tables, listings and figures are contained in a separate document.

2. STUDY OBJECTIVES

2.1. Primary Objectives

- To assess the efficacy of apatinib plus PLD compared with PLD in platinum-resistant ovarian cancer, as measured by progression-free survival (PFS) according to Response Evaluation Criteria in Solid Tumors (RECIST) version 1.1.

2.2. Secondary Objectives

- To assess the overall survival (OS) in subjects treated with apatinib plus PLD compared with PLD.
- To assess the objective response rate (ORR) in subjects treated with apatinib plus PLD compared with PLD.
- To assess the disease control rate (DCR) in subjects treated with apatinib plus PLD compared with PLD .
- To assess the duration of response (DoR) in subjects treated with apatinib plus PLD compared with PLD.
- To assess the time to response (TTR) in subjects treated with apatinib plus PLD compared with PLD.
- To assess the safety and tolerability of apatinib plus PLD compared with PLD.

3. STUDY DESIGN AND METHODS

3.1. General Study Design and Plan

This is a multicentre, open-label, randomized, parallel controlled, clinical trial to evaluate the efficacy and safety of apatinib plus PLD in treating platinum-resistant ovarian cancer.

Eligible subjects will be randomized at a ratio of 1:1 to receive either PLD alone or apatinib plus PLD, with the stratification by platinum-free interval (≤3 months *vs.* 3–6 months [excluding the boundary values] from last receipt of platinum-based chemotherapy to progression) and prior platinum-sensitive relapse (yes *vs.* no).

- Subjects assigned to PLD group will receive 40 mg/m² doxorubicin hydrochloride liposomes intravenously on day 1, every 28 days is a treatment cycle.
- Subjects assigned to apatinib plus PLD group will receive 250 mg apatinib orally once a day plus 40 mg/m² doxorubicin hydrochloride liposomes intravenously on day 1, every 28 days is a treatment cycle.

Subjects will receive study treatment with each cycle of 28 days until the occurrence of progressive disease (PD), death, intolerable toxicity, withdrawal of consent, or investigator's decision.

Efficacy evaluation will be performed every 8 weeks.

3.2. Randomization

The eligible subjects will be randomly assigned to receive either PLD alone or apatinib plus PLD at a ratio of 1:1 via an interactive web response system based on a sequence generated with blocks and stratified by platinum-free interval (≤3 months *vs.* 3–6 months [excluding the boundary values] from last receipt of platinum-based chemotherapy to progression) for the first 13 patients, and platinum-free interval (≤3 months *vs.* 3–6 months [excluding the boundary values] and prior platinum-sensitive relapse (yes *vs.* no) for the remaining patients. Patients and investigators will not be masked to the treatment assignment.

3.3. Blinding

Not applicable. This is an open-label study.

4. STUDY ENDPOINTS

4.1. Efficacy Endpoint(s)

4.1.1. Primary Efficacy Endpoint(s)

- Investigator assessed PFS based on RECIST v1.1: defined as the time from randomization to objective disease progression or any cause of death, whichever occurs first.

4.1.2. Secondary Efficacy Endpoint(s)

- Overall Survival (OS): defined as the time from randomization to death of any cause.
- Objective response rate (ORR): defined as the proportion of subjects whose best overall response (BOR) is CR or PR, evaluated by investigator based on RECIST v1.1.
- Disease Control Rate (DCR): defined as the proportion of subjects whose best overall response (BOR) is complete response (CR), partial response (PR) and stable disease (SD), evaluated by investigator based on RECIST v1.1.
- Duration of response (DoR): defined as the time from first CR/PR, evaluated by investigator based on RECIST v1.1, to PD or any cause of death, whichever occurs first.
- Time to Response (TTR): defined as the time from randomization to first CR/PR, evaluated by investigator based on RECIST v1.1.

4.2. Safety Endpoint(s)

Safety endpoints include TEAE, clinical laboratory test parameters, vital sign, electrocardiogram, echocardiogram and Eastern Cooperative Oncology Group (ECOG) performance status (PS).

4.3. Health Economics and Outcome Research Endpoint(s)

- Quality of Life (EORTC QLQ-C30)

5. SAMPLE SIZE DETERMINATION

The primary endpoint is PFS in subjects with platinum-resistant recurrent ovarian cancer treated with apatinib plus PLD. A total of 126 subjects will be randomized using a 1:1 ratio to receive either apatinib plus PLD or PLD alone.

Referring to previously published data, assuming a median PFS of 3.0 months in control group (PLD alone) and a median PFS of 5.5 months in treatment group (apatinib plus PLD), it will require 86 PFS events to provide 80% power to detect a difference in PFS between groups using a log-rank test at a two-sided significance level (α)=0.05. Assuming an enrollment period of 24 months and a follow-up of 4 months, 50 subjects in each group will be required to observe sufficient PFS events at the end of study. By considering a drop-out rate of 20%, 63 subjects in each group is needed and the total sample size is 126 subjects.

6. INTERIM ANALYSIS

Not applicable. No interim analyses were planned.

7. GENERAL AND STATISTICAL CONSIDERATIONS

7.1. Analysis Sets

7.1.1. Intention-To-Treat Set

The Intention-To-Treat Set (ITT) will include all randomized subjects. ITT will be used for the analysis of PFS and OS.

7.1.2. Modified Intention-To-Treat Set

The modified Intention-To-Treat Set (mITT) will include all randomized subjects who received at least 1 dose of study drug and have at least 1 post-baseline tumor assessment. mITT will be used for the supportive analyses except for PFS and OS as well as the main analyses of ORR and DCR.

7.1.3. Safety Analysis Set

The Safety Analysis Set (SS) will include all subjects who received at least 1 dose of study drug and have a safety record after drug administration. SS will be used for the safety analyses.

7.2. General Considerations

7.2.1. Reference Start Date, End Date and Study Day

- Reference start date is the first date of study treatment administration
- Reference end date is the last date of study treatment administration
- Study day = event/assessment date – reference start date + (event/assessment date ≥ reference start date)

7.2.2. Baseline

In general, baseline is defined as the last non-missing measurement taken prior to reference start date. In the case where the last non-missing measurement and the reference start date coincide, that measurement will be considered pre-baseline, but Adverse Events (AEs) and medications commencing on the reference start date will be considered post-baseline.

7.2.3. Definition and Use of Visit Windows

All the by-visit analysis will use the original visits being recorded in the eCRF. No derivation of visit will be performed.

7.2.4. Repeated or Unscheduled Assessments of Safety Parameters

In general, all the by-visit summaries will only present the data being recorded in scheduled visits. Measurements recorded in unscheduled visits will not be included in the by-visit summaries, but will contribute to determine the best/worst case value if required (i.e. shift table).

If there are multiple values for the same test before the initiation of study treatment, the last available assessment will be used.

Listings will include all the scheduled and unscheduled data.

7.3. Statistical Considerations

7.3.1. Missing Date or Incomplete Date

In general, the incomplete date will be imputed following the below rule unless otherwise specified if it has impact on the statistical analysis:

- Will not impute if completely missing.

7.3.1.1. Missing or Incomplete Date Information of Study Treatment

7.3.1.1.1. Missing or Incomplete Start Dates

- Only day is missing: impute 1 to the day part.
- Month and day are missing: impute to Jan 01.
- Completely missing date will not be imputed.

7.3.1.1.2. Missing or Incomplete End Dates

- Only day is missing: impute to the last day of the month.
- Month and day are missing: impute to Dec 31.
- Completely missing date: impute to the data cutoff date.

In case the imputed start date is later than the stop date, then the start date will be imputed using the end date.

7.3.1.2. Missing or Incomplete Date Information for New Antitumor Therapies

- Only day is missing: impute 1 to the day part.
- Month and day are missing: impute to Jan 01.
- Completely missing date will not be imputed.

In case the imputed new antitumor therapy date is earlier than the next day of the last date of study treatment administration, then the new antitumor therapy date will be imputed using the next day of the last date of study treatment administration.

7.3.1.3. Missing or Incomplete Date Information for the First Pathology Diagnosis

- Only day is missing: impute 1 to the day part.
- Month and day are missing: impute to Jan 01.
- Completely missing date will not be imputed.

Imputed date should not be earlier than the birth date.

7.3.1.3. Missing or Incomplete Date Information for Adverse Events and Concomitant Medications

7.3.1.3.2. Missing or Incomplete Start Dates

- Missing Day Only
  - If the known parts of the incomplete date is the same as the corresponding parts of the first date of study treatment administration, then impute to the date of first study treatment administration.
  - Otherwise impute 1 to the day part.
- Missing Month and Day
  - If the year of the incomplete date is the same as the year of the first date of study treatment administration, then impute to the date of first study treatment administration.
  - Otherwise impute to Jan 1.
- Completely missing date will not be imputed.

7.3.1.3.3. Missing or Incomplete End Dates

- Only day is missing: impute to the last day of the month. If the imputed date is later than the death date, then use the death date to impute.
- Month and day are missing: impute to Dec 31. If the imputed date is later than the death date, then use the death date to impute.
- Completely missing date will not be imputed.

In case the imputed start date is later than the end date, then the start date will be imputed using the end date.

7.3.1.4. Missing or Incomplete death date

- Only day is missing: impute 1 to the day part.
- Month and day are missing: impute to Jan 01.
- If completely missing: impute to the next day of last date known to be alive.

In case the imputed date is earlier than the next day of last date known to be alive, then the death date will be imputed using the next day of last date known to be alive.

7.3.1.5. Missing or Incomplete PD date

- Missing Day Only
  - If the known parts of the incomplete date is the same as the corresponding parts of the last non-PD tumor assessment date, then impute to the next day of the last non-PD tumor assessment date.
  - Otherwise impute 1 to the day part.
- Missing Month and Day
  - If the year of the incomplete date is the same as the year of the last non-PD tumor assessment date, then impute to the next day of the last non-PD tumor assessment date.
  - Otherwise impute to Jan 1.
- Completely missing date will not be imputed, the subjects will be considered to be censored.

In case the imputed PD date is later than the death date, then the PD date will be imputed using the death date.

7.3.2. Character Values of Clinical Laboratory Tests

If the reported value for a clinical laboratory test is in form of a character string, then the numeric part of the string will be used for the analysis purpose.

If the result is reported as below-limit-of quantification (BLQ), then the lower boundary of the quantifiable assessment range will be used for the analysis purpose. However, the actual values as reported in the database will be presented in data listings.

7.3.3. Computing Methods and Reporting Conventions

All the statistical analysis will be performed with SAS® Version 9.4 or above.

7.3.3.1. Statistical Summary Conventions

For continuous variables, descriptive statistics including number of subjects with non-missing values, mean, standard deviation, median, minimum and maximum will be presented. For categorical variables, frequencies and percentages will be presented. Time-to-events data will be analyzed by Kaplan-Meier method and K-M plots will be presented if necessary.

7.3.3.2. General Reporting Conventions

The means and medians should have 1 more decimal place than the observed values, the standard deviations should have 2 additional decimal place than the observed values. Min and Max should have the same decimal place with the observed values.

p-values will be reported to 4 decimal places, the p-values less than 0.0001 will be reported as "<0.0001", while the p-values greater than "0.9999" will be reported as ">0.9999".

7.3.4. Subgroups

Subgroup analysis will be conducted for PFS and OS, which will be described in corresponding sections. It should be noted that the study is not designed to detect the difference in treatment within subgroups. If the subject number of the group is less than 10 or with other considerations, it maybe pooled with others.

The following subgroups will be assessed for PFS and OS:

- Age as categorical variable (< 65 years, ≥ 65 years)
- ECOG performance status (0, 1–2)
- Previous platinum-sensitive relapse (yes, no)
- Previous therapy lines (1, 2, ≥ 3)
- Platinum-free interval (≤ 3 months, > 3 months)
- Ascites (yes, no)
- Platinum-refractory (yes, no)
- First platinum-resistant relapse (yes, no)

8. STATISTICAL ANALYSIS

8.1. Summary of Study Data

8.1.1. Subject Disposition

The number and percentage of subject in each analysis set (Intention-to-treat set, modified intention-to-treat set and safety analysis set) will be summarized by treatment group. The number and percentage of subjects who discontinue the study treatment and subjects who are still on treatment at the time of analysis will be presented for each treatment group. Reasons for treatment discontinuation will be further summarized as number of subjects and percentage for each treatment group. In addition, the duration of subject in study will also be summarized.

- Duration in study (months) = (Last day of known to be alive – randomization date + 1)/30.4375

Data listing of analysis sets, as well as disposition will be provided.

8.1.2. Protocol Deviations

Protocol deviations will be summarized by the treatment group and overall and furthermore summarized by severity.

Protocol deviations will be listed.

8.1.3. Demographic and Baseline Characteristics

Demographic and baseline characteristics will be summarized by treatment group and overall for ITT using the following information from the ‘Demographic Characteristic’ eCRF pages.

- Age as continuous variable
- Age as categorical variable (<65-year, ≥65-year)
- Gender (male, female)
- Baseline ECOG PS (0, 1, 2)
- Baseline height (cm)
- Baseline weight (kg)
- Baseline Body Mass Index (BMI) (kg/m^2)
- Body Surface Area (BSA) (m^2)

The derivations of BMI and BSA are listed as below:

- BMI (kg/m^2) = weight (kg)/height $(m)^2$

- BSA (m^2) = 0.00586* height (m)+0.0126* weight (kg)-0.0461

The continuous data will be summarized by using descriptive statistics, including mean, standard deviation, median, maximum and minimum values. Categorical data will be presented as frequencies and percentages.

Data listing for demographic information will be provided.

8.1.4. Tumor Diagnosis and treatment history

The tumor diagnosis and prior treatment information listed below will be summarized by treatment group and overall for ITT using the following information from the 'Tumor History' eCRF pages.

- Time since initial diagnosis to date of randomization (months), defined as (date of randomization- date of initial diagnosis)/ 30.4375
- Method of diagnosis (Surgery, Biopsy)
- Ovarian cancer type (Serous carcinoma, Non-serous carcinoma)
- Serous carcinoma histologic subtype (High grade, Low grade)
- Non-serous carcinoma histologic subtype (Well differentiated, Moderately differentiated, Poorly differentiated)
- Number of organs involved in metastasis (≤2, >2)
- Ascites (yes, no)
- Platinum-refractory (yes, no)
- Platinum-free interval (months) (≤3, 3-6)
- Previous antiangiogenic therapy (yes, no)
- Previous PARP inhibitor therapy (yes, no)
- Previous platinum-sensitive relapse (yes, no)
- First platinum-resistant relapse (yes, no)

Data listing for tumor diagnosis will be provided.

8.1.5. Tumor Treatment History

Tumor treatment history will be summarized by treatment group and overall for ITT using the following information from the 'Past History' eCRF pages.

The number and percentage of patients in each of the following tumor treatment history categories will be tabulated:

- Patients with at least one prior chemotherapy history

- Patients with at least one prior anti-cancer radiotherapy
- Patients with at least one prior anti-cancer surgery

Prior chemotherapy history will be summarized as follows based on the number and percentage of patients with the following:

- At least one prior chemotherapy
- Previous treatment lines. If there are patients received multiple treatment lines, summarize the highest line only
- Best response, which is derived from the highest treatment line

Listing of prior chemotherapy history (including treatment lines, start date, end date, chemotherapy regimens, number of treatments, best response, progression date, treatment types), prior anti-cancer radiotherapy (including radiotherapy sites, start date, end date, overall radiotherapy dose, best response) and prior anti-cancer surgery (including surgery name and surgery date) will be provided.

8.1.6. Medical History

Medical history will be coded using version 22.0 or above of Medical Dictionary for Regulatory Activities (MedDRA) and will be summarized by treatment group and overall for ITT from the 'Past History' eCRF page. Medical history will be summarized as the number and percentage of patients by MedDRA preferred term (PT) as event category and MedDRA primary system organ class (SOC) as summary category. Each patient will be counted only once within each PT or SOC.

Data listing of medical history will be provided.

8.1.7. Concomitant Medications

The concomitant medications are collected under the 'Therapeutic Medication' eCRF pages.

Concomitant medications are medications, other than study medications, which started prior to first dose date of study treatment and continued on on-treatment period as well as those started during the on-treatment period.

Summary of concomitant medications will include the number and percentage of patients in each treatment group and overall for SS by Anatomical Therapeutic Chemical (ATC) Classification level 2 and preferred term.

Data listing of concomitant medications will be provided.

8.2. Efficacy Analyses

8.2.1. Analysis of Primary Efficacy Endpoints

ITT will be used as the primary analysis set for the primary efficacy analysis.

The primary endpoint is investigator evaluated PFS based on RECIST v1.1. PFS is defined as the time from randomization to the date of objective disease progression or any cause of death, which occurred earlier.

PFS (months)= [date of censoring/event-date of randomization+1]/30.4375

Censoring rules for primary analysis of PFS is presented as below:

Case number	Description	Censored/ event	Date of censor/event
1	No baseline or post-baseline tumor assessment and no early death (< 117 days from randomization)	Censored	Randomization date
2	No baseline or post-baseline tumor assessment with early death (< 117 days from randomization)	Event	Death date
3	≤ 1 missing of tumor assessment before PD or death	Event	PD or death date, which occurs earlier
4	2 or more consecutive missing of tumor assessment before PD or death	Censored	Last adequate tumor assessment date before consecutive missing of tumor assessment

Hypothesis testing for a potential inequivalent claim in regard to PFS will be performed with a 2-sided hypotheses setting.

Suppose that SA(t) and SP(t) are the cumulative survival functions for PFS in test treatment and control groups, respectively. The null hypothesis

H_0: SA(t) = SP(t) for all $t \geq 0$ (identical survival functions for both treatment groups) will be tested against the 2-sided alternative hypothesis

H_a: SA(t) ≠ SP(t) for at least some $t > 0$

8.2.1.1. Main analysis of the primary efficacy endpoint

The Kaplan-Meier method will be used to estimate the distribution of PFS (including the median PFS) for each treatment group, the 95% confidence intervals of mPFS will be calculated by using the Brookmeyer and Crowley method. The corresponding Kaplan-Meier curves will also be presented for each treatment group.

The stratified log-rank test will be conducted via use of SAS PROC LIFETEST to detect the difference in PFS between the test treatment and control group. The stratified Cox proportional hazards regression model will be used via SAS PROC PHREG to estimate the hazard ratio and associated 95% confidence interval. The randomization stratification factors, including previous platinum-sensitive relapsed (yes vs. no) and platinum-free interval (≤3 months vs. 3-6 months), will be included in the stratified log-rank test and stratified Cox proportional hazards regression model as strata.

8.2.1.2. Sensitivity analysis of the primary efficacy endpoint

The following sensitivity analyses will be performed to assess the robustness of the main analysis:

- The unstratified log-rank test will be conducted to detect the difference in PFS. The unstratified Cox proportional hazards regression model will be used to estimate the hazard ratio and associated 95% confidence interval.
- The second sensitivity analysis is the same as the primary analysis except it considers initiation of an anti-cancer therapy before PD or death, which will be censored at the last adequate tumor assessment date before the initiation of new anti-cancer therapy.

Censoring rules for the second sensitivity analysis of PFS is presented as below:

Case number	Description	Censored/ event	Date of censor/event
1	No baseline or post-baseline tumor assessment and no early death (< 117 days from randomization)	Censored	Randomization date
2	No baseline or post-baseline tumor assessment with early death (< 117 days from randomization)	Event	Death date
3	PD or death without the initiation of new anti-cancer therapy except for case 5	Event	PD or death date, which occurs earlier

4	Initiated new anti-cancer therapy before PD or death	Censored	Last adequate tumor assessment date before the initiation of new anti-cancer therapy
5	2 or more consecutive missing of tumor assessment before PD or death	Censored	Last adequate tumor assessment date before consecutive missing of tumor assessment

8.2.1.3. Subgroup analysis

In addition, subgroup analysis will be performed for the baseline factors listed in section 7.3.4. The HR for apatinib plus PLD group versus PLD group and associated 95% confidence interval will be estimated by Cox proportional hazards model stratified by the randomization strata (previous platinum-sensitive relapsed (yes vs. no) and platinum-free interval (≤3 months vs. 3-6 months)) in each level of the subgroups.

PFS relevant information will be listed for all subjects in the ITT.

8.2.2. Analysis of Secondary Efficacy Endpoints

8.2.2.1. Overall survival

The primary analysis of Overall survival (OS) is based on ITT. OS is defined as from the date of randomization to the date of death. If a subject lost to follow-up or withdrawal from study before death is observed, the subject will be censored at the last date known to be alive.

Similar analysis of PFS will be performed for OS. The Kaplan-Meier method will be used to estimate the distribution of OS (including the median OS) for each treatment group, the 95% confidence intervals of mOS will be calculated by using the Brookmeyer and Crowley method. The corresponding Kaplan-Meier curves will also be presented.

The stratified log-rank test will be conducted via use of SAS PROC LIFETEST to detect the difference in OS between the test treatment and control group. The stratified Cox proportional hazards regression model will be used via SAS PROC PHREG to estimate the hazard ratio and associated 95% confidence interval. The randomization stratification factors, including previous platinum-sensitive relapsed (yes vs. no) and platinum-free interval (≤3 months vs. 3-6 months), will be included in the stratified log-rank test and stratified Cox proportional hazards regression model as strata.

The following sensitivity analysis will be performed to assess the robustness of the main analysis:

- Use unstratified log-rank test to detect the difference in OS and use unstratified Cox regression model to estimate hazard ratio and associated 95% CI.

Subgroup analysis will be performed for the baseline factors listed in section 7.3.4. The HR for apatinib plus PLD group versus PLD group and associated 95% confidence interval will be estimated by Cox proportional hazards model stratified by the randomization strata (previous platinum-sensitive relapsed (yes vs. no) and platinum-free interval (≤3 months vs. 3-6 months)) in each level of the subgroups.

OS relevant information will be listed for all subjects in the ITT.

8.2.2.2. Objective response rate (ORR) and Disease control rate (DCR)

Objective response rate (ORR) will be analyzed primarily based on mITT. ORR is defined as the proportion of subjects with best overall response (BOR) of complete response (CR) or partial response (PR) according to RECIST v1.1 in the corresponding analysis set.

Disease control rate (DCR) will be analyzed primarily based on mITT. DCR is defined as the proportion of subjects with best overall response (BOR) of complete response (CR), partial response (PR) or stable disease (SD) according to RECIST v1.1 in the corresponding analysis set.

SD should meet the minimum duration of 6 weeks (42 days) from randomization.

The number and proportion of subjects with BOR of CR, PR, SD, PD and NE in each treatment group will be presented.

The 95% confidence interval of ORR and DCR will be estimated by using Clopper-Pearson method. The stratified Cochran-Mantel-Haenszel (CMH) test with previous platinum-sensitive relapsed (yes vs. no) and platinum-free interval (≤3 months vs. 3-6 months) as stratification factors will be used to compare the ORR and DCR between the test treatment and control group.

Unconfirmed response results will be used for the main analysis, while the confirmed response results are sensitivity analysis. The confirmation rule of BOR could be referred to APPENDIX. Confirmed CR and PR need to be confirmed at least 4 weeks (28 days) apart.

In addition, baseline target lesion burden and best post-baseline tumor shrinkage will be summarized by descriptive statistics. Waterfall plot presenting best post-baseline tumor shrinkage will also be provided for each treatment group.

Data listing regarding BOR will be provided for subjects in mITT.

8.2.2.3. Duration of Response (DoR) and Time to Response (TTR)

Duration of response (DoR) is defined as first time of CR or PR, to PD or death which occurs earlier. The analysis is only applicable for subjects whose BOR is CR or PR. The censor rule is the same as PFS.

Time to response (TTR) is defined as the time from randomization to the first time of CR or PR. The analysis is only applicable for subjects whose BOR is CR or PR.

The analysis methods of DoR and TTR is similar to those of PFS and OS.

The analysis will be based on subjects whose BOR is CR or PR in ITT.

The Kaplan-Meier method will be used to estimate the distribution of DoR and TTR (including the median DoR and median TTR) for each treatment group, the 95% confidence intervals of mDoR and mTTR will be calculated by using the Brookmeyer and Crowley method. The corresponding Kaplan-Meier curves will also be presented.

The stratified log-rank test will be conducted via use of SAS PROC LIFETEST to detect the difference in DoR and TTR between the test treatment and placebo group. The stratified Cox proportional hazards regression model will be used via SAS PROC PHREG to estimate the hazard ratio and associated 95% confidence interval. The randomization stratification factors, including previous platinum-sensitive relapsed (yes vs. no) and platinum-free interval (≤3 months vs. 3-6 months), will be included in the stratified log-rank test and stratified Cox proportional hazards regression model as strata.

In addition, analyze DoR and TTR with confirmed CR or PR in the same way as unconfirmed CR/PR.

Data listing regarding DoR and TTR (including the subject ID, randomization date, first date of CR/PR, event or censor date as well as the events type and censor reason correspondingly) will also be provided.

8.3. Safety Analyses

All the safety analysis will be performed for SS except for otherwise specified.

Safety endpoints include treatment-emergent adverse events (TEAE), clinical laboratory tests, vital signs, electrocardiogram (ECG) parameters, echocardiogram (ECHO) parameters and Eastern Cooperative Oncology Group performance status (ECOG PS).

8.3.1. Dosing and Extent of Exposure

The following items reflecting drug exposure will be summarized by treatment group for apatinib and PLD respectively based on SS:

Apatinib:

- Duration of exposure (cycle) = (last dose date of apatinib – first dose date of apatinib + 1)/28
- Duration of exposure (day) = (last dose date of apatinib – first dose date of apatinib + 1)
- Actual duration of exposure (cycle) = [(last dose date of apatinib – first dose date of apatinib + 1) – sum of dose interruption days of apatinib]/7

- Total dose (mg) = sum of dose of apatinib per day
- Dose intensity (mg/day) = Total dose (mg)/ Duration of exposure (day)
- Relative dose intensity (%)= Dose intensity (mg/day)*100/ planned dose per day (250 mg/day)

PLD (each cycle for PLD is defined by a 4- week period):

- Duration of exposure (cycles) = (last dose date of PLD – first dose date of PLD + 28)/ 28
- Actual duration of exposure (cycles) = number of cycles with non-zero dose of PLD
- Total dose (mg/m^2) = sum of dose of PLD per cycle (mg)/ BSA (m^2)
- Dose intensity (mg/cycle/m^2) = Total dose (mg)/ Duration of exposure (cycle) * BSA (m^2)
- Relative dose intensity (%)= Dose intensity (mg/cycle/m^2)*100/ planned dose per cycle (40 mg/cycle/m^2)

In addition, number and percentage of subjects who did not experience dose reduction or experienced dose reduction for at least 1 time will be summarized for each treatment group. Similarly, number and percentage of subjects who did not experience drug interruption or experienced drug interruption for at least1 time drug interruption will be summarized for each treatment group.

8.3.2. Adverse Events

Adverse events (AE) will be coded using Version 22.0 or above of the Medical Dictionary for Regulatory Activities (MedDRA) and classified according to the NCI-CTCAE criteria version 4.0. AE will be summarized by treatment group.

- An AE will be considered as a TEAE if it occurs or becomes worse in severity after the initiation of study treatment and within 30 days after last study treatment administration.

If the CTCAE grade of an AE is missing, it will be considered as grade 3 AE.

The TEAEs which being recorded as being “definitely related with”, “highly-possibly related with” and “possibly related with” study drug in eCRF will be considered as drug related TEAE (TRAE). If the relationship to the study treatment is missing, it will be considered to be related to study treatment.

A high level summary of the number of subjects with TEAEs will be presented by treatment group, including the number and percentage of subjects with:

- Any TEAEs
- TEAEs with CTCAE grade ≥ 3
- TEAE leading to dose reduction of apatinib or PLD
- TEAE leading to interruption of apatinib or PLD

- TEAE leading to permanent discontinuation of apatinib or PLD
- TEAE leading to death
- Serious TEAE (TESAE)
- Study treatment related TEAEs (TRAEs) (related to apatinib or PLD)
- TRAEs (related to apatinib or PLD) with CTCAE grade ≥ 3
- TRAEs (related to apatinib or PLD) leading to dose reduction of apatinib or PLD
- TRAEs (related to apatinib or PLD) leading to interruption of apatinib or PLD
- TRAE (related to apatinib or PLD) leading to permanent discontinuation of apatinib
- TRAE (related to apatinib or PLD) leading to permanent discontinuation of PLD
- TRAEs (related to apatinib or PLD) leading to death
- Serious TRAEs (related to apatinib or PLD)

The number and percentage of subjects reporting TEAEs in each treatment group will be summarized overall, by system organ class (SOC) and preferred term (PT) and further be summarized by severity (overall and for CTCAE grade ≥ 3). A subject who reports more than 1 TEAEs with the same SOC/PT will only be counted once at the corresponding SOC/PT by using the most severe CTCAE grade.

In addition, TEAE with incidence rate ≥10% will also be summarized by PT.

Data listing will be provided.

8.3.2.1. Treatment-related TEAEs

The number and percentage of subjects reporting TRAEs in each treatment group will be summarized overall by SOC/PT and further be summarized by severity (overall and for CTCAE grade ≥ 3). Only the occurrence with the highest CTCAE grade and the closest relationship to the study treatment will be counted at the corresponding SOC/PT level if a subject who reports more than 1 TRAEs with different CTCAE grade and relationship to the study treatment which could be coded to the same SOC/PT.

In addition, TRAEs with incidence rate ≥10% will also be summarized by PT.

Data listing of TRAEs will be provided with the information of subject ID, AE name, start/end date, severity, CTCAE grade, relationship to the study drug, outcome, SAE or not, the action taken for AE and whether it has led to the withdrawal from study.

8.3.2.2. Serious adverse events

SAE will be summarized in the similar way of TEAE. The number and percentage of subjects reporting SAEs in each treatment group will be summarized overall by SOC/PT and further be summarized by severity (overall and for CTCAE grade ≥ 3).

Treatment related SAEs will be summarized in the similar way of SAEs.

Data listing of SAE will be listed.

8.3.2.3. TEAE leading to study drug interruption, dose reduction and discontinuation

The number and percentage of subjects reporting TEAEs which lead to study drug interruption, dose reduction and drug discontinuation in each treatment group will be summarized overall by SOC/PT and further be summarized by severity (overall and for CTCAE grade ≥ 3). A subject who reports more than 1 TEAEs with the same SOC/PT will only be counted once at the corresponding SOC/PT by using the most severe CTCAE grade.

TRAEs which lead to study drug interruption, dose reduction and drug discontinuation in each treatment group will be summarized in the similar way of TEAEs.

Data listing of TEAEs leading to study drug interruption, dose reduction and drug discontinuation will be listed.

8.3.2.4. Death, Other Serious Adverse Events and Other Significant Adverse Events

The number and percentage of TEAEs and TRAEs leading to death will be summarized will be summarized by SOC/PT for each treatment group.

In addition, the time to the first occurrence of TEAEs leading to death will be provided.

8.3.3. Clinical Laboratory Evaluations

The clinical laboratory evaluation system and parameters listed below will be summarized if applicable.

Table 1: List of Laboratory Tests

Hematology	hemoglobin, white blood cell (WBC) count, absolute neutrophil count (ANC) and platelet (PLT) count
Urinalysis	urinary pH, urine protein, urine red blood cells and urine white blood cell
Biochemistry	liver function tests (ALT, AST and TBIL), renal function tests (BUN and Cr) and electrolyte tests (serum potassium, sodium and chloride)
Coagulation	prothrombin time

The abnormality for each laboratory parameter will be assessed by investigator. A shift table from baseline to the worst post-baseline assessment according to the investigator's evaluation for each parameter listed above(if applicable) will be presented by treatment group.

Data listing for the abnormal laboratory tests results as well as the stool blood assessment results will be presented.

8.3.4. Vital Sign

The results and their changes from baseline of each vital sign parameter, including height (cm), weight (kg), BSA (m^2), temperature (℃), heart rate (beats/min), breath (breaths/min), systolic blood pressure (mmHg) and diastolic blood pressure (mmHg), will be summarized by descriptive statistics for baseline and each post-baseline scheduled visits as well as corresponding changes from baseline (if applicable) by study treatment.

Data listing will be provided for the vital sign results.

8.3.5. ECG and Echocardiogram

The quantitative results and their changes from baseline of each ECG parameter, including heart rate (beats/min), QTc (ms) and LVEF (%), will be summarized by descriptive statistics for baseline (changes from baseline is not applicable) and each post-baseline scheduled visits by treatment group.

The abnormality of ECG and Echocardiogram will be assessed by investigator. A shift table from baseline to the worst post-baseline assessment according to the investigator's evaluation will be presented for ECG and Echocardiogram by treatment group.

Data listing will be provided for the ECG and Echocardiogram results.

8.3.6. Eastern Cooperative Oncology Group (ECOG) PS

The analysis of ECOG will be performed based on ITT. The ECOG PS will be summarized as categorical variables for baseline and each post-baseline scheduled visit by treatment group. In addition, the shift table from baseline to the worst score will be provided.

Data listing will be provided for the ECOG PS.

8.4. Patients Reported Outcome (PRO) Analysis

Health-related quality of life (HRQoL) will be assessed by the European Organization for Research and Treatment of Cancer Quality of Life Questionnaire-Core 30 (EORTC QLQ-C30). The EORTC QLQ-C30 consists of 30 questions which assess five functional scales (physical, role, cognitive, emotional and social), global health status/quality of life scale, three symptom scales (fatigue, pain, nausea and vomiting) and six single items (dyspnoea, insomnia, appetite loss, constipation, diarrhea and financial difficulties).

The subscales of the EORTC QLQ-C30 will be scored based on the EORTC scoring manual. In summary, each scale will be transformed so that scale scores will range from 0 to 100. The transformation will proceed in two steps.

1. The average of the items contributing subscale will be calculated to compute the raw score (RS) of the scale

   RS=(Q1+Q2+…+Qn)/n, in which n is the number of item in the scale.

2. A linear transformation will be applied to ‘standardize’ the raw score

   Functional scales: standard score =[1-(RS-1)/R]*100

   Symptom scales/ items and general health status/ QoL: standard score =[(RS-1)/R]*100

   (where R is the full range of scores for each domain or item)

After scores are transformed, high scores will represent higher (“better”) levels of functioning and the general health status or a higher (“worse”) level of symptoms. Response for the 30 items will be summarized as categorical variable for baseline and each post-baseline scheduled visit by treatment group. Scale scores will be summarized as continues variable for baseline and each post-baseline scheduled visit by treatment group.

9. SUMMARY OF CHANGES TO THE STATISTICAL ANALYSES SPECIFIED IN PROTOCOL

- Add DoR and TTR as secondary endpoints and their analysis methods
- Set EORTC QLQ-C30 as a separate health quality outcome and add the analysis of the quality of life

10. REFERENCES

1. Nishida N, Yano H, Komai K, Nishida T, Kamura T, Kojiro M. Vascular endothelial growth factor C and vascular endothelial growth factor receptor 2 are related closely to the prognosis of patients with ovarian carcinoma. Cancer 2004; 101(6): 1364-74.
2. Poveda AM, Selle F, Hilpert F, et al. Bevacizumab Combined With Weekly Paclitaxel, Pegylated Liposomal Doxorubicin, or Topotecan in Platinum-Resistant Recurrent Ovarian Cancer: Analysis by Chemotherapy Cohort of the Randomized Phase III AURELIA Trial. J Clin Oncol 2015; 33(32): 3836-8.
3. Naumann RW, Coleman RL, Burger RA, et al. PRECEDENT: a randomized phase II trial comparing vintafolide (EC145) and pegylated liposomal doxorubicin (PLD) in combination versus PLD alone in patients with platinum-resistant ovarian cancer. J Clin Oncol 2013; 31(35): 4400-6.
4. Mutch DG, Orlando M, Goss T, et al. Randomized phase III trial of gemcitabine compared with pegylated liposomal doxorubicin in patients with platinum-resistant ovarian cancer. J Clin Oncol 2007; 25(19): 2811-8.
5. Common Terminology Criteria for Adverse Events (CTCAE). Version 4.0. United States Department of Health and Human Services, National Institutes of Health, National Cancer Institute, Washington, DC, USA, May 28, 2009.
6. Eisenhauer EA, Therasse P, Bogaerts J Schwartz LH, Sargent D, Ford R, et al. New response evaluation criteria in solid tumours: Revised RECIST guideline (version 1.1). Eur J Cancer. 2009;45:228-47.
7. NMPA. Guidance for clinical trial data management and report & statistical analysis plan and report. 2016.
8. ICH E9: Statistical Principles for ClinicalTrials. 1998.
9. Fayers PM, Aaronson NK, Bjordal K, Groenvold M, Curran D, Bottomley A on behalf of the EORTC Quality of Life Group. EORTC QLQ-C30 Scoring Manual (3rd edition). Brussels: EORTC, 2001.

11. APPENDICES

Appendix 1. Time point response: patients with target (+/- non-target) disease

Target lesions	Nontarget lesions	New lesions	Overall response
CR	CR	No	CR
CR	non-CR/non-PD	No	PR
CR	Not fully evaluable	No	PR
PR	non-PD or not fully evaluable	No	PR
SD	non-PD or not fully evaluable	No	SD
Not fully evaluable	non-PD	No	NE
PD	Any	Yes or no	PD
Any	PD	Yes or no	PD
Any	Any	Yes	PD

CR = complete response; PR = partial response; SD = stable disease; PD = progressive disease; NE =non-evaluable

Appendix 2. Time point response: patients with non-target disease only

Nontarget lesions	New lesions	Overall response
CR	No	CR
Non-CR or non-PD	No	Non-CR or non-PD
Not fully evaluable	No	NE
Unconfirmed PD	Yes or no	PD
Any	Yes	PD

Appendix 3. BOR confirmation rule

Overall response at the first time point	Overall response at the subsequent time point	Best overall response
CR	CR	CR
CR	PR	SD， PD or PR[a]
CR	SD	SD provided minimum criteria for SD duration met, otherwise, PD
CR	PD	SD provided minimum criteria for SD duration met, otherwise, PD
CR	NE	SD provided minimum criteria for SD duration met, otherwise, NE
PR	CR	PR
PR	PR	PR
PR	SD	SD
PR	PD	SD provided minimum criteria for SD duration met, otherwise, PD
PR	NE	SD provided minimum criteria for SD duration met, otherwise, NE
NE	NE	NE

Note: CR = complete response, PR = partial response, SD = stable disease, PD = progressive disease, NE = not evaluable.

Superscript "a": If a CR is truly met at first time point, then any disease seen at a subsequent time point, even disease meeting PR criteria relative to baseline, makes the disease PD at that point (since disease must have reappeared after CR). Best response would depend on whether minimum duration for SD was met. However, sometimes ‘CR’ may be claimed when subsequent scans suggest small lesions were likely still present and in fact the patient had PR, not CR at the first time point. Under these circumstances, the original CR should be changed to PR and the best response is PR. The minimum SD duration is 6 weeks (42 days).

Appendix 4. SAS code for survival analysis

Kaplan-Meier method:

```
Proc lifetest data=dataset_name;
Time time*censor(0);
Strata strata1 strata2 /Group=TREATMENT;
Run;
```

Cox proportional hazards regression model:

```
Proc phreg data=dataset_name;
model time*censor(0)=TREATMENT;
Strata strata1 strata2;
Run;
```

Strata1= previous platinum-sensitive relapsed (yes vs. no)

Strata2= platinum-free interval (⩽3 months vs. 3-6 months)
