# Supplementary material for: Effect of Apatinib Plus Pegylated Liposomal Doxorubicin vs Pegylated Liposomal Doxorubicin Alone on Platinum-Resistant Recurrent Ovarian Cancer: The APPROVE Randomized Clinical Trial
Source: JAMA Oncol. 2022 Jun 30;8(8):1169–76. doi: 10.1001/jamaoncol.2022.2253 (PMC9247861; doi:10.1001/jamaoncol.2022.2253)
Supplement: Supplement 3. — eTable 1. List of 11 participating sites eTable 2. Treatment-emergent adverse events eFigure. Subgroup analyses of progression-free survival [file jamaoncol-e222253-s003.pdf]

Supplemental Online Content

Wang T, Tang J, Yang H, et al. Effect of apatinib plus pegylated liposomal doxorubicin vs pegylated liposomal doxorubicin alone on platinum-resistant recurrent ovarian cancer: the APPROVE randomized clinical trial. *JAMA Oncol*. Published online June 30, 2022. doi:10.1001/jamaoncol.2022.2253

eTable 1. List of 11 participating sites
eTable 2. Treatment-emergent adverse events
eFigure. Subgroup analyses of progression-free survival

This supplemental material has been provided by the authors to give readers additional information about their work.

© 2022 Wang T et al. *JAMA Oncology.*

eTable 1. List of 11 participating sites.

Site name	Number of recruited patients
National Cancer Center/National Clinical Research Center for Cancer/Cancer Hospital, Chinese Academy of Medical Sciences and Peking Union Medical College	73
Hunan Cancer Hospital	20
Yunnan Provincial Cancer Center	17
West China Second University Hospital	12
Cancer Hospital of China Medical University	11
Chongqing University Cancer Hospital	8
The First Hospital of Jilin University	5
Xiangya Hospital	2
Guangxi Medical University Affiliated Tumor Hospital	2
The Second Affiliated Hospital of Dalian Medical University	1
Hubei Cancer Hospital	1

© 2022 Wang T et al. *JAMA Oncology.*

eTable 2. Treatment-emergent adverse events

Events, No. (%)	Apatinib plus PLD group (*n* = 74)				PLD group (*n* = 72)			
	**All Grades**	**Grade 1-2**	**Grade 3**	**Grade 4**	**All Grades**	**Grade 1-2**	**Grade 3**	**Grade 4**
Any	69 (93.2%)	37 (50.0%)	30 (40.5%)	2 (2.7%)	61 (84.7%)	47 (65.3%)	14 (19.4%)	0 (0%)
WBC decrease	45 (60.8%)	40 (54.1%)	5 (6.8%)	0 (0%)	36 (50.0)	33 (45.8%)	3 (4.2%)	0 (0%)
Neutrophil count decrease	44 (59.5%)	33 (44.6%)	11 (14.9%)	0 (0%)	27 (37.5%)	21 (29.2%)	6 (8.3%)	0 (0%)
Oral ulcer	21 (28.4%)	20 (27.0%)	1 (1.4%)	0 (0%)	9 (12.5%)	7 (9.7%)	2 (2.8%)	0 (0%)
Hand-foot syndrome	19 (25.7%)	15 (20.3%)	3 (4.1%)	1 (1.4%)	2 (2.8%)	0 (0%)	2 (2.8%)	0 (0%)
Anaemia	16 (21.6%)	15 (20.3%)	1 (1.4%)	0 (0%)	16 (22.2%)	13 (18.1%)	3 (4.2%)	0 (0%)
Fatigue	15 (20.3%)	15 (20.3%)	0 (0%)	0 (0%)	8 (11.1%)	8 (11.1%)	0 (0%)	0 (0%)
Nausea	14 (18.9%)	13 (17.6%)	1 (1.4%)	0 (0%)	19 (26.4%)	19 (26.4%)	0 (0%)	0 (0%)
Hypertension	13 (17.6%)	7 (9.5%)	6 (8.1%)	0 (0%)	1 (1.4%)	1 (1.4%)	0 (0%)	0 (0%)
Oral mucositis	12 (16.2%)	10 (13.5%)	1 (1.4%)	1 (1.4%)	6 (8.3%)	6 (8.3%)	0 (0%)	0 (0%)
Platelet count decrease	12 (16.2%)	10 (13.5%)	2 (2.7%)	0 (0%)	3 (4.2%)	3 (4.2%)	0 (0%)	0 (0%)
Vomiting	11 (14.9%)	11 (14.9%)	0 (0%)	0 (0%)	10 (13.9%)	10 (13.9%)	0 (0%)	0 (0%)
AST increase	11 (14.9%)	11 (14.9%)	0 (0%)	0 (0%)	2 (2.8%)	2 (2.8%)	0 (0%)	0 (0%)
Dizziness	11 (14.9%)	10 (13.5%)	1 (1.4%)	0 (0%)	2 (2.8%)	2 (2.8%)	0 (0%)	0 (0%)
Headache	11 (14.9%)	10 (13.5%)	1 (1.4%)	0 (0%)	3 (4.2%)	3 (4.2%)	0 (0%)	0 (0%)
Proteinuria	11 (14.9%)	9 (12.2%)	2 (2.7%)	0 (0%)	2 (2.8%)	2 (2.8%)	0 (0%)	0 (0%)
Urine leucocyte positive	8 (10.8%)	8 (10.8%)	0 (0%)	0 (0%)	2 (2.8%)	2 (2.8%)	0 (0%)	0 (0%)

AST, aspartate aminotransferase; PLD, pegylated liposomal doxorubicin; WBC, white blood cell count. Treatment-emergent adverse events occurring in 10% or more of patients in either group are listed.

© 2022 Wang T et al. *JAMA Oncology.*

eFigure. Subgroup Analyses of Progression-Free Survival.

Groups	Apatinib+PLD (n=78) Event/N	PLD (n=74) Event/N	Apatinib+PLD Median PFS (95% CI)	PLD Median PFS (95% CI)	HR (95% CI)
**Age**					
<65	30/67	46/68	5.8 (3.7,8.8)	3.4 (2.5,3.8)	0.48 (0.29,0.82)
≥65	7/11	4/6	8.8 (2.2,12.7)	2.1 (0.9,NA)	0.11 (0.01,1.13)
**ECOG performance status**					
0	10/17	12/24	5.7 (3.2,9.9)	6.5 (3.4,NA)	0.87 (0.30,2.50)
1-2	27/59	35/46	5.8 (3.7,8.9)	2.6 (2.0,3.7)	0.28 (0.16,0.50)
**Previous platinum-sensitive relapse**					
No	25/49	35/43	6.0 (3.7,8.8)	2.6 (2.0,3.4)	0.35 (0.19,0.62)
Yes	12/29	15/31	4.0 (3.5,12.7)	4.6 (2.0,8.1)	0.76 (0.34,1.70)
**Previous therapy lines**					
1	24/44	27/38	6.0 (3.7,8.9)	2.9 (2.0,3.9)	0.40 (0.21,0.75)
2	5/18	16/26	5.8 (2.2,7.3)	3.4 (2.0,5.9)	0.37 (0.10,1.41)
≥3	8/16	7/10	4.0 (2.9,NA)	2.9 (0.6,5.3)	0.69 (0.21,2.24)
**Platinum-free interval (months)**					
>3	22/44	27/45	4.0 (3.6,5.8)	3.7 (2.0,5.6)	0.74 (0.41,1.33)
≤3	15/34	23/29	8.8 (3.7,11.8)	2.5 (2.0,3.7)	0.20 (0.09,0.44)
**Ascites**					
No	20/48	26/46	7.8 (4.6,9.9)	3.7 (2.0,6.5)	0.33 (0.16,0.67)
Yes	17/30	24/28	3.7 (2.9,8.9)	3.3 (2.0,3.7)	0.51 (0.25,1.00)
**Platinum-refractory**					
No	31/63	41/60	5.7 (3.7,8.8)	3.4 (2.1,3.9)	0.51 (0.31,0.87)
Yes	6/15	9/14	7.3 (3.7,12.7)	3.0 (1.7,3.7)	0.16 (0.04,0.61)
**First platinum-resistant relapse**					
No	8/17	14/18	5.8 (3.6,NA)	2.9 (1.7,3.7)	0.37 (0.13,1.05)
Yes	29/61	36/56	5.7 (3.7,8.8)	3.4 (2.1,4.6)	0.48 (0.27,0.83)
**Overall**	37/78	50/74	5.8 (3.8,8.8)	3.3 (2.1,3.8)	0.44 (0.28,0.71)

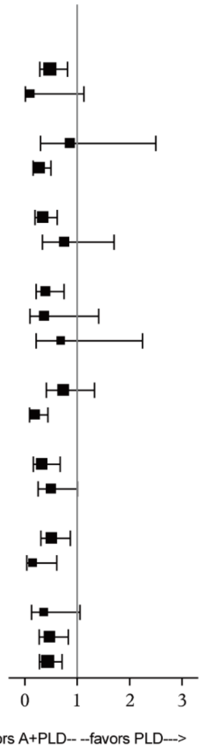

© 2022 Wang T et al. *JAMA Oncology.*
