# Supplementary material for: Effect of Apatinib Plus Pegylated Liposomal Doxorubicin vs Pegylated Liposomal Doxorubicin Alone on Platinum-Resistant Recurrent Ovarian Cancer: The APPROVE Randomized Clinical Trial
Source: JAMA Oncol. 2022 Jun 30;8(8):1169–76. doi: 10.1001/jamaoncol.2022.2253 (PMC9247861; doi:10.1001/jamaoncol.2022.2253)
Supplement: Supplement 4. — Data sharing statement [file jamaoncol-e222253-s00.pdf]

Data Sharing Statement

Wang. Effect of Apatinib Plus Pegylated Liposomal Doxorubicin vs Pegylated Liposomal Doxorubicin Alone on Platinum-Resistant Recurrent Ovarian Cancer. *JAMA Oncol*. Published June 30, 2022. doi:10.1001/jamaoncol.2022.2253

Data
Data available: No
